# Genome-wide identification of Gramineae histone modification genes and their potential roles in regulating wheat and maize growth and stress responses

**DOI:** 10.1186/s12870-021-03332-8

**Published:** 2021-11-20

**Authors:** Liwei Zheng, Shengjie Ma, Dandan Shen, Hong Fu, Yue Wang, Ying Liu, Kamran Shah, Caipeng Yue, Jinyong Huang

**Affiliations:** 1 School of Agricultural Sciences, Zhengzhou University, Henan, 450001 China; 2 College of Horticulture, Northwest A & F University, Yangling, 712100 Shaanxi China

**Keywords:** Histone modification, Wheat and maize, Growth and development, Stress

## Abstract

**Background:**

In plants, histone modification (*HM*) genes participate in various developmental and defense processes. Gramineae plants (e.g., *Triticum aestivum*, *Hordeum vulgare*, *Sorghum bicolor*, *Setaria italica*, *Setaria viridis*, and *Zea mays*) are important crop species worldwide. However, little information on *HM* genes is in Gramineae species.

**Results:**

Here, we identified 245 *TaHMs*, 72 *HvHMs*, 84 *SbHMs*, 93 *SvHMs*, 90 *SiHMs*, and 90 *ZmHMs* in the above six Gramineae species, respectively. Detailed information on their chromosome locations, conserved domains, phylogenetic trees, synteny, promoter elements, and gene structures were determined. Among the *HMs*, most motifs were conserved, but several unique motifs were also identified. Our results also suggested that gene and genome duplications potentially impacted the evolution and expansion of *HMs* in wheat. The number of orthologous gene pairs between rice (*Oryza sativa*) and each Gramineae species was much greater than that between *Arabidopsis* and each Gramineae species, indicating that the dicotyledons shared common ancestors. Moreover, all identified *HM* gene pairs likely underwent purifying selection based on to their non-synonymous (Ka)/synonymous (Ks) nucleotide substitutions. Using published transcriptome data, changes in *TaHM* gene expression in developing wheat grains treated with brassinosteroid, brassinazole, or activated charcoal were investigated. In addition, the transcription models of *ZmHMs* in developing maize seeds and after gibberellin treatment were also identified. We also examined plant stress responses and found that heat, drought, salt, insect feeding, nitrogen, and cadmium stress influenced many *TaHMs*, and drought altered the expression of several *ZmHMs*. Thus, these findings indicate their important functions in plant growth and stress adaptations.

**Conclusions:**

Based on a comprehensive analysis of Gramineae *HMs*, we found that *TaHMs* play potential roles in grain development, brassinosteroid- and brassinazole-mediated root growth, activated charcoal-mediated root and leaf growth, and biotic and abiotic adaptations. Furthermore, *ZmHMs* likely participate in seed development, gibberellin-mediated leaf growth, and drought adaptation.

**Supplementary Information:**

The online version contains supplementary material available at 10.1186/s12870-021-03332-8.

## Background

In plants, epigenetic histone modification (HM) can activate or silence gene expression. *HM* genes play essential functions in various growth and development processes and stress responses, such as carotenoid biosynthesis, floral organ development, and fungal pathogen resistance [1–3]. In plants, HM depends on four kinds of enzymes, including histone methyltransferases (HMTs), histone demethylases (HDMs), histone acetylases (HATs), and histone deacetylases (HDACs) [4–7].

HMTs are mainly encoded by the *SET DOMAIN GROUP* (*SDG*) and *protein arginine methyltransferases* (*PRMTs*) genes [8]. Plant *HMT* genes are involved in shoot and root branching, hormone regulation, morphogenesis, circadian cycle, fungal pathogen resistance, and abscisic acid (ABA) and salt stress [9, 10]. Furthermore, in plants, HMT-mediated processes can be reversed by activation of *HDM* genes. The *HDM* gene family contains two gene subfamilies, i.e., *SWIRM and C-terminal domain* (*HDMA*) and *JmjC domain-containing proteins* (*JMJ*) [11]. Studies on *HDMs* in plants have revealed their functions in chromatin regulation, brassinosteroid (BR) signaling, floral induction, pollen development, floral organ formation, and circadian cycle [12, 13]. Four types of genes (*HAGs*, *HAMs*, *HACs*, and *HAFs*) are recognized in the *HAT* gene family [14]. *HAT* genes participate in the transition from vegetative to reproductive growth, abiotic and biotic responses, and stress-related hormone signaling [15–18]. The *HDAC* family contains the *RPD3/HDA1* (*HDA*), *Silent Information Regulator 2* (*SRT*), and *HD2* (*HDT*) subfamilies [19]. *HDAC* genes participate in vegetative and reproductive growth, stress adaptations, gene silencing, cell growth, and regeneration [20, 21].

Gramineous grain crops, including *Triticum aestivum*, *Hordeum vulgare*, *Sorghum bicolor*, *Setaria italica*, *Setaria viridis*, and *Zea mays*, are widely cultivated and provide important caloric intake for humans [22]. In Gramineae species, growth and development are closely related to grain yield and quality [23, 24]. Biotic and abiotic stresses markedly affect crop development and yield [25–30]. Although the functions of *HMs* in plant growth and environmental adaptations have been identified in some plant species [8, 18, 20], their characteristics and functions in *T. aestivum*, *H. vulgare*, *S. bicolor*, *S. viridis*, *S. italica*, and *Z. mays* remain unclear. The publication of the genomes of these species allows for the systematic characterization of *HM* genes via bioinformatics analysis.

In this study, 245, 72, 84, 93, 90, and 90 *HMs* were identified in the *T. aestivum*, *H. vulgare*, *S. bicolor*, *S. viridis*, *S. italica*, and *Z. mays* genomes, respectively. Their location on chromosomes, conserved domains, evolution, synteny, promoter sequences, and gene structures were analyzed. The expression patterns of *TaHMs* and *ZmHMs* in developing wheat grain and maize seed were investigated. Moreover, the responses of *TaHMs* to growth regulators (BR, brassinazole (BRZ), and activated charcoal (AC)) and to biotic and abiotic stresses (heat, drought, salt, insect feeding, nitrogen (N), and cadmium (Cd)) were explored, and changes in the expression profiles of *ZmHMs* after gibberellin (GA_3_) and drought treatment were also analyzed.

## Results

### Identification and characterization of *HM* genes in *T. aestivum*, *H. vulgare*, *S. bicolor*, *S. viridis*, *S. italica*, and *Z. mays*

**Fig. 1:**
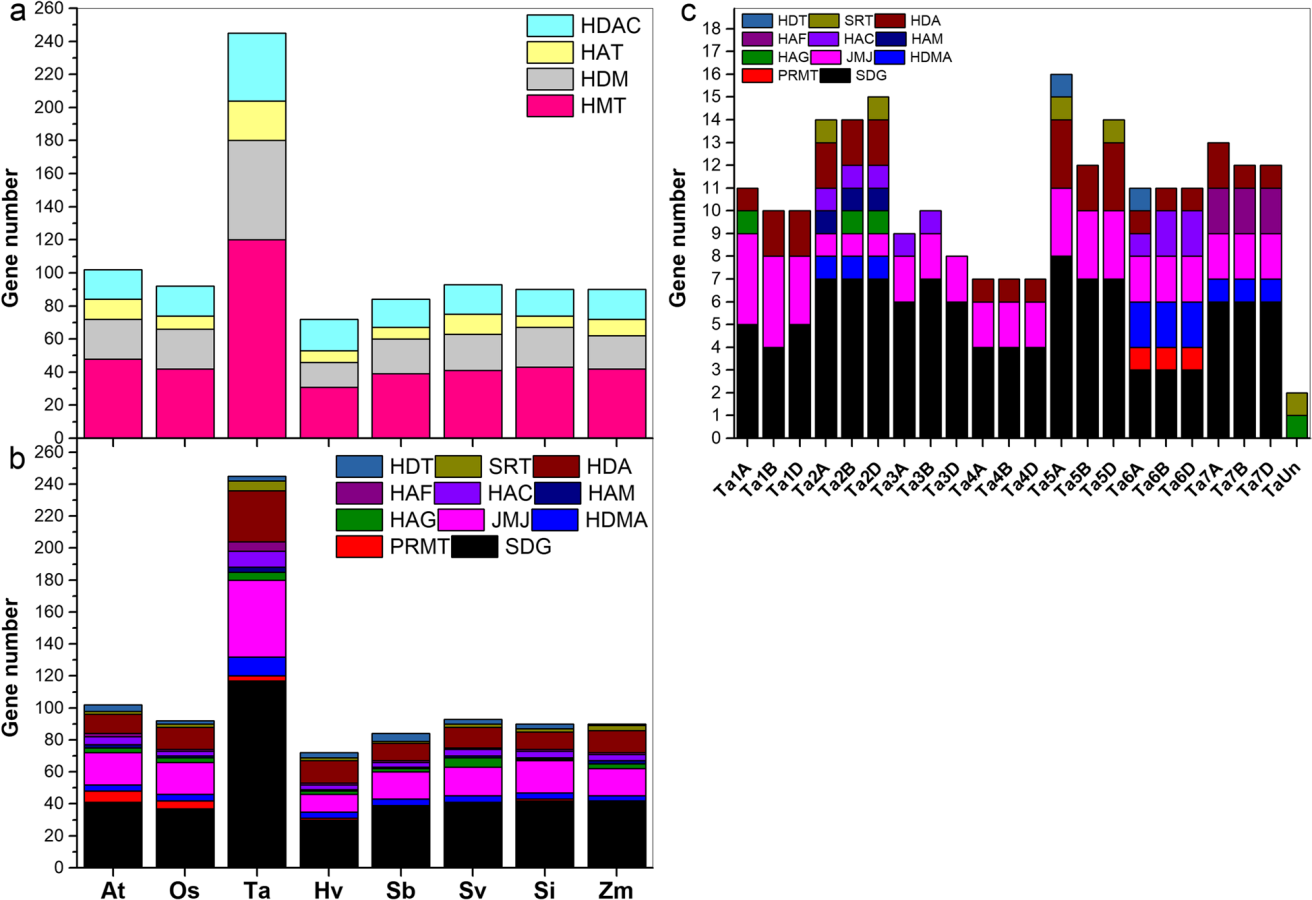
Number of *HM* genes among different species. **a** Gene number of each *HM* family in *Arabidopsis*, rice, and six Gramineae species. **b** Gene number of each *HM* subfamily in model and six Gramineae species. **c** Number of each *TaHM* subfamily on wheat chromosomes

*Arabidopsis* and rice (*Oryza sativa*) contain 102 and 92 *HMs*, including 48 and 42 *HMTs*, 24 and 24 *HDMs*, 12 and eight *HATs*, and 18 and 18 *HDACs*, respectively (Fig. 1a). In total, 245, 72, 84, 93, 90, and 90 *HMs* were identified in *T. aestivum*, *H. vulgare*, *S. bicolor*, *S. viridis*, *S. italica*, and *Z. mays*, respectively (Fig. 1a and b). The number of *HMTs*, *HDMs*, *HATs* and *HDACs* were broadly equal among the Gramineae species, except for *T. aestivum* (Fig. 1a)*.* There were 2.4- and 2.7-fold as many wheat *HMs* (*HMTs*, *HDMs*, *HATs*, and *HDACs*) than *Arabidopsis* and rice *HMs*, respectively (Fig. 1a). There were 30–117 *SDGs*, 1–7 *PRMTs*, 3–12 *HDMAs*, 11–48 *JMJs*, 1–6 *HAGs*, 1–3 *HAMs*, 3–10 *HACs*, 1–6 *HAFs*, 11–32 *HDAs*, 1–6 *SRTs*, and 1–5 *HDTs* among all species (Fig. 1b). Furthermore, there were 3–8 *T. aestivum SDGs* (*TaSDGs*), 0–1 *TaPRMT*-*TaHAG*-*TaHAM*-*TaSRT*-*TaHDT*, 0–2 *TaHDMAs*-*TaHACs*-*TaHAFs*, 1–4 *TaJMJs*, and 0–3 *TaHDAs* on chromosome 1A-7D (Fig. 1c). One *TaHAG* and one *TaSRT* were located on an unknown chromosome (Fig. 1c).

The identified Gramineae *HMs* were named based on their chromosomal location (Fig. S1). For example, wheat chromosome 5A (Ta5A) contained the most *HMs*, followed by Ta2D (Fig. 1c and S1–1). Most barley *HMs* (*HvHMs*) were found on the longest chromosome 2 (chr2H), and *HvSDG29*, *HvSDG30*, and *HvHDA14* were located on an unknown chromosome (Fig. S1–2). Sorghum *SDGs* (*SbSDGs*) were the most numerous among all *HM* genes, with 38 *SbSDGs* distributed on nine chromosomes, and chromosome 2 containing the most *SbHMs* (Fig. S1–3). Details on the Gramineae *HMs* are listed in Table S1. Their coding region (CDS) lengths ranged from 195 (*HvHDT3*) to 7008 (*AtSDG2*) bp, with the deduced polypeptides ranging from 64 to 2335 amino acids (aa).

### Conserved domain and phylogenetic analyses of *HM* genes

**Fig. 2:**
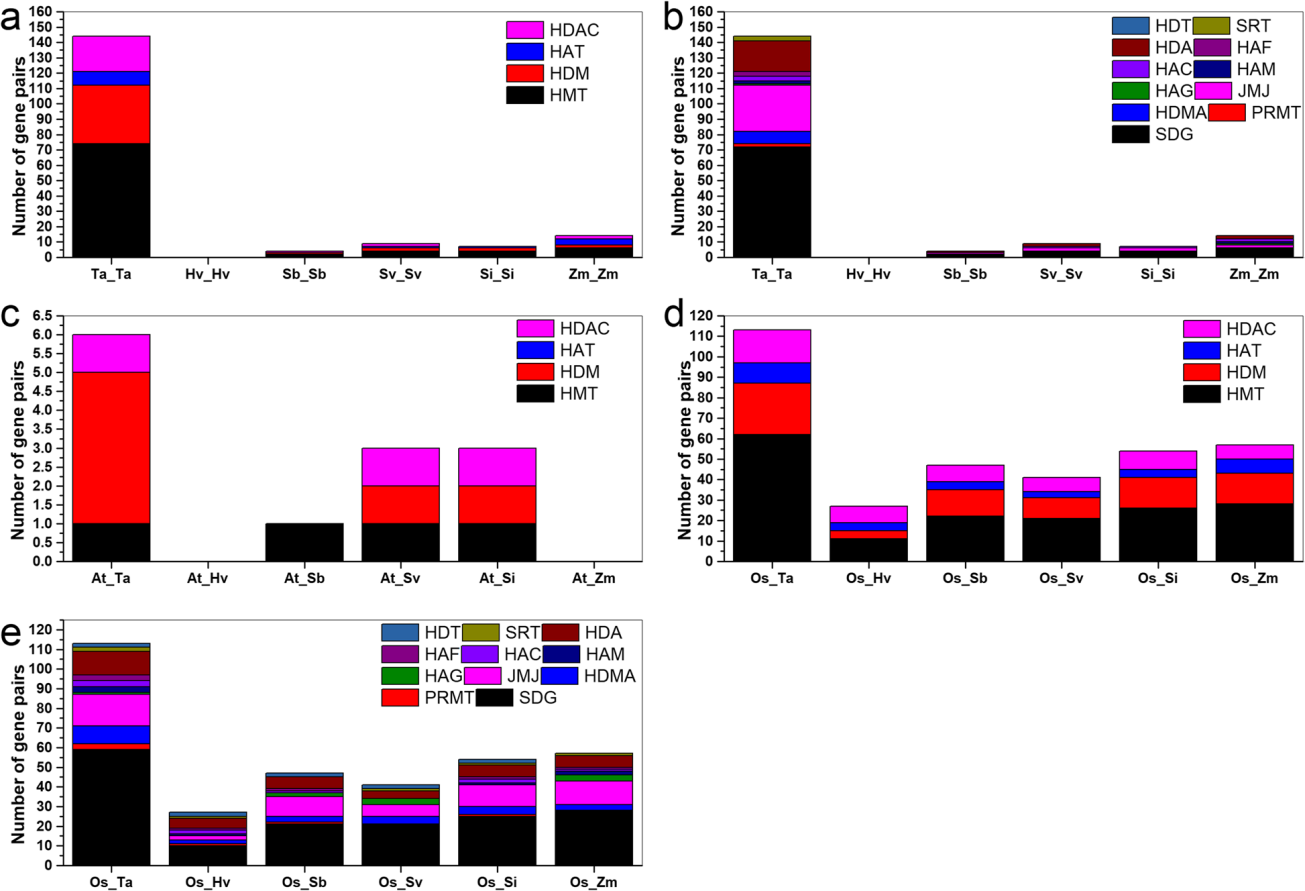
Comparison of number of gene orthologs against different genomes. **a** Number of *HM* family gene pairs among each Gramineae species. **b** Number of *HM* subfamily gene pairs among each Gramineae species. **c** Number of *HM* gene pairs between *Arabidopsis* and each Gramineae species. **d** Number of *HM* family gene pairs between rice and each Gramineae species. **e** Number of *HM* subfamily gene pairs between rice and each Gramineae species

Conserved *HM* domains were investigated, with various domains identified in the different *HMs* (Fig. S2). A total of 35 conserved motifs were identified in all *Arabidopsis* and rice *HMs* (Fig. S2–1, Fig. S2–8, Fig. S2–9, Fig. S2–10, Fig. S2–17, Fig. S2–18, Fig. S2–19, Fig. S2–20, Fig. S2–21, Fig. S2–28, and Fig. S2–29). For example, one to seven domains were found in the AtSDG and OsSDG proteins (Fig. S2–1) and six conserved motifs were identified in all AtPRMTs and OsPRMTs (Fig. S2–8). Most conserved domains identified in *T. aestivum*, *H. vulgare*, *S. bicolor*, *S. italica*, *S. viridis*, and *Z. mays* were the same as those in AtHMs and OsHMs, but several distinct domains were found in the Gramineae *HMs* (Fig. S2–2, Fig. S2–3, Fig. S2–4, Fig. S2–5, Fig. S2–6, Fig. S2–7, Fig. S2–8, Fig. S2–9, Fig. S2–10, Fig. 2–11, Fig. 2–12, Fig. 2–13, Fig. 2–14, Fig. 2–15, and Fig. 2–16). For example, 51 elements were identified in TaSDGs, most of which were the same as those found in AtSDGs and OsSDGs (Fig. S2–2). Almost all JMJ proteins included JmjC or JmjN, and specific motifs were found in the Gramineae JMJs (Fig. S2–11, Fig. S2–12, Fig. S2–13, Fig. S2–14, Fig. S2–15, and Fig. S2–16).

To clarify the evolutionary relationships among *HM* genes, unrooted phylogenetic trees were constructed (Fig. S3). All *AtHMTs* (except *AtSDG41*), *OsHMTs*, and *TaHMTs* were classed into groups A–E, which were further subdivided (Fig. S3–1). All SDGs were clustered together in classes A–D and F–H, and PRMTs were divided into group E subgroups e1 and e2 (Fig. S3–2). In Fig. S3–3, AtPRMTs, OsPRMTs, and SbPRMTs were clustered together in group A, which could be divided into subgroups a1 and a2, and all SDGs (except for AtSDG41) were identified in groups B–G. AtPRMTs, OsPRMTs, and SvRMTs were grouped in either class A or B, and the other HMTs were in classes C–H (Fig. S3–4). All *Arabidopsis*, rice, and *S. italica* SDGs were clustered together in groups B–E, with the exception of OsSDG738, SiSDG17, and SiSDG34, and PRMTs were all clustered in group A (Fig. S3–5). AtPRMTs-OsPRMTs-ZmPRMTs and AtSDGs-OsSDGs-ZmSDGs were clustered together in groups A and B–G (Fig. S3–6). In classes A, B, and D, the model and Gramineae JMJs were closely clustered, and HDMAs were divided into subclasses c1 and c2 in group C (Fig. S3–7). The evolutionary relationships among HATs were investigated (Fig. S3–8). HAGs were classified into groups B, C, and E, and HAFs, HAMs, and HACs were separately divided into groups A, D, and F. HDTs and SRTs were clustered into groups A and B, while HDAs were found in groups C and D (Fig. S3–9).

### Synteny analysis of *HM* genes

To identify expansion patterns in *HM* genes, duplicated blocks in each Gramineae genome were investigated, within which gene pairs were identified (Fig. S4). For example, 144 pairs of *TaHMs* were identified from 21 chromosomes (Fig. 2a and Fig. S4–1). Only four *SbHM* gene pairs (*SbSDG16*-*SbSDG37*, *SbSDG22*-*SbSDG26*, *SbJMJ1*-*SbJMJ10*, and *SbHDA11*-*SbHDA5*) were identified in the *S. bicolor* genome (Fig. 2a, b, and Fig. S4–2). A total of four types of *SvHM* gene pairs (i.e., four *SvSDGs*, two *SvJMJs*, one SvHAC, and two *SvHDAs*) were found (Fig. 2a, b, and Fig. S4–3). However, no *HvHM* gene pairs were identified (Fig. 2a and b).

We investigated the syntenic relationships among Gramineae and *Arabidopsis HMs* (Fig. 2c and Fig. S5). For example, one *HMT* gene pair (*AtSDG24* and *TaSDG97*), four *HDM* gene pairs (*AtJMJ13* and *TaJMJ3*, *AtJMJ13* and *TaJMJ7*, *AtJMJ13* and *TaJMJ11*, and *AtJMJ13* and *TaJMJ42*), and one *HDAC* gene pair (*AtHDA9* and *TaHDA12*) were identified between *Arabidopsis* and wheat (Fig. 2c and Fig. S5–1). Only *AtSDG24* and *SbSDG19* were found in the same *Arabidopsis* and *S. bicolor* gene pair (Fig. 2c and Fig. S5–2). No *HM* gene pairs were identified in *Arabidopsis*-barley and *Arabidopsis*-maize.

Various *HM* gene pairs were found between the rice and wheat genomes, including 62 pairs of *HMTs* (59 pairs of *SDGs* and three pairs of *PRMTs*), 25 pairs of *HDMs* (nine pairs of *HDMAs* and 16 pairs of *JMJs*), eight pairs of *HATs* (one pair of *HAGs*, three pairs of *HAMs*, three pairs of *HACs*, and three pairs of *HAFs*), and 16 pairs of *HDACs* (12 pairs of *HDAs*, two pairs of *SRTs*, and two pairs of *HDTs*) (Fig. 2d, e, and Fig. S6–1). Gene pairs between rice and other Gramineae species were also found (Fig. 2d, e, Fig. S6–2, Fig. S6–3, Fig. S6–4, Fig. S6–5, and Fig. S6–6). For example, a total of 27 pairs of *OsHMs*-*HvHMs* were identified (Fig. 2d, e, and Fig. S6–2); different *HM* gene pairs were found between *S. bicolor* and rice, including 21 pairs of *SDGs*, three pairs of *HDMAs*, 10 pairs of *JMJs*, two pairs of *HAGs*, one pair of *HACs*, *PRMTs*, and *HAFs*, six pairs of *HDAs*, and two pairs of *HDTs* (Fig. 2d, e, and Fig. S6–3).

To evaluate selection pressure during duplication of the above gene pairs, their non-synonymous (Ka), synonymous (Ks), and Ka/Ks values were calculated. Data showed that the Ka/Ks values were all less than or generally equal to 1 (Tables S2, S3, S4). However, several gene pairs, such as *SiJMJ5*-*SiJMJ19*, *AtJMJ13*-*TaJMJ3*, and *AtJMJ13*-*TaJMJ7*, shared no non-synonymous mutations based on their Ks values.

### Promoter and structural analyses of *HM* genes

*HM* genes play important roles in plant stress and defense responses [31, 32]. Various stress-related elements were identified in Gramineae *HM* genes (Fig. S7). For example, in the *TaHMT*, *TaHDM*, and *TaHDAC* genes, at least one abscisic acid-, methyl jasmonate (MeJA)-, defense-, drought-, low temperature-, or salt-related element was uncovered (Fig. S7–1, 2, and 4). Furthermore, 2–13 stress-related motifs (defense/stress, abscisic acid, and MeJA-responsiveness elements) were identified in the *HvHMT* genes. *SbSDG3*, *SbSDG13*, *SbPRMT1*, and *SbJMJ16* only contained one defense/stress, abscisic acid, or MeJA-responsiveness motif, whereas all other *SbHMs* included at least two stress-related elements (Fig. S7–6).

We next identified *HM* gene structures. In general, homologous *HM* genes, especially those in the same pair, shared similar structures, although gene lengths differed (Fig. S8). For example, most homologous *TaHMT* genes contained more than one CDS and were more than 3000 bp in length (Fig. S8–1). All *HvHMTs*, except for *HvSDG4*, shared a short non-coding sequence, and most were 2000–5000 bp in length (Fig. S8–5). Many *SbHMTs* (*SbSDGs* and *SbPRMTs*), *SbHDMs* (*SbHDMAs* and *SbJMJs*), *SbHATs* (*SbHAGs*, *SbHAMs*, *SbHACs*, and *SbHAFs*), and *SbHDACs* (*SbHDAs*, *SbSRTs*, and *SbHDTs*) consisted of short CDSs, but several genes contained one to two long CDSs (Fig. S8–9, 8–10, 8–11, and 8–12).

### Expression patterns of *TaHMs* in developing wheat grain in response to BR and AC

**Fig. 3:**
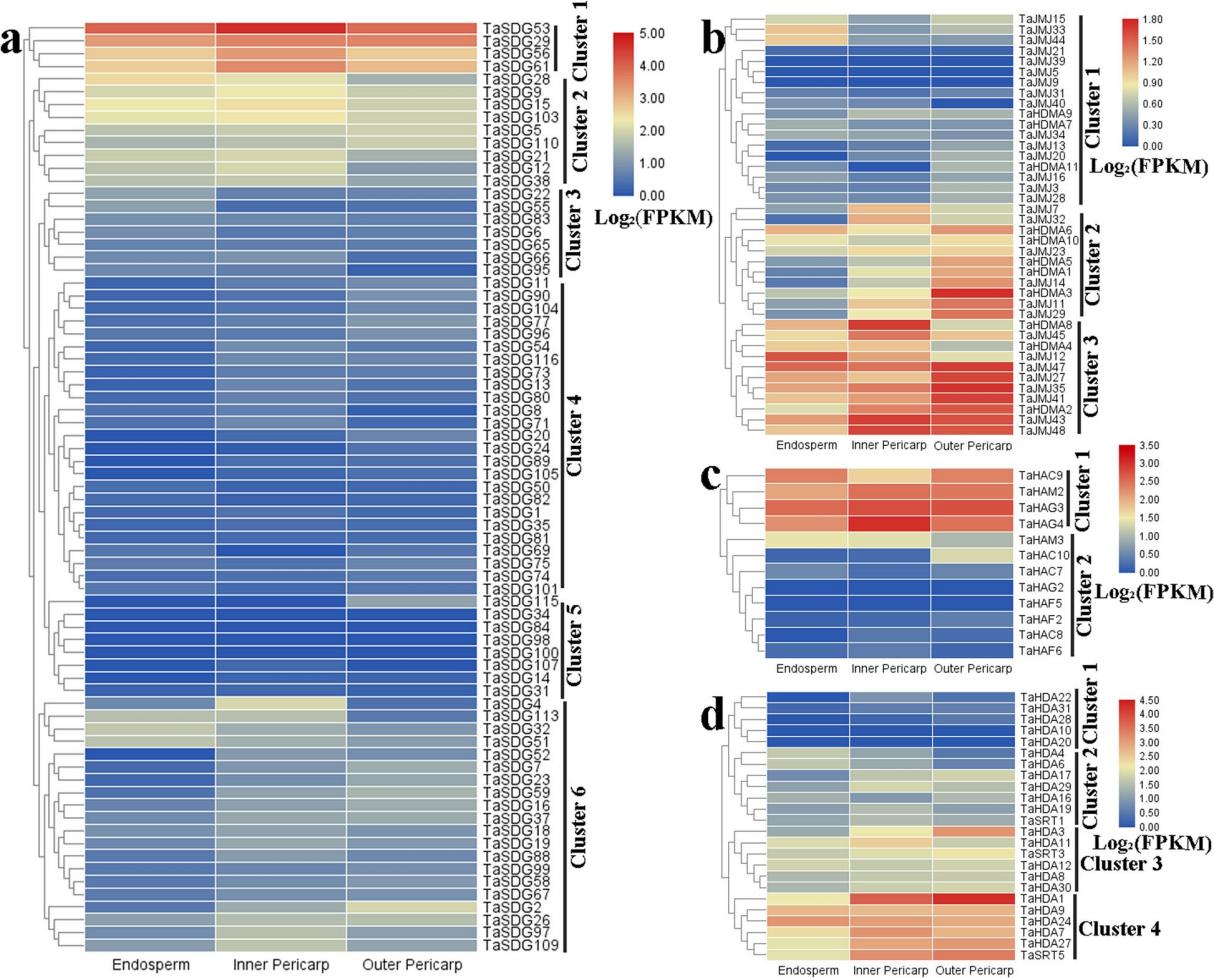
Expression profiles of *TaHMs* in various tissues of developing wheat grain. **a** *TaHMTs*: *TaSDGs*. **b** *TaHDMs*: *TaHDMAs* and *TaJMJs*. **c** *TaHATs*: *TaHAGs*, *TaHAMs*, *TaHACs* and *TaHAFs*. **d** TaHDACs: *TaHDAs*, *TaSRTs*, and *TaHDTs*. FPKM: Fragments per kilobase per million. Expression levels of other *TaHMs* not shown here were not detected in developing grain

To investigate the potential roles of *HMs* in wheat grain growth and development, we examined their expression profiles in the endosperm, inner pericarp, and outer pericarp (Fig. 3). Based on these expression patterns, *TaHMs* were divided into various clusters (Fig. 3a-d). In cluster 1, *TaSDG53*, *TaSDG29*, *TaSDG56*, and *TaSDG61* were highly expressed in all tissues, especially in the inner pericarp. In cluster 2, several *TaSDGs*, such as *TaSDG15*, *TaSDG103*, and *TaSDG21*, were also highly expressed in the inner pericarp. In cluster 3, seven *TaSDGs* were found at relatively low levels in the outer pericarp. In clusters 4 and 5, *TaSDGs* showed lower expression levels than in the other clusters. In cluster 6, most genes were highly expressed in the inner and outer pericarps (Fig. 3a). The expression levels of *TaHDMAs* and *TaJMJs* were generally low compared with other genes (Fig. 3b). *TaHATs* were classified into two classes according to their expression patterns. Genes in cluster 1 were more highly expressed in all tissues than genes in cluster 2 (Fig. 3c). In clusters 3 and 4, *TaHDAs*, *TaSRT3*, and *TaSRT5* were highly expressed in the pericarps (Fig. 3d).

**Fig. 4:**
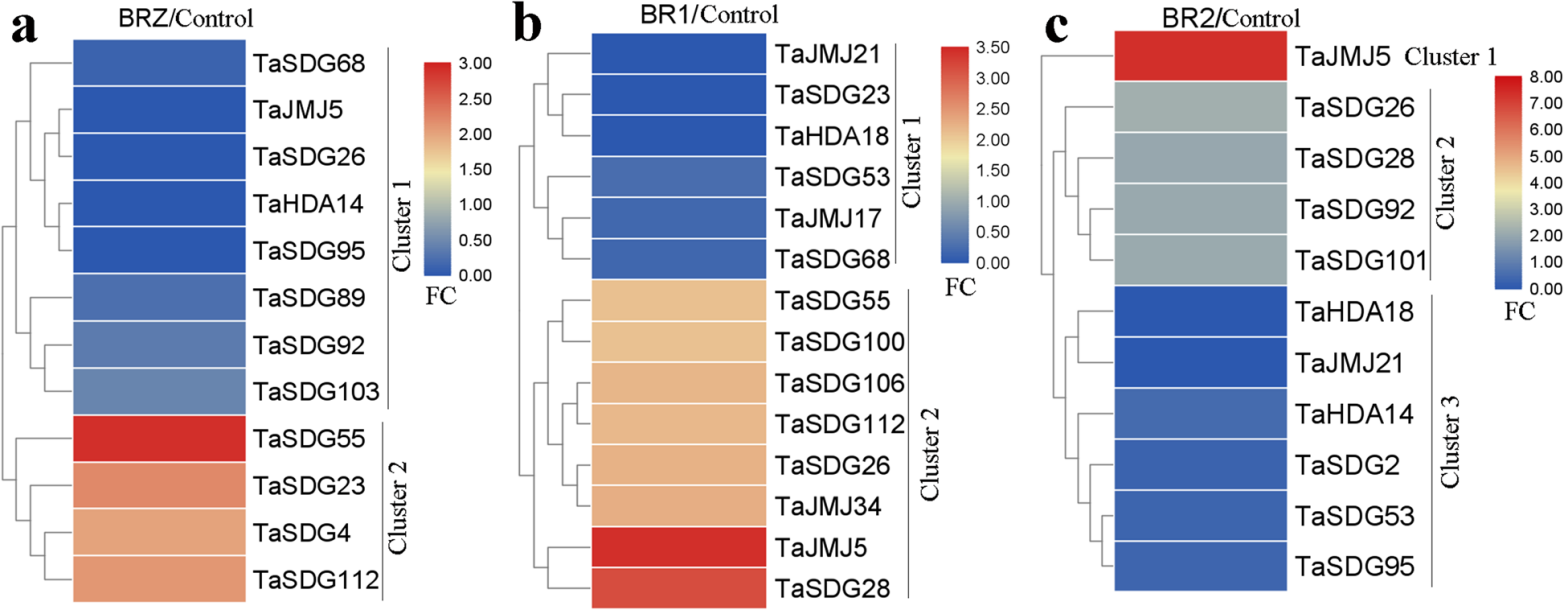
Expression analysis of *TaHMs* in response to BR and BRZ. **a** Differentially expressed *TaHMs* between BRZ-treated and control groups. **b** Differentially expressed *TaHMs* between BR1-treated and control groups. **c** Differentially expressed *TaHMs* between BR2-treated and control groups. BR1, 50 nM EpiBL; BR2, 1 mM EpiBL; BRZ, 1 mM BRZ. FC: fold-change

In *Arabidopsis*, rice, wheat, and maize, BR plays an important role in root growth, including lateral root initiation and hair formation [33–36]. BR treatment significantly increases the number of lateral roots in wheat, but inhibits root length and diameter, whereas the BR synthesis inhibitor BRZ shows the opposite roles on lateral root number and root diameter [33]. Although *HM* genes are known to regulate various developmental processes, information on their roles in regulating wheat roots is scarce. In this study, we analyzed *HM* gene expression profiles during BR- and BRZ-mediated root growth (Fig. 4). In cluster 2, *TaSDG4*, *TaSDG23*, *TaSDG55*, and *TaSDG112* showed a 2-fold increase after BRZ treatment, whereas, in cluster 1, BRZ treatment repressed *TaSDG26*, *TaSDG68*, *TaSDG89*, *TaSDG92*, *TaSDG95*, *TaSDG103*, and *TaJMJ5* expression (Fig. 4a). BR treatment increased the expression of more than 10 *TaHMs* (especially *TaJMJ5* and *TaSDG28*), while BR1 and BR2 exposure inhibited the expression of several other *TaHMs* (Fig. 4b and c). For example, *TaSDG26*, *TaSDG28*, and *TaJMJ5* were induced by BR1 and BR2 treatment; *TaSDG92* and *TaSDG101* were up-regulated by BR2 treatment; and *TaJMJ21*, *TaSDG53*, and *TaHDA18* were repressed by both BR1 and BR2 treatment.

**Fig. 5:**
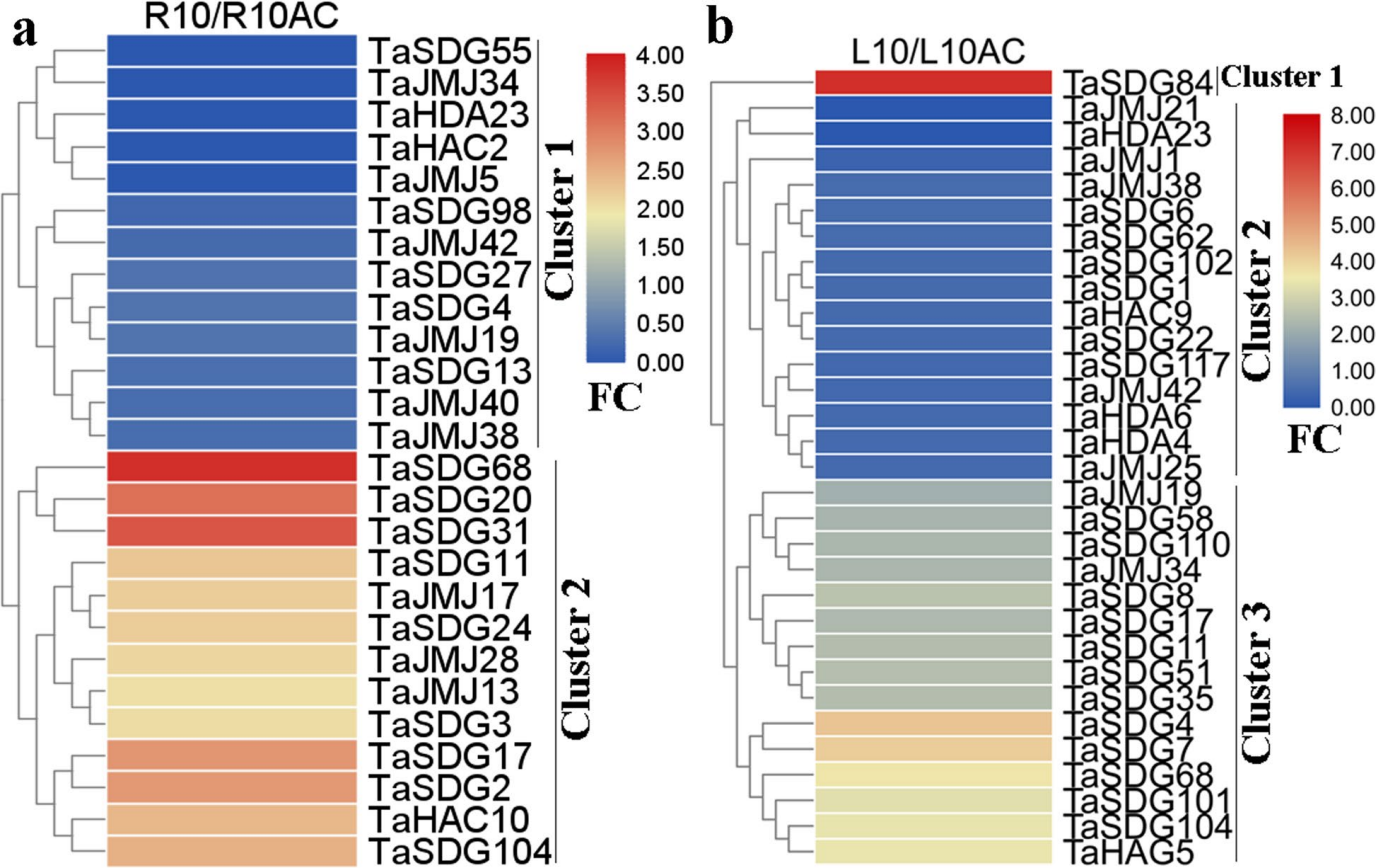
Expression pattern analysis of *TaHMs* in response to AC. **a** Differentially expressed *TaHMs* between AC (R10AC) and control (R10) groups in roots of 10-day-old seedlings. **b** Differentially expressed *TaHMs* between AC (L10) and control (L10AC) groups in leaves of 10-day-old seedlings. FC: fold-change

In plant culture, AC is widely used to promote seedling growth [37]. Notably, AC treatment promotes wheat seedling growth, accompanied by an increase in soluble protein, root activity, and total phenol and sugar content [37]. Here, we found that 26 and 31 *TaHMs* were differentially expressed in roots and leaves after AC treatment, respectively (Fig. 5), with an almost equal number down-regulated and up-regulated by AC treatment. For example, after AC treatment, *TaSDG68* and *TaSDG84* showed a 4- and 8-fold decrease in the roots and leaves, respectively; *TaSDG55* was increased in the roots; and *TaJMJ21* was up-regulated in the leaves (Fig. 5a and b).

### Responses of *TaHMs* to abiotic and biotic stresses

**Fig. 6:**
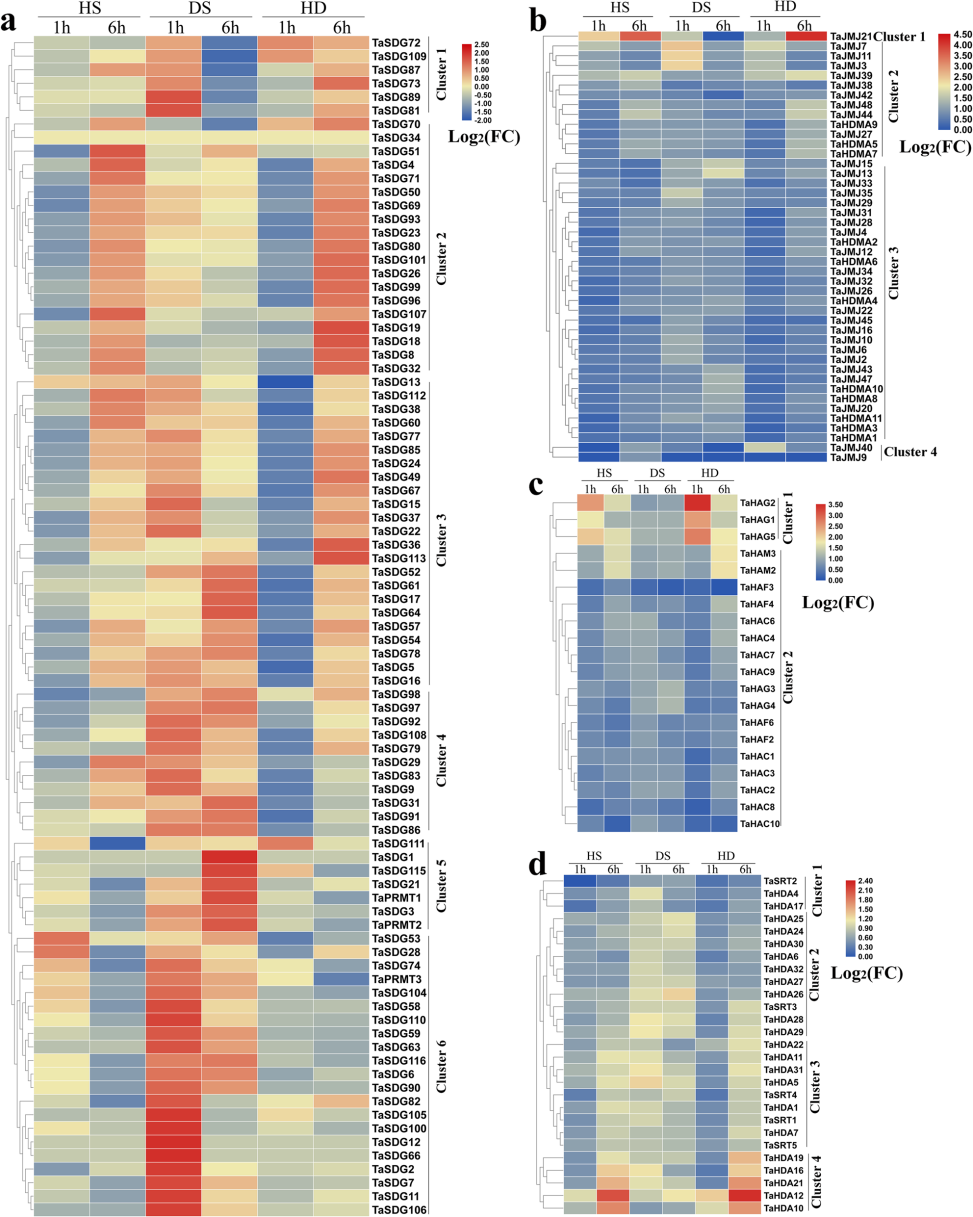
Expression pattern analysis of *TaHMs* in response to HS, DS, and HD. **a** Differentially expressed *TaHMTs* between control and HS, DS, and HD groups. **b** Differentially expressed *TaHDMs* between control and HS, DS, and HD groups. **c** Differentially expressed *TaHATs* between control and HS, DS, and HD groups. **d** Differentially expressed *TaHDACs* between control and HS, DS, and HD groups. Log_2_FC data represent level of up-regulation or down-regulation. FC: fold-change

To explore whether *TaHMs* respond to abiotic stresses, we analyzed their expression levels after heat stress (HS), drought stress (DS), and heat stress (HD) treatment using previously published RNA-seq data [38]. In total, 86 *TaHMT* genes (83 *TaSDGs* and three *TaPRMTs*) were differentially expressed at 1 or 6 h after the different treatments (Fig. 6a). These *TaHMT* genes could be divided into six clusters based on their transcription patterns. In cluster 1, almost all *TaSDGs* were induced and repressed by DS at 1 and 6 h, respectively, and were up-regulated by HD at 6 h. In cluster 2, 20 *TaSDGs* were obviously increased at 6 h after HS and HD treatment, but were decreased at 1 h, and several *TaSDGs* were clearly induced or inhibited by DS. In cluster 3, *TaSDGs* were generally induced by both HS and HD at 6 h but were suppressed at 1 h in the HS and HD groups, and increased at 1 and 6 h in the DS group. All *TaHDMs* were divided into four clusters (Fig. 6b). In cluster 1, *TaJMJ21* was highly expressed after HS and HD treatment, and increased at 1 h following DS treatment. In cluster 2, *TaJMJ7*, *TaJMJ11*, and *TaJMJ3* were generally up-regulated by DS and HD at 1 h, whereas other genes were generally up-regulated at 6 h after HS and HD treatment. *TaHATs* were clustered into two classes (Fig. 6c). In cluster 1, *TaHAG1*, *TaHAG2*, and *TaHAG5* were increased after HS and HD treatment. In cluster 2, *TaHAM2* and *TaHAM3* were obviously up-regulated at 6 h in the HS and HD groups, and other genes were induced by HS, DS, or HD at at least one time point. As shown in Fig. 6d, *TaHDA4*, *TaHDA17*, and *TaSRT2* were induced by DS in cluster 1, and *TaHDA4* was also increased in the HS group. DS treatment increased the expression levels of 10 *TaHDACs* in cluster 2, and these genes were also affected by HS and HD at several time points. Nine *TaHDACs* were distinctly induced by HS, DS, or HD treatment in cluster 3. In cluster 4, *TaHDACs* (*TaHDA10*, *TaHDA12*, *TaHDA16*, *TaHDA19*, and *TaHDA21*) were primarily expressed at 6 h in the HS and HD groups.

**Fig. 7:**
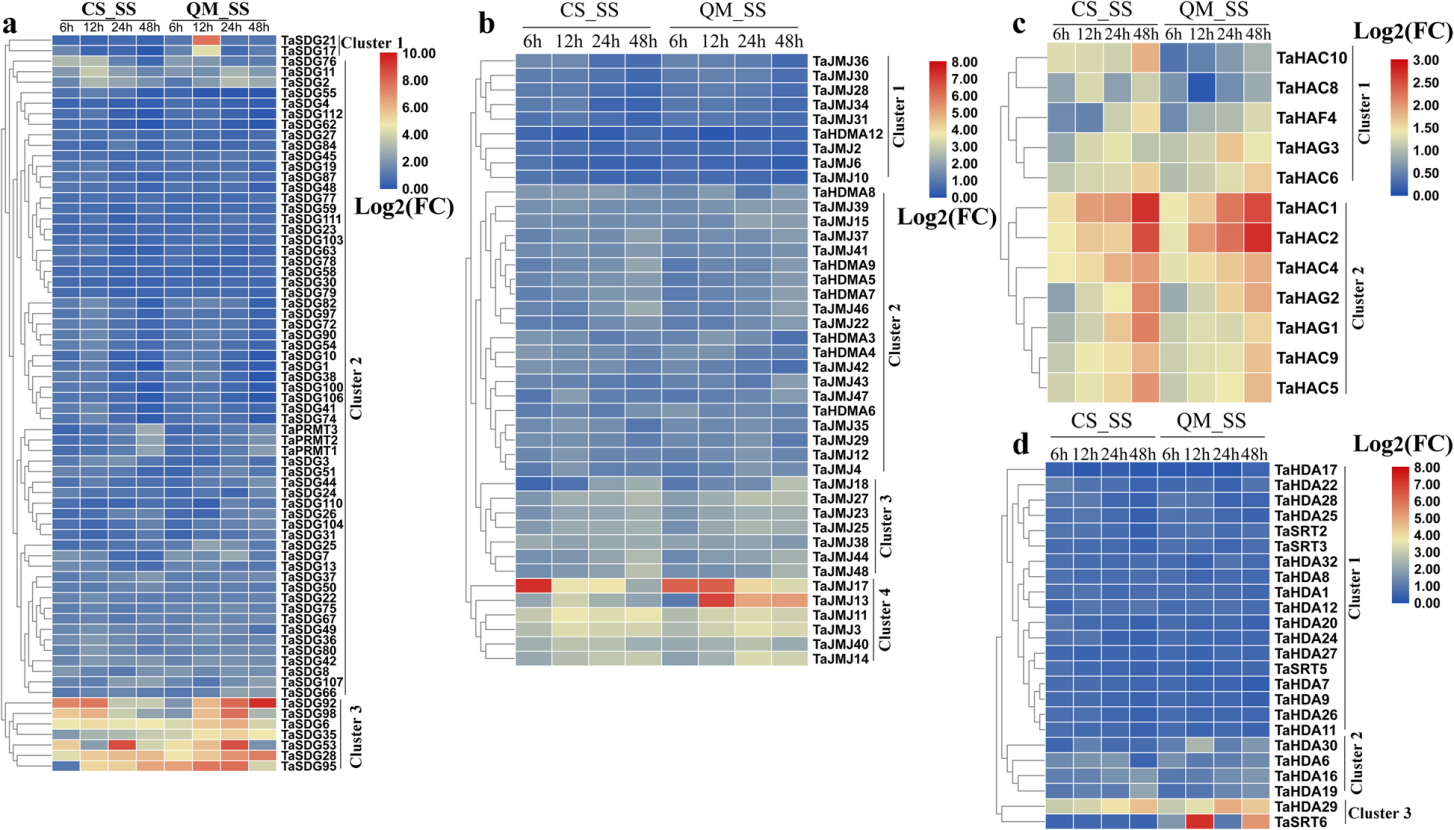
Expression pattern analysis of *TaHMs* in response to SS in two different varieties. **a** Differentially expressed *TaHMTs* between control and SS group in CS and QM. **b** Differentially expressed *TaHDMs* between control and SS group in CS and QM. **c** Differentially expressed *TaHATs* between control and SS group in CS and QM. **d** Differentially expressed *TaHDACs* between control and SS group in CS and QM. Log_2_FC data represent level of up-regulation or down-regulation. FC: fold-change

To investigate the responses of *TaHMs* to SS, we identified the expression profiles in the salt-sensitive wheat cultivar Chinese Spring (CS) and salt-insensitive wheat cultivar Qingmai 6 (QM). After SS treatment, almost all *TaHMs* were up-regulated at at least one time point (Fig. 7). The *TaHMTs* were grouped into three clusters (Fig. 7a). In cluster 1, *TaSDG17* and *TaSDG21* were significantly induced in QM at 12 h after SS treatment. In cluster 2, *TaSDGs* and *TaPRMTs* were up-regulated at several time points in both the CS and QM groups after SS treatment. Most *TaHDM* genes were up-regulated by SS from 6 to 24 h in cluster 1. Genes in cluster 2 were induced by SS treatment at most time points. The transcripts of *TaJMJ18*, *TaJMJ27*, *TaJMJ23*, *TaJMJ25*, *TaJMJ38*, *TaJMJ44*, and *TaJMJ48* increased from 12 to 48 h after SS treatment in cluster 3. In both CS and QM, six *TaJMJs* were obviously induced by SS in cluster 4 (Fig. 7b). *TaHATs* were divided into two clusters (Fig. 7c). In cluster 1, *TaHAC8* and *TaHAC10* were mainly regulated by SS in CS, and *TaHAF4*, *TaHAG3*, and *TaHAC6* were induced by SS in both the CS and QM cultivars. In cluster 2, SS treatment increased the expression levels of *TaHACs* and *TaHAGs*, especially *TaHAC1* and *TaHAC2*, at every time point (Fig. 7c). The expression levels of *TaHDACs*, especially genes in cluster 3, were also markedly increased at at least one time point after SS treatment (Fig. 7d).

Wheat pests *Sitobion avenae* and *Schizaphis graminum* can increase yield losses [39]. Compared with the non-phytotoxic aphid *S. avenae*, feeding by phytotoxic aphid *S. graminum* causes more severe damage in wheat leaves [40]. N is an essential macronutrient for plant growth and development, and low N stress can repress wheat leaf and root growth [40]. In addition, Cd can inhibit leaf photosynthesis, carbon and N metabolism, and wheat growth and yield [41]. To clarify how *TaHMs* respond to biotic, nutrition, and heavy metal stress, their expression patterns were obtained from previous transcriptome research [39–41]. In our study, *TaJMJ7* increased 3.4- and 4-fold after *S. avenae* and *S. graminum* feeding, respectively; *TaJMJ11* was induced by *S. avenae* infection; *TaJMJ40* and *TaJMJ42* increased in the *S. graminum* feeding group compared with the control; and *TaHDA17*, *TaSDG73*, *TaHDA20*, *TaSDG81*, *TaHDA22*, and *TaSDG89* were distinctly controlled following *S. graminum* feeding (Table 1). In addition, N stress suppressed *TaSDG73* and *TaHDA20* expression in the leaves, but up-regulated *TaJMJ11* and *TaJMJ3* in the roots (Table 1). Furthermore, Cd treatment induced a 2.2- to 6.4-fold increase in the expression levels of 12 *TaHMs* (e.g., *TaSDG13*, *TaJMJ28*, and *TaHDT1*) in the roots but decreased the expression of *TaSDG102* (Table 1).

**Table 1:** Expression analysis of *TaHMs* during different biotic and abiotic stresses

Gene	Sa/C	Sg/C	N/C_leaf	N/C_root	Cd/C
*TaJMJ7*	3.460546762	4.061182894	Nan	Nan	Nan
*TaJMJ11*	4.25128801	Nan	Nan	3.242752801	Nan
*TaJMJ40*	Nan	176.8521564	Nan	Nan	Nan
*TaJMJ42*	Nan	12.76303914	Nan	Nan	Nan
*TaHDA17*	Nan	0.19218931	Nan	Nan	Nan
*TaSDG73*	Nan	0.095377977	0.12244898	Nan	Nan
*TaHDA20*	Nan	0.198773867	0.114219114	Nan	Nan
*TaSDG81*	Nan	0.109196612	Nan	Nan	Nan
*TaHDA22*	Nan	0.161925264	Nan	Nan	Nan
*TaSDG89*	Nan	0.06737233	Nan	Nan	Nan
*TaJMJ3*	Nan	Nan	Nan	3.687573184	Nan
*TaSDG13*	Nan	Nan	Nan	Nan	6.470609988
*TaSDG100*	Nan	Nan	Nan	Nan	4.039565068
*TaSDG102*	Nan	Nan	Nan	Nan	0.265806191
*TaSDG66*	Nan	Nan	Nan	Nan	4.300055702
*TaSDG74*	Nan	Nan	Nan	Nan	3.367466059
*TaSDG112*	Nan	Nan	Nan	Nan	3.127601118
*TaSDG82*	Nan	Nan	Nan	Nan	3.248304396
*TaSDG106*	Nan	Nan	Nan	Nan	2.966738703
*TaSDG62*	Nan	Nan	Nan	Nan	3.971222851
*TaJMJ28*	Nan	Nan	Nan	Nan	2.453364919
*TaHDT1*	Nan	Nan	Nan	Nan	2.228164872
*TaJMJ34*	Nan	Nan	Nan	Nan	2.353792646
*TaSDG87*	Nan	Nan	Nan	Nan	2.360930064

*Sa S. avenae*, *Sg S. graminum*, *N* nitrogen stress, *Cd* cadmium stress, *C* control

### Diverse responses of *TaHMs* to growth and stress signaling

**Fig. 8:**
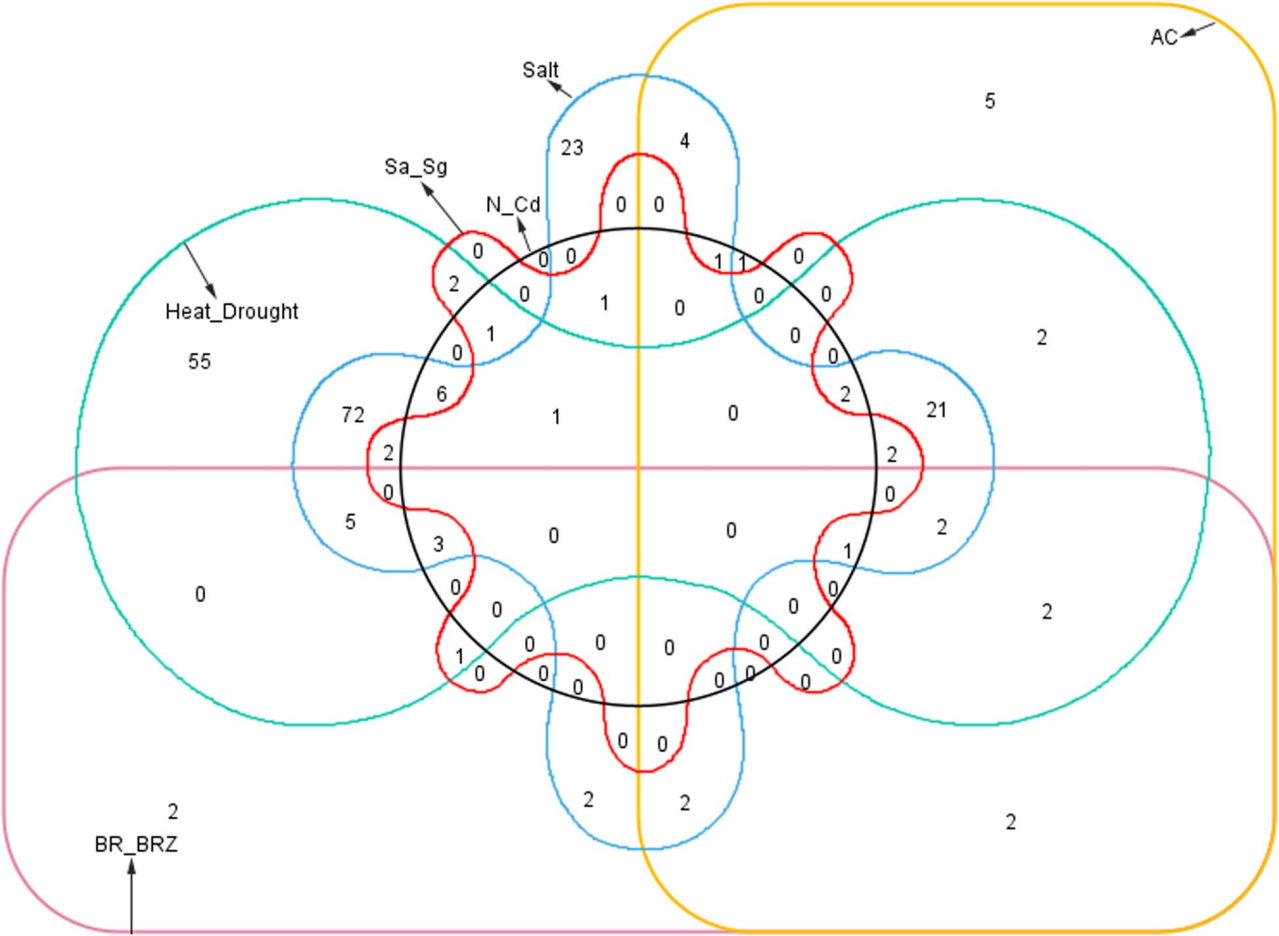
Venn analysis of DEGs under diverse growth and stress treatments

To investigate the multiple functions of *TaHMs* in wheat growth and stress adaptations, a Venn diagram was constructed with the above identified DEGs (Fig. 8). The DEGs were clustered into six sets, including the BR or BRZ (BR-BRZ) class, AC class, heat or drought (heat-drought) class, salt class, *S. avenae* or *S. graminum* (Sa-Sg) class, and N or Cd (N-Cd) class (Fig. 8 and Table S5). Some *TaHMs* were simultaneously respond to various signals, while several ones were only regulated by single clue. For example, two *TaHMs* (*TaSDG68* and *TaJMJ5*) were concurrently in response to BR-BRZ and AC; the expression patterns of *TaSDG95* and *TaSDG103* were altered by both BR-BRZ and salt treatment; and 72 *TaHMs* simultaneously responded to heat, drought, and salt stress. We found that *TaSDG13* and *TaJMJ28* were common DEGs after AC, heat-drought, salt, and N-Cd treatment. *TaJMJ34* was commonly induced or repressed by BR-BRZ, AC, heat-drought, salt, and N-Cd treatment. In total, 55, 23, five, and two *TaHMs* responded to heat-drought, salt, AC, and BR-BRZ, stress, respectively (Fig. 8 and Table S5).

### Expression analysis of *ZmHMs* in developing seed and response to GA treatment

**Fig. 9:**
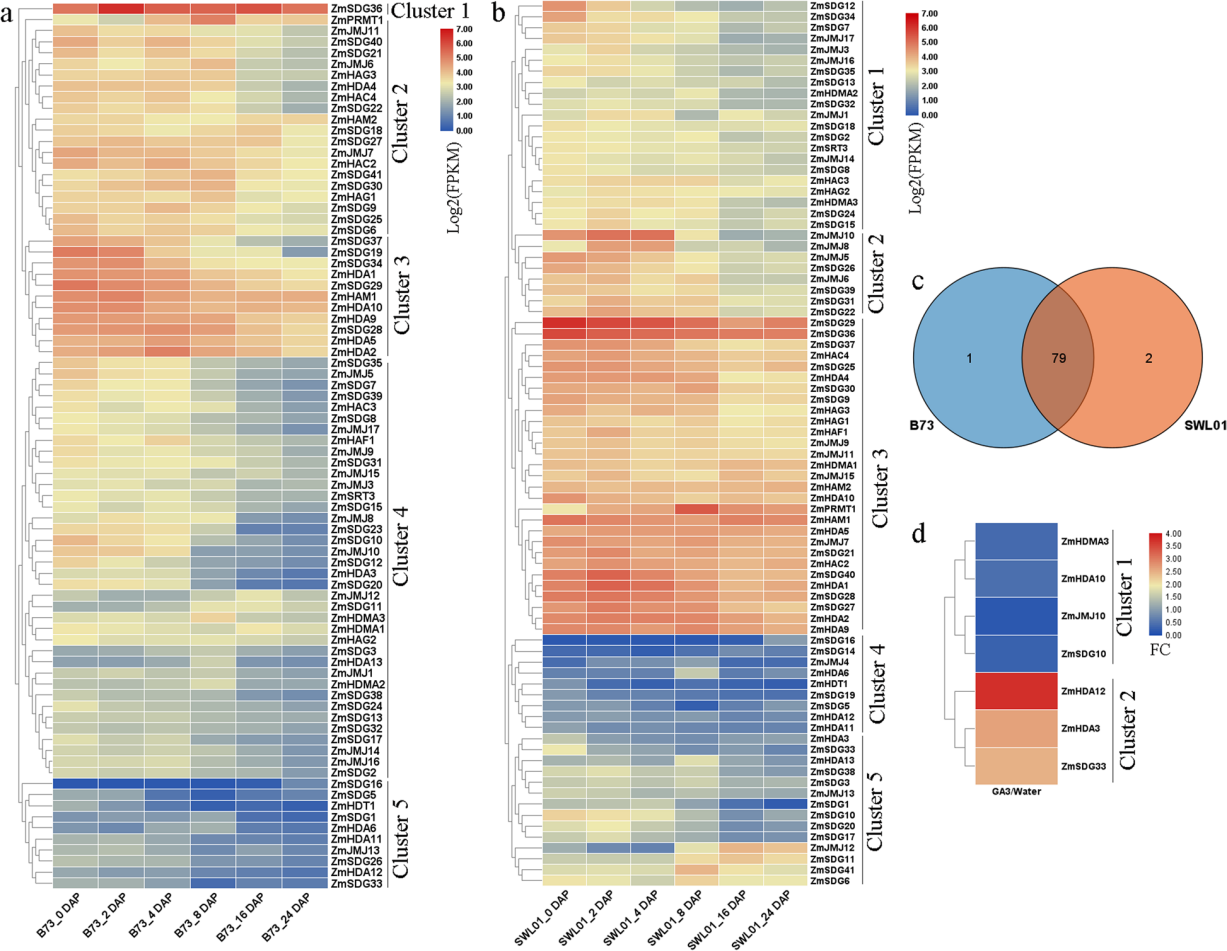
Expression profiles of *ZmHMs* in developing seeds and in response to GA signaling. **a** Expression profiles of *ZmHMs* in developing B73 seeds. **b** Expression profiles of *ZmHMs* in developing SWL01 seeds. **c** Venn analysis of genes expressed in B73 and SWL01 seeds. **d** Differentially expressed *TaHMs* between GA-treated and control groups. FPKM: fragments per kilobase per million. FC: fold-change

To investigate the functions of *ZmHMs* in maize growth and development, we analyzed the expression profiles of *ZmHMs* in different seed growth stages of B73 and SWL01 cultivars (Fig. 9a and b). The SWL01 cultivar is a mutant of B73 and shows higher viscosity [42]. From 0 to 24 days (d) after pollination (DAP), 80 *ZmHM* genes were clustered into five classes (Fig. 9a). During the whole experimental period (especially at 2 DAP), *ZmSDG36* in cluster 1 showed higher expression than genes in other classes. In cluster 2, *ZmHMs* showed higher expression at the early stages (from 0 to 8 DAP) than during the later periods (from 16 to 24 DAP). In cluster 3, 11 *ZmHMs* were highly expressed at all stages, especially from 0 to 4 DAP. There were 81 *ZmHM* genes detected during SWL01 seed development (Fig. 9b). Like genes in B73, these *ZmHMs* were distributed into five clusters in SWL01. For example, *ZmHMs* in clusters 1 and 2 (especially cluster 2) were mainly expressed at 0, 2, and 4 DAP. *ZmHMs*, such as *ZmSDG29*, *ZmSDG36*, *ZmSDG40*, and *ZmHDA1*, showed higher expression levels in cluster 3 than genes in other clusters. A total of 79 *ZmHMs* were commonly expressed in both B73 and SWL01 seeds, but most showed different expression patterns between the two cultivars. For example, *ZmSDG41* expression was higher in B73 than in SWL01; *ZmHAF1* gradually decreased over time in B73 but showed almost no change in SWL01 (Fig. 9c). GA_3_ application significantly promoted leaf sheath growth of *D11* [43]. Seven *ZmHM* genes were differentially expressed between the GA and control groups (Fig. 9d). In cluster 1, *ZmHDMA3*, *ZmHDA10*, *ZmJMJ10*, and *ZmSDG10* were down-regulated by GA, whereas *ZmHDA12*, *ZmHDA3*, and *ZmSDG33* were up-regulated.

### Expression analysis of *ZmHMs* in response to drought stress

To identify the potential roles of *ZmHMs* in drought adaptation, their expression patterns were analyzed in drought-tolerant cultivars (ND476 and H082183), drought-sensitive cultivars (ZX978 and Lv28), and C7–2 (Table 2). In total, 10 *ZmHMs* were identified as DEGs in response to drought stress. After drought treatment, the transcription level of *ZmJMJ2* showed a 6-fold increase in ND476 compared with ZX978, whereas *ZmHDA11* was repressed in ND476. *ZmSDG5*, *ZmJMJ4*, and *ZmSDG24* were induced by drought treatment in C7–2, but *ZmSDG33* and *ZmJMJ17* were controlled. In Lv28 and H082183, *ZmJMJ5* was up-regulated under both moderate and severe drought treatment. The expression level of *ZmSDG1* increased in H082183 after moderate drought treatment, whereas *ZmHDA2* was markedly down-regulated after severe drought treatment.

**Table 2:** Expression analysis of *ZmHMs* during different drought stresses

Gene_id	TD/SD	CD/CC	LMD/LMC	LSD/LSC	HMD/HMC	HSD/HSD
*ZmJMJ2*	6.086	Nan	Nan	Nan	Nan	Nan
*ZmHDA11*	0.249	Nan	Nan	Nan	Nan	Nan
*ZmSDG5*	Nan	9.388824371	Nan	Nan	Nan	Nan
*ZmJMJ4*	Nan	3.074936123	Nan	Nan	Nan	Nan
*ZmSDG24*	Nan	2.363578856	Nan	Nan	Nan	Nan
*ZmSDG33*	Nan	0.457540122	Nan	Nan	Nan	Nan
*ZmJMJ17*	Nan	0.390122323	Nan	Nan	Nan	Nan
*ZmJMJ5*	Nan	Nan	Up	Nan	Nan	Up
*ZmSDG1*	Nan	Nan	Nan	Nan	Up	Nan
*ZmHDA2*	Nan	Nan	Nan	Nan	Nan	Down

*TD* tolerant cultivar ND476 drought treatment, *SD* sensitive cultivar ZX978 drought treatment, *CD* C7–2 drought treatment, *CC* C7–2 control, *LMD* Lv28 moderate drought treatment, *LMC* Lv28 control, *LSD* Lv28 severe drought treatment, *LSC* Lv28 control, *HMD* H082183 moderate drought treatment, *HMC* H082183 control, *HSD* H082183 severe drought treatment, *HSC* H082183 control

## Discussion

Although *HM* genes are known to play essential roles in plant growth and biotic and abiotic stress in model plants [8, 18, 20], little information has been reported for Gramineae species. Here, we systematically characterized *TaHMs*, *HvHMs*, *SbHMs*, *SvHMs*, *SiHMs*, and *ZmHMs*, including information on their gene location, conserved domains, gene phylogeny, gene expansion, synteny, promoter *cis*-elements, and gene structure. Moreover, we analyzed their expression levels in wheat and maize in regard to growth and stress adaptations. These findings will provide a basis for further functional analyses of *HM* genes.

### Comparison of *HM* genes between Gramineae and model plants

Based on previous research, there are 48 *AtHMTs*, 24 *AtHDMs*, 12 *AtHATs*, and 18 *AtHDACs* in *Arabidopsis* [17, 19] and 92 *OsHMs*, including 42 *OsHMTs*, 24 *OsHDMs*, eight *OsHATs*, and 18 *OsHDACs* in *O. sativa* [44]. In the six Gramineae plants, we identified 245 *TaHMs* (120 *TaHMTs*, 60 *TaHDMs*, 24 *TaHATs*, and 41 *TaHDACs*), 72 *HvHMs* (31 *HvHMTs*, 15 *HvHDMs*, seven *HvHATs*, and 19 *HvHDACs*), 84 *SbHMs* (39 *SbHMTs*, 21 *SbHDMs*, seven *SbHATs*, 17 *SbHDACs*), 93 *SvHMs* (41 *SvHMTs*, 22 *SvHDMs*, 12 *HvHATs*, and 18 *SvHDACs*), 90 *SiHMs* (43 *SiHMTs*, 24 *SiHDMs*, seven *SiHATs*, and 16 *SiHDACs*), and 90 *ZmHMs* (42 *ZmHMTs*, 20 *ZmHDMs*, 10 *ZmHATs*, and 18 *ZmHDACs*) (Fig. 1). In terms of gene number, we found 2.4- and 2.6-fold greater number of *TaHMs* than *AtHMs* and *OsHMs*, respectively. *TaSDGs*, *TaHDMAs*, *TaJMJs*, *TaHAGs*, *TaHAMs*, *TaHAC*s, *TaHAFs*, *TaHDAs*, and *TaSRTs* were increased 1.5–3-fold. However, the number *HM* genes in other species varied slightly compared with those in the model plants (Fig. 1a and b). In wheat, a total of 144 gene pairs were identified in 10 kinds of *HM* genes, but there were 4–14 *HM* gene pairs among *S. bicolor*, *S. viridis*, *S. italica*, and *Z. mays*, and no gene duplication in *H. vulgare*. Genome duplication occurs during species evolution [45], and the wheat genome contains three homologous subgenomes [22]. Therefore, the expansions in wheat *HM* genes may be associated with gene and genome duplications during evolution.

In general, Gramineae, *Arabidopsis*, and rice *HM* genes shared similar domains (Fig. S2), although there were several exceptions. For example, *TaSDGs*, *HvSDGs*, *ZmSDGs*, *TaJMJs*, and *SbJMJs* contained 15, two, 10, 14, and eight special motifs, respectively (Fig. S2–2, 2–3, 2–7, 2–11, and 2–13). As new functions can be predicted from unique domains, greater attention should be paid to those genes sharing special elements in the future. According to phylogenetic analysis, each type of *HM* gene was clustered together (Fig. S3), although there were exceptions. For example, *AtSDG41*, *HvSDG4*, *SiSDG17*, *SiSDG34*, and *OsSDG738* shared a close relationship with *PRMTs* other than *SDGs* (Fig. S3–1, 3–2, and 3–5). This may be due to their incompletely matching protein sequences.

To better understand Gramineae *HMs*, duplicated blocks between model plants and Gramineae were determined. In this study, 13 orthologous genes were identified between *Arabidopsis* and the six Gramineae species (Fig. S5 and Table S3)), and 389 rice-Gramineae gene pairs were found (Fig. S6 and Table S4), indicating that these gene pairs shared common ancestors. Gene pairs showed considerable differences between *Arabidopsis*-Gramineae and rice-Gramineae in terms of number, which may be due to the diversity in evolutionary history between monocotyledons and dicotyledons. Several *AtHMs* and *OsHMs* are involved in plant growth and stress responses [9, 10, 12, 15–17, 20, 21, 46–48]. Although many unknown Gramineae *HMs* could be inferred from the orthologous genes of model plants, these predictions must be confirmed in future experiments. Gene evolution mode can be determined through Ka/Ks values. Here, the Ka/Ks ratios of all gene pairs were less than 1, indicating purifying selection [49].

### Potential functions of *TaHMs* and *ZmHMs* in plant growth and stress responses

Like transcription factors, *HMs* are important regulators of many biological processes, including plant growth and development [1–3]. We proposed that *TaHMs* and *ZmHMs* share similar roles with known *HMs*. Candidate *TaHMs* involved in wheat grain development and *ZmHMs* involved in maize seed development were characterized in this study. Expression patterns showed that almost all *TaHMs* (especially *TaSDGs* in cluster 1 (Fig. 3a), *TaHDMs* in cluster 3 (Fig. 3b), *TaHATs* in cluster 1 (Fig. 3c) and *TaHDACs* in cluster 4 (Fig. 3d)) were expressed in developing wheat grains, and many genes were highly expressed in specific grain tissue layers (Fig. 3). About 80% *ZmHMs* showed different expression patterns in developing maize seeds (Fig. 9a and b). Several *ZmHM* genes specifically expressed in B73 (*ZmSDG23*) or SWL01 (*ZmSDG14* and *ZmJMJ4*) were found (Fig. 9c). In addition, several commonly expressed *ZmHMs* between B73 and SWL01 were found but showed varied expression patterns (Fig. 9c). Moreover, seed-specific motifs of *ZmHMs* were identified. These findings suggest that *TaHM* genes affect grain growth and development, most *ZmHM* genes play roles in wax and regular maize seed development, and several *ZmHM* genes specifically participate in regulating seed viscosity.

BR is an essential plant hormone and stimulates wheat root hair formation and lateral root initiation [33]. However, responses of *TaHMs* to BR and BRZ are not known. In this study, four *TaSDGs* were induced by BRZ, but six *TaSDGs* as well as *TaJMJ5* and *TaHDA14* were repressed (Fig. 4a). In addition, BR respectively increased or decreased the expression of 11 *TaHMs* (Fig. 4b). We also found that GA treatment stimulated leaf sheath elongation of maize seedlings and altered the expression of seven *ZmHMs* (Fig. 9d). The above results indicate that these *TaSDGs* and *ZmSDGs* are likely involved in BR-mediated root growth and GA-mediated leaf development. AC is a positive growth regulator in wheat culture [37]. However, the relationship between *TaHMs* and AC is unclear. Here, 26 *TaHMs* were differentially expressed between the control and AC-treated roots, with about half repressed or induced by AC, respectively (Fig. 5a). In leaves, 16 *TaHMs* were regulated by AC, with 15 found to be highly expressed (Fig. 5b). Thus, these up- and down-regulated *TaHMs* are speculated to play important roles in AC-promoted wheat seedling growth.

In addition to their important functions in growth, *HM* genes also play essential roles in plant defenses [9, 17, 21, 46]. Here, *TaHM*-mediated stress responses were explored (Figs. 6-7 and Table 1). In total, 86 *TaHMTs* were differentially expressed after HS, DS, or HD treatment (Fig. 6a), and 45 *TaHDMs*, 20 *TaHATs*, and 27 *TaHDACs* were induced by stress treatment (Fig. 6b-d). In response to SS, almost all *TaHMs* were increased, especially *TaSDGs* in cluster 3 (Fig. 7a), *TaJMJs* in cluster 4 (Fig. 7b), *TaHATs* in cluster 2 (Fig. 7c), and *TaHDACs* in cluster 3 (Fig. 7d). The expression patterns of 10 *TaHMs*, including *TaSDG73*, *TaSDG81*, *TaSDG89*, *TaJMJ7*, *TaJMJ11*, *TaJMJ40*, *TaJMJ42*, *TaHDA17*, *TaHDA20*, and *TaHDA22*, were affected by *S. avenae* or *S. graminum* feeding (Table 1). Furthermore, N stress regulated the expression of four *TaHMs* (*TaSDG73*, *TaJMJ3*, *TaJMJ11*, and *TaHDA20*) (Table 1). Transcriptions of 13 *TaHMs* were influenced by Cd treatment, with most found to be increased (Table 1). Several *ZmHMs* were up-regulated or down-regulated by drought treatment (Table 2). A number of stress-related elements were identified in *TaHMs* and *ZmHMs*, which may partly explain their responses to stress. The above findings suggest the occurrence of methylation when wheat and maize experience biotic or abiotic stresses.

The multiple functions of *TaHMs* are discussed in Fig. 8 and Table S5. In total, 85 *TaHMs* were simultaneously regulated by two signals; 25 *TaHMs* were simultaneously regulated by three treatments; nine *TaHMs* were up-regulated or down-regulated by four signals; and one wheat gene was simultaneously regulated by five treatments. The diverse functions of these *TaHMs* indicate that they are essential for wheat growth and stress adaptations, and thus warrant further study. Moreover, all *ZmHMs*, except for *ZmJMJ2* and *ZmJMJ4*, that responded to drought stress were also identified in developing seeds, indicating their roles in maize growth and stress adaptations.

## Conclusions

*TaHMs*, *HvHMs*, *SbHMs*, *SvHMs*, *SiHMs*, and *ZmHMs* were systematically explored in our study to clarify their chromosome locations, protein structures, gene duplications, promoters, and gene structures. Phylogenetic and synteny comparisons between model plant and Gramineae *HMs* were performed and the potential roles of Gramineae *HMs* were posited through their known homologs. The unique characteristics of the *HM* genes were investigated based on their domains and expansions. Specific domains were identified in several Gramineae species, e.g., SDGs, PRMTs, JMJs, HDAs, and HDTs, which may exhibit unique functions. The expansion patterns of Gramineae *HMs* were analyzed to elucidate differences in gene number and function among Gramineae species. Using previously published RNA-seq data, we also investigated the potential roles of *TaHMs* in developing grain, as well as BR-mediated root growth and AC-regulated seedling development and explored the functions of *ZmHMs* in seed development and GA-mediated leaf growth. Candidate wheat HMs involved in high temperature, drought, salt, insect feeding, nutrition and heavy metal stress were analyzed. In addition, the ZmHMs involved in drought response were examined, and their responses to the above-mentioned stresses were inferred through promoter analysis. In summary, based on bioinformatics analysis, we predicted the functions of the Gramineae HMs, with the potential functions of wheat and maize in growth and stress adaptations verified based on expression profile analysis. The results of this study will lay the foundation for future research.

## Methods

### Identification and naming of *HMs*

The HMM files of each type of *HM* gene were downloaded from the Pfam database (http://pfam.sanger.ac.uk/) according to published IDs (HMTs: SDG-PF00856, PRMT-PF05185; HDMs:

HDMA-PF04433, JMJ-PF02373; HATs: HAG-PF00583, HAM-PF01853, HAC-PF08214, HAF-PF09247; HDACs: HDA-PF00850, SRT-PF02146) [6]. Using HMMER v3.0, the *T. aestivum*, *H. vulgare*, *S. bicolor*, *S. viridis*, *S. italica*, and *Z. mays* genomes were searched with HMM files [50]. No available HDTs was found in the Pfam database, but we obtained *Arabidopsis* and rice HDT proteins [7] to blast the above Gramineae protein databases using the ‘Blast Several Sequence to a Big Database’ tool in TBtools [51]. Phylogenetic analyses were used to confirm putative Gramineae HM protein sequences. Based on their chromosomal location, the genes were named as described in previous studies [51–56].

### Protein domain composition, gene structure, promotor *cis*-acting elements, phylogenetic tree, orthologous genes, and heatmap analyses

Using protein sequences, protein domain files were generated from the Batch CD-Search database (https://www.ncbi.nlm.nih.gov/Structure/bwrpsb/bwrpsb.cgi). Visualization of conserved motifs was completed with ‘Visualize NCBI CDD Domain Pattern’ in TBtools [51]. ‘Gene Structure View (Advanced)’ in TBtools [51] was employed to investigate the *HM* gene structures. Promoter analysis was performed in the PlantCARE database (http://bioinformatics.psb.ugent.be/webtools/plantcare/html/) and visualized using ‘Simple Biosequence Viewer’ in TBtools [51]. MEGA X was used to construct a phylogenetic tree with the maximum-likelihood method, and protein sequence alignment was completed using the ClustalW method [57]. Synteny blocks were obtained from genome sequences and general feature format v3 (gff3). The ‘Fasta stats, Table Row Extract Or Filter, File Merge For Mcscanx, File Transformat For Microsynteny Viewer And Advanced Circos’ tools in TBtools [51] were used to visualize syntenic relationships among homologous *HM* genes. Using ‘Simple Ka/Ks Calculator (NG)’ in TBtools, the non-synonymous (Ka) and synonymous (Ks) nucleotide substitutions of orthologous gene pairs were calculated with coding sequences [51]. Heat maps were generated with the ‘HeatMap’ tool in TBtools [51].

### Plant material and treatment

All plant materials mentioned here were used in previous studies, which provide the original sources and formal identification of plant materials.

#### Seed development

Immature wheat grains were dissected into endosperm and inner and outer seed pericarp tissues at 12 d post-anthesis and sampled for library construction and RNA sequencing (RNA-seq). RNA-seq expression data [fragments per kilobase per million (FPKM)] in the above tissues were uploaded to the WheatExp database (https://wheat.pw.usda.gov/WheatExp/about.html) [58]. For regular field-grown maize (B73), seeds were sampled at each growth phase. The waxy maize inbred line (SWL01) was grown and sampled in the same way. We downloaded transcriptome data from a previous study [42].

#### BR treatment

Three-day-old wheat seedlings were treated with 50 nM epibrassinolide (EpiBL, Sigma, USA) (BR1-treated group), 1 mM EpiBL (BR2-treated group), or 1 mM BRZ (BR synthesis inhibitor, Sigma) (BRZ-treated group). After 12 d of treatment, wheat roots were sampled and used for RNA-seq analysis [33]. Differentially expressed genes (DEGs) were provided by the corresponding author of a previously published study [33].

#### GA_3_ treatment

Dwarf *D11* is a GA-sensitive maize mutant. Seedlings in the control and GA-treated groups were treated daily with distilled water or10^− 4^ M GA_3_ (Sigma), respectively. The second leaf sheaths of *D11* were collected at the three fully-expanded-leaves (V3) stage for RNA-seq, as described previously [43]. The RNA-seq data were obtained from a previously published study [43].

#### AC treatment

After sterilization, immature wheat (*T. aestivum*) embryos were used to investigate the effects of AC (a widely used growth regulator in plant tissue culture) on wheat seedling growth in medium. The roots and leaves were used for RNA isolation and library construction, respectively. The DEGs were obtained from the supplementary data of a previously published paper [37].

#### Heat, drought, and salt treatment

In the control group, seven-day-old TAM 107 seedlings were grown in hydroponic solution under 16 h day (22 °C) and 8 h night (18 °C) conditions. Seedlings were treated with 40 °C in the HS group, with 20% (m/V) PEG-6000 in the DS group, and with combined heat (40 °C) and drought stress (40 °C and 20% PEG-6000) in the HD group [38]. Seedling leaves were sampled for RNA-seq after 1 and 6 h of stress treatment. DEG analysis was performed by Liu et al. (2015), and DEGs were downloaded from the WheatExp database (https://wheat.pw.usda.gov/WheatExp/about.html) [38].

In terms of drought treatment for maize, three published studies were cited [59–61]. Firstly, drought-sensitive ZX978 and drought-tolerant ND476 cultivars were used in early study. Maize seedlings planted in soil with 70–80% and 15–20% water content were set as the control and drought-treated groups, respectively. After 12 d of treatment, flag leaves of ND476 and ZX978 under both control and drought conditions were sampled for RNA-seq [60]. Secondly, maize ChangC7–2 (C7–2) seedlings were used for identifying drought-tolerant mechanisms. After 7 d of drought treatment, the expanded third leaves of seven-day-old C7–2 seedlings were sampled for RNA-seq analysis. The DEGs detected between the control and drought-treated groups were obtained from the additional files of a previously published study [59]. Thirdly, two maize inbred lines (drought-sensitive inbred line Lv28 and drought-tolerant inbred line H082183) were used for maize drought tolerance analysis. In the control group, Lv28 and H082183 seedlings were well-watered. In the moderate drought (MD) and severe drought (SD) treatment groups, maize seedlings were subjected to 27 and 46 d of drought treatment, respectively. The roots of Lv28 and H082183 were sampled for RNA-seq, as described in Zhang et al. (2017) [61]. DEGs between the drought (moderate and severe drought) and control groups were obtained from Zhang et al. (2017) [61].

Salt-tolerant cultivar QM and salt-sensitive cultivar CS were used to detect responses of wheat to salt [62]. The growth conditions of the QM and CS seedlings were the same as for TAM 107. For salt treatment, 150 mmol L^− 1^ NaCl was added to solution. The roots of QM and CS were collected at 6, 12, 24, and 48 h after salt treatment [62]. Salt-related DEGs were obtained from previous study [62].

#### Insect feeding and N and cd treatment

Two-leaf stage Zhongmai 175 wheat seedlings were used for adult aphid (non-phytotoxic *S. avenae* and phytotoxic *S. graminum*) infestation [40]. Leaf samples (~ 2.5 × 2.5 cm) from aphid feeding sites were used for RNA-seq. Information on DEGs between control and treated samples were obtained from the additional files of a previously published study [40].

Two-leaf stage Wanmai No. 52 seedlings were used for N stress treatment. The roots and leaves were sampled for transcriptome analysis at 10 d after treatment [63]. We downloaded the DEGs related to N stress from a previous study [63].

For cadmium (Cd) stress treatment, 50 μM CdCl_2_ was applied to Zhengmai 379 seedlings. The roots of seedlings were harvested at 12 d after Cd treatment and used for transcriptome sequencing [41]. The identified DEGs were obtained from a previous study [41].

For the various wheat and maize genome versions, we converted gene IDs of the above wheat genes in the website (http://202.194.139.32/idConvert/) and maize gene IDs in the MaizeGDB database (https://chinese.maizegdb.org/gene_center/gene).

## Supplementary Information

**Additional file 1: Figure S1.** Chromosome location analysis of *HM* genes.

**Additional file 2: Figure S2.** Conserved domain analysis of HM proteins.

**Additional file 3: Figure S3.** Phylogenetic analysis of *HM* genes.

**Additional file 4: Figure S4.** Synteny analysis of each Gramineae *HM* gene.

**Additional file 5: Figure S5.** Synteny analysis of *HM* genes between each Gramineae species and *Arabidopsis*.

**Additional file 6: Figure S6.** Synteny analysis of *HM* genes between each Gramineae species and rice.

**Additional file 7: Figure S7.** Promoter analysis of *T. aestivum*, *H. vulgare*, *S. bicolor*, *S. viridis*, *S. italica*, and *Z. mays HM* genes.

**Additional file 8: Figure S8.** Gene structure analysis of *HM* genes.

**Additional file 9: Table S1.** List of *HM* gene families in *Arabidopsis*, *O. sativa* (rice), *T. aestivum*, *H. vulgare*, *S. bicolor*, *S. viridis*, *S. italica*, and *Z. mays* genomes. **Table S2.** Ka, Ks, and Ka/Ks values of each Gramineae duplication gene pair. **Table S3.** Ka, Ks, and Ka/Ks values of duplication gene pairs between *Arabidopsis* and each Gramineae species. **Table S4.** Ka, Ks, and Ka/Ks values of duplication gene pairs between rice and each Gramineae species. **Table S5.** Common DEGs among different classes.

## Data Availability

All RNA-seq data mentioned here can be found in previous studies [33, 37, 38, 40–43, 58–62]. The datasets used and/or analyzed during the current study are also available from the corresponding author on reasonable request.

## Abbreviations

HATs: Histone acetylases
HDACs: Histone deacetylases
HDMs: Histone methylases
HM: Histone modification
HMTs: Histone methyltransferases
FPKM: Fragments per kilobase per million
HS: Heat stress
DS: Drought stress
MD: Moderate drought
SD: Severe drought
*S. avenae*: *Sitobion avenae*
*S. graminum*: *Schizaphis graminum*
IWGSC: International Wheat Genome Sequencing Consortium

## References

[1] Fan S, Liu H, Liu J, Hua W, Xu S, Li J. Systematic analysis of the DNA Methylase and Demethylase gene families in rapeseed (Brassica napus L.) and their expression variations after salt and heat stresses. Int J Mol Sci. 2020;21(3):953–63. 10.3390/ijms21030953 PMC7036824 32023925

[2] Ho L, Crabtree GR. Chromatin remodelling during development. Nature. 2010;463(7280):474–84. 10.1038/nature08911 PMC3060774 20110991

[3] Sun Z, Wang X, Qiao K, Fan S, Ma Q (2021). Genome-wide analysis of JMJ-C histone demethylase family involved in salt-tolerance in Gossypium hirsutum L. Plant Physiol Biochem.

[4] Zhang X (2008). The epigenetic landscape of plants. Science..

[5] Klose RJ, Zhang Y (2007). Regulation of histone methylation by demethylimination and demethylation. Nat Rev Mol Cell Biol.

[6] Xu J, Xu H, Liu Y, Wang X, Xu Q, Deng X (2015). Genome-wide identification of sweet orange (Citrus sinensis) histone modification gene families and their expression analysis during the fruit development and fruit-blue mold infection process. Front Plant Sci.

[7] Aiese CR, Sanseverino W, Cremona G, Ercolano MR, Conicella C, Consiglio FM (2013). Genome-wide analysis of histone modifiers in tomato: gaining an insight into their developmental roles. BMC Genomics.

[8] Chen DH, Qiu HL, Huang Y, Zhang L, Si JP (2020). Genome-wide identification and expression profiling of SET DOMAIN GROUP family in Dendrobium catenatum. BMC Plant Biol.

[9] Ahmad A, Cao X (2012). Plant PRMTs broaden the scope of arginine methylation. J Genet Genomics.

[10] Dong G, Ma DP, Li J (2008). The histone methyltransferase SDG8 regulates shoot branching in Arabidopsis. Biochem Biophys Res Commun.

[11] Jiang D, Yang W, He Y, Amasino RM (2007). Arabidopsis relatives of the human lysine-specific Demethylase1 repress the expression of FWA and FLOWERING LOCUS C and thus promote the floral transition. Plant Cell.

[12] Chen X, Hu Y, Zhou DX (2011). Epigenetic gene regulation by plant Jumonji group of histone demethylase. Biochim Biophys Acta.

[13] Xing L, Qi S, Zhou H, Zhang W, Zhang C, Ma W, Zhang Q, Shah K, Han M, Zhao J (2020). Epigenomic regulatory mechanism in vegetative phase transition of Malus hupehensis. J Agric Food Chem.

[14] Pandey R, Muller A, Napoli CA, Selinger DA, Pikaard CS, Richards EJ, Bender J, Mount DW, Jorgensen RA (2002). Analysis of histone acetyltransferase and histone deacetylase families of Arabidopsis thaliana suggests functional diversification of chromatin modification among multicellular eukaryotes. Nucleic Acids Res.

[15] Chen ZJ, Tian L (2007). Roles of dynamic and reversible histone acetylation in plant development and polyploidy. Biochim Biophys Acta.

[16] Sheldon CC, Finnegan EJ, Dennis ES, Peacock WJ (2006). Quantitative effects of vernalization on FLC and SOC1 expression. Plant J.

[17] Tian L, Fong MP, Wang JJ, Wei NE, Jiang H, Doerge RW, Chen ZJ (2005). Reversible histone acetylation and deacetylation mediate genome-wide, promoter-dependent and locus-specific changes in gene expression during plant development. Genetics..

[18] Wang L, Ahmad B, Liang C, Shi X, Sun R, Zhang S, Du G (2020). Bioinformatics and expression analysis of histone modification genes in grapevine predict their involvement in seed development, powdery mildew resistance, and hormonal signaling. BMC Plant Biol.

[19] Hollender C, Liu Z (2008). Histone deacetylase genes in Arabidopsis development. J Integr Plant Biol.

[20] Ma X, Lv S, Zhang C, Yang C (2013). Histone deacetylases and their functions in plants. Plant Cell Rep.

[21] Wang Z, Cao H, Chen F, Liu Y (2014). The roles of histone acetylation in seed performance and plant development. Plant Physiol Biochem.

[22] Appels R, Eversole K, Feuillet C, Keller B, Rogers J, Stein N, et al. Shifting the limits in wheat research and breeding using a fully annotated reference genome. Science. 2018;361(6403):7191. 10.1126/science.aar7191 30115783

[23] Steele KA, Price AH, Witcombe JR, Shrestha R, Singh BN, Gibbons JM, Virk DS (2013). QTLs associated with root traits increase yield in upland rice when transferred through marker-assisted selection. Theor Appl Genet.

[24] Uga Y, Sugimoto K, Ogawa S, Rane J, Ishitani M, Hara N, Kitomi Y, Inukai Y, Ono K, Kanno N (2013). Control of root system architecture by DEEPER ROOTING 1 increases rice yield under drought conditions. Nat Genet.

[25] Chen D, Chen D, Xue R, Long J, Lin X, Lin Y, Jia L, Zeng R, Song Y (2019). Effects of boron, silicon and their interactions on cadmium accumulation and toxicity in rice plants. J Hazard Mater.

[26] Hanin M, Ebel C, Ngom M, Laplaze L, Masmoudi K (2016). New insights on plant salt tolerance mechanisms and their potential use for breeding. Front Plant Sci.

[27] Wang X, Xin C, Cai J, Zhou Q, Dai T, Cao W, Jiang D (2016). Heat priming induces trans-generational tolerance to high temperature stress in wheat. Front Plant Sci.

[28] Xie W, Xiong W, Pan J, Ali T, Cui Q, Guan D, Meng J, Mueller ND, Lin E, Davis SJ (2018). Decreases in global beer supply due to extreme drought and heat. Nature Plants.

[29] Zorb C, Geilfus CM, Dietz KJ (2019). Salinity and crop yield. Plant Biol (Stuttg).

[30] Agarwal P, Khurana P (2019). Functional characterization of HSFs from wheat in response to heat and other abiotic stress conditions. Funct Integr Genomics.

[31] Chinnusamy V, Zhu JK (2009). Epigenetic regulation of stress responses in plants. Curr Opin Plant Biol.

[32] Choi SM, Song HR, Han SK, Han M, Kim CY, Park J, Lee YH, Jeon JS, Noh YS, Noh B (2012). HDA19 is required for the repression of salicylic acid biosynthesis and salicylic acid-mediated defense responses in Arabidopsis. Plant J.

[33] Hou L, Zhang A, Wang R, Zhao P, Zhang D, Jiang Y, Diddugodage CJ, Wang X, Ni Z, Xu S (2019). Brassinosteroid regulates root development with highly redundant genes in Hexaploid wheat. Plant Cell Physiol.

[34] Konishi H, Komatsu S (2003). A proteomics approach to investigating promotive effects of brassinolide on lamina inclination and root growth in rice seedlings. Biol Pharm Bull.

[35] Wei Z, Li J (2016). Brassinosteroids regulate root growth, development, and Symbiosis. Mol Plant.

[36] Kim SK, Chang SC, Lee EJ, Chung WS, Kim YS, Hwang S, Lee JS (2000). Involvement of brassinosteroids in the gravitropic response of primary root of maize. Plant Physiol.

[37] Dong FS, Lv MY, Wang JP, Shi XP, Liang XX, Liu YW, Yang F, Zhao H, Chai JF, Zhou S (2020). Transcriptome analysis of activated charcoal-induced growth promotion of wheat seedlings in tissue culture. BMC Genet.

[38] Liu Z, Xin M, Qin J, Peng H, Ni Z, Yao Y, Sun Q (2015). Temporal transcriptome profiling reveals expression partitioning of homeologous genes contributing to heat and drought acclimation in wheat (Triticum aestivum L.). Bmc. Plant Biol.

[39] Hu XS, Liu XF, Thieme T, Zhang GS, Liu TX, Zhao HY (2015). Testing the fecundity advantage hypothesis with Sitobion avenae, Rhopalosiphum padi, and Schizaphis graminum (Hemiptera: Aphididae) feeding on ten wheat accessions. Sci Rep.

[40] Zhang Y, Fu Y, Fan J, Li Q, Francis F, Chen J (2019). Comparative transcriptome and histological analyses of wheat in response to phytotoxic aphid Schizaphis graminum and non-phytotoxic aphid Sitobion avenae feeding. BMC Plant Biol.

[41] Qin S, Liu H, Rengel Z, Gao W, Nie Z, Li C, Hou M, Cheng J, Zhao P (2020). Boron inhibits cadmium uptake in wheat (Triticum aestivum) by regulating gene expression. Plant Sci.

[42] Gu W, Yu D, Guan Y, Wang H, Qin T, Sun P, Hu Y, Wei J, Zheng H (2020). The dynamic transcriptome of waxy maize (Zea mays L. sinensis Kulesh) during seed development. Genes. Genomics..

[43] Wang Y, Wang X, Deng D, Wang Y (2019). Maize transcriptomic repertoires respond to gibberellin stimulation. Mol Biol Rep.

[44] Lu F, Li G, Cui X, Liu C, Wang XJ, Cao X (2008). Comparative analysis of JmjC domain-containing proteins reveals the potential histone demethylases in Arabidopsis and rice. J Integr Plant Biol.

[45] Xu G, Guo C, Shan H, Kong H (2012). Divergence of duplicate genes in exon-intron structure. Proc Natl Acad Sci U S A.

[46] Berr A, McCallum EJ, Alioua A, Heintz D, Heitz T, Shen WH (2010). Arabidopsis histone methyltransferase SET DOMAIN GROUP8 mediates induction of the jasmonate/ethylene pathway genes in plant defense response to necrotrophic fungi. Plant Physiol.

[47] Kim JM, To TK, Nishioka T, Seki M (2010). Chromatin regulation functions in plant abiotic stress responses. Plant Cell Environ.

[48] Luo M, Liu X, Singh P, Cui Y, Zimmerli L, Wu K (2012). Chromatin modifications and remodeling in plant abiotic stress responses. Biochim Biophys Acta.

[49] Lynch M, Conery JS (2000). The evolutionary fate and consequences of duplicate genes. Science..

[50] Finn RD, Clements J, Eddy SR (2011). HMMER web server: interactive sequence similarity searching. Nucleic Acids Res.

[51] Chen C, Chen H, Zhang Y, Thomas HR, Frank MH, He Y, Xia R (2020). TBtools: An integrative toolkit developed for interactive analyses of big biological data. Mol Plant.

[52] Zheng L, Ma J, Song C, An N, Zhang D, Zhao C, Qi S, Han M (2017). Genome-wide identification and expression profiling analysis of brassinolide signal transduction genes regulating apple tree architecture. Acta Physiol Plant.

[53] Zheng L, Ma J, Song C, Zhang L, Gao C, Zhang D, et al. Genome-wide identification and expression analysis of GRF genes regulating apple tree architecture. Tree Genet Genomes. 2018;14:54–64.

[54] Zheng L, Zhao C, Mao J, Song C, Ma J, Zhang D, Han M, An N (2018). Genome-wide identification and expression analysis of brassinosteroid biosynthesis and metabolism genes regulating apple tree shoot and lateral root growth. J Plant Physiol.

[55] Zheng L, Ma J, Zhang L, Gao C, Zhang D, Zhao C, Han M (2018). Revealing critical mechanisms of BR-mediated apple nursery tree growth using iTRAQ-based proteomic analysis. J Proteome.

[56] Zheng L, Yang Y, Gao C, Ma J, Shah K, Zhang D, Zhao C, Xing L, Han M, An N (2019). Transcriptome analysis reveals new insights into MdBAK1-mediated plant growth in Malus domestica. J Agric Food Chem.

[57] Kumar S, Stecher G, Li M, Knyaz C, Tamura K (2018). MEGA X: molecular evolutionary genetics analysis across computing platforms. Mol Biol Evol.

[58] Wan Y, Underwood C, Toole G, Skeggs P, Zhu T, Leverington M, Griffiths S, Wheeler T, Gooding M, Poole R (2009). A novel transcriptomic approach to identify candidate genes for grain quality traits in wheat. Plant Biotechnol J.

[59] Zhang Q, Liu H, Wu X, Wang W (2020). Identification of drought tolerant mechanisms in a drought-tolerant maize mutant based on physiological, biochemical and transcriptomic analyses. BMC Plant Biol.

[60] Liu G, Zenda T, Liu S, Wang X, Jin H, Dong A, Yang Y, Duan H (2020). Comparative transcriptomic and physiological analyses of contrasting hybrid cultivars ND476 and ZX978 identify important differentially expressed genes and pathways regulating drought stress tolerance in maize. Genes Genomics.

[61] Zhang X, Liu X, Zhang D, Tang H, Sun B, Li C, Hao L, Liu C, Li Y, Shi Y (2017). Genome-wide identification of gene expression in contrasting maize inbred lines under field drought conditions reveals the significance of transcription factors in drought tolerance. PLoS One.

[62] Zhang Y, Liu Z, Khan AA, Lin Q, Han Y, Mu P, Liu Y, Zhang H, Li L, Meng X (2016). Expression partitioning of homeologs and tandem duplications contribute to salt tolerance in wheat (Triticum aestivum L.). Sci Rep.

[63] Wang J, Song K, Sun L, Qin Q, Sun Y, Pan J, et al. Morphological and Transcriptome analysis of wheat seedlings response to low nitrogen stress. Plants (Basel). 2019;8(4):98–108. 10.3390/plants8040098 PMC6524375 30991719

