# Supplementary material for: Genome-wide identification of Gramineae histone modification genes and their potential roles in regulating wheat and maize growth and stress responses
Source: BMC Plant Biol. 2021 Nov 20;21:543. doi: 10.1186/s12870-021-03332-8 (PMC8605605; doi:10.1186/s12870-021-03332-8)

**Figure S1 Chromosome location analysis of *HM* genes.**

Figure S1-1 Chromosome location analysis of *T. aestivum* *HM* genes.


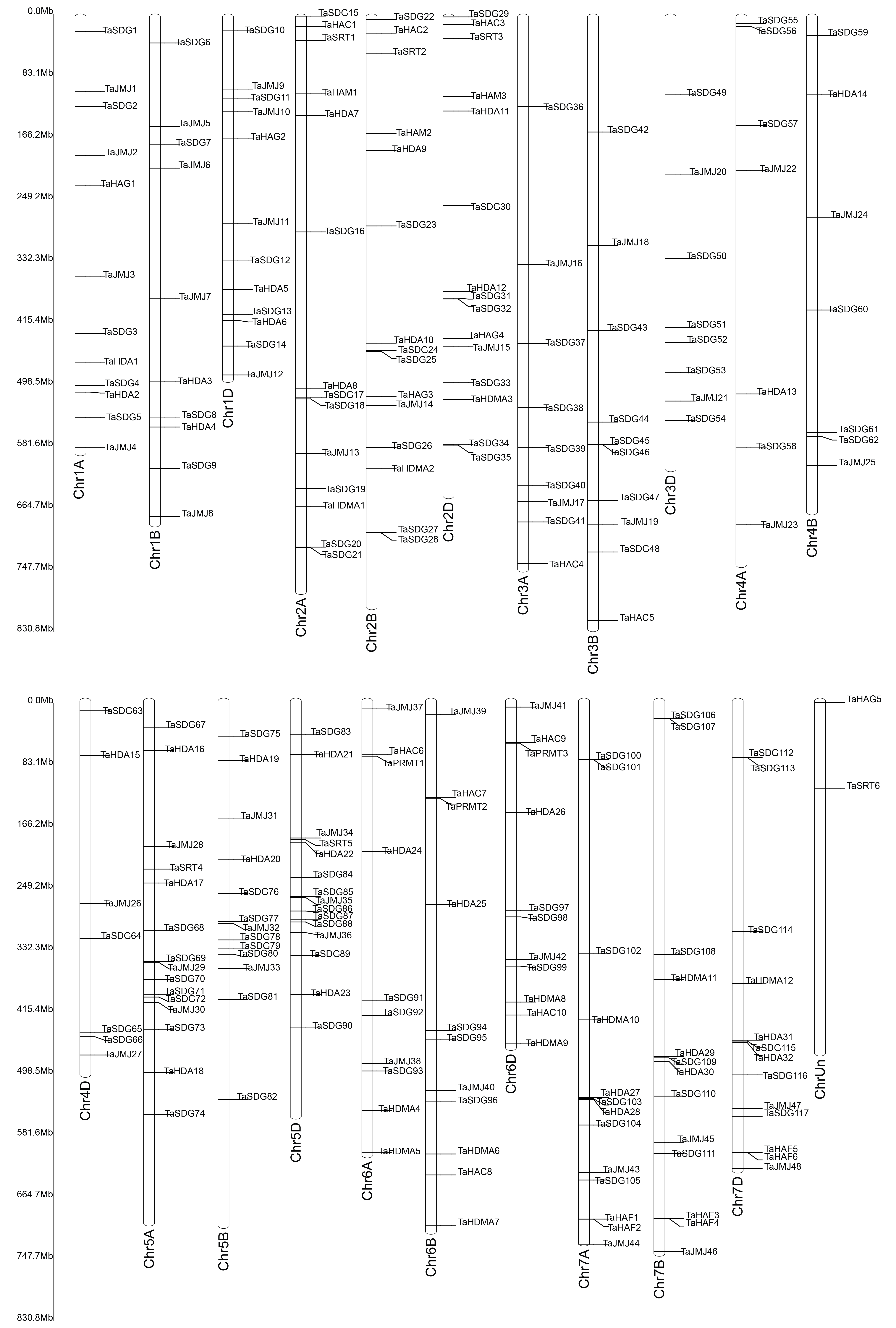


Figure S1-2 Chromosome location analysis of *H. vulgare* *HM* genes.


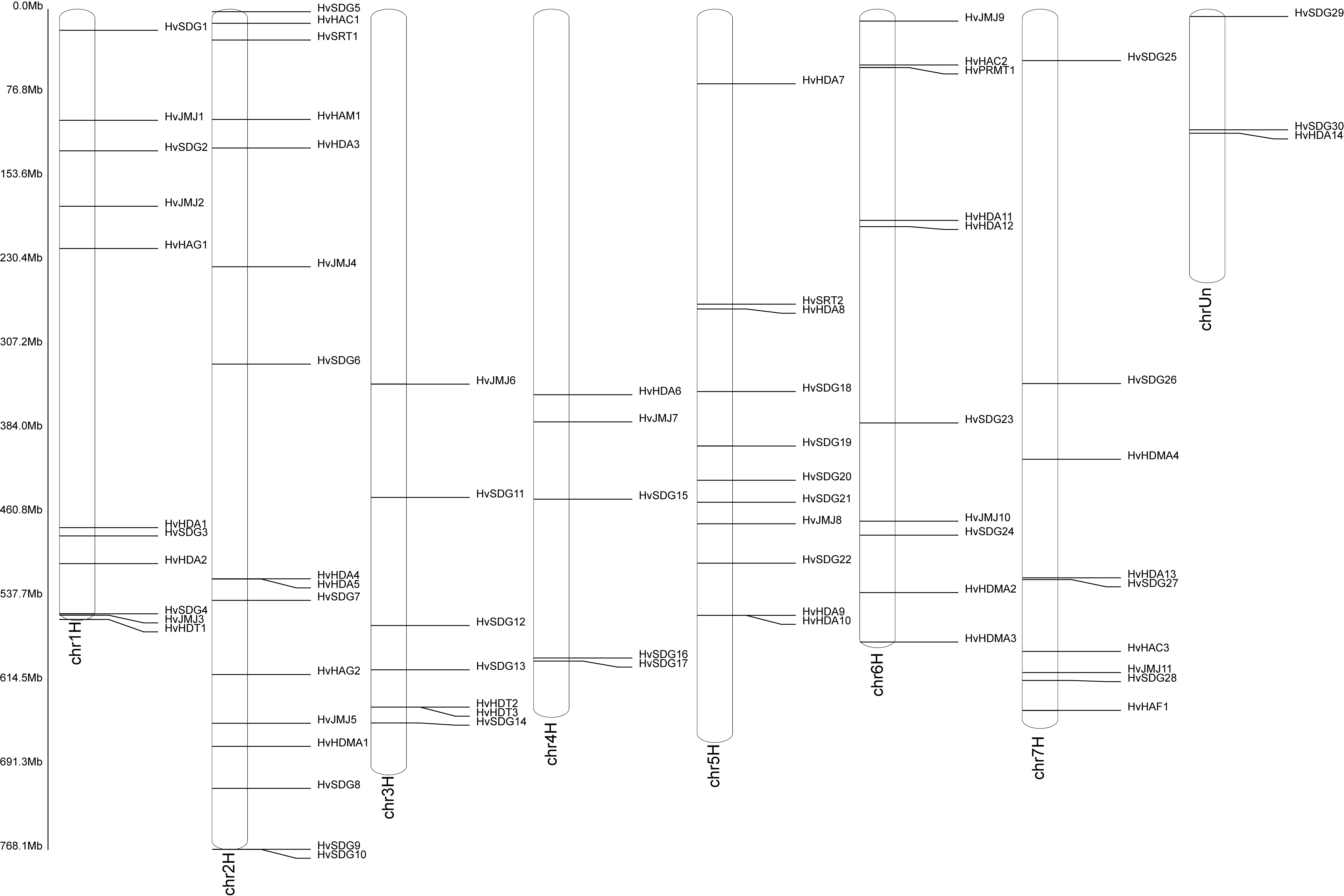


Figure S1-3 Chromosome location analysis of *S. bicolor* *HM* genes.


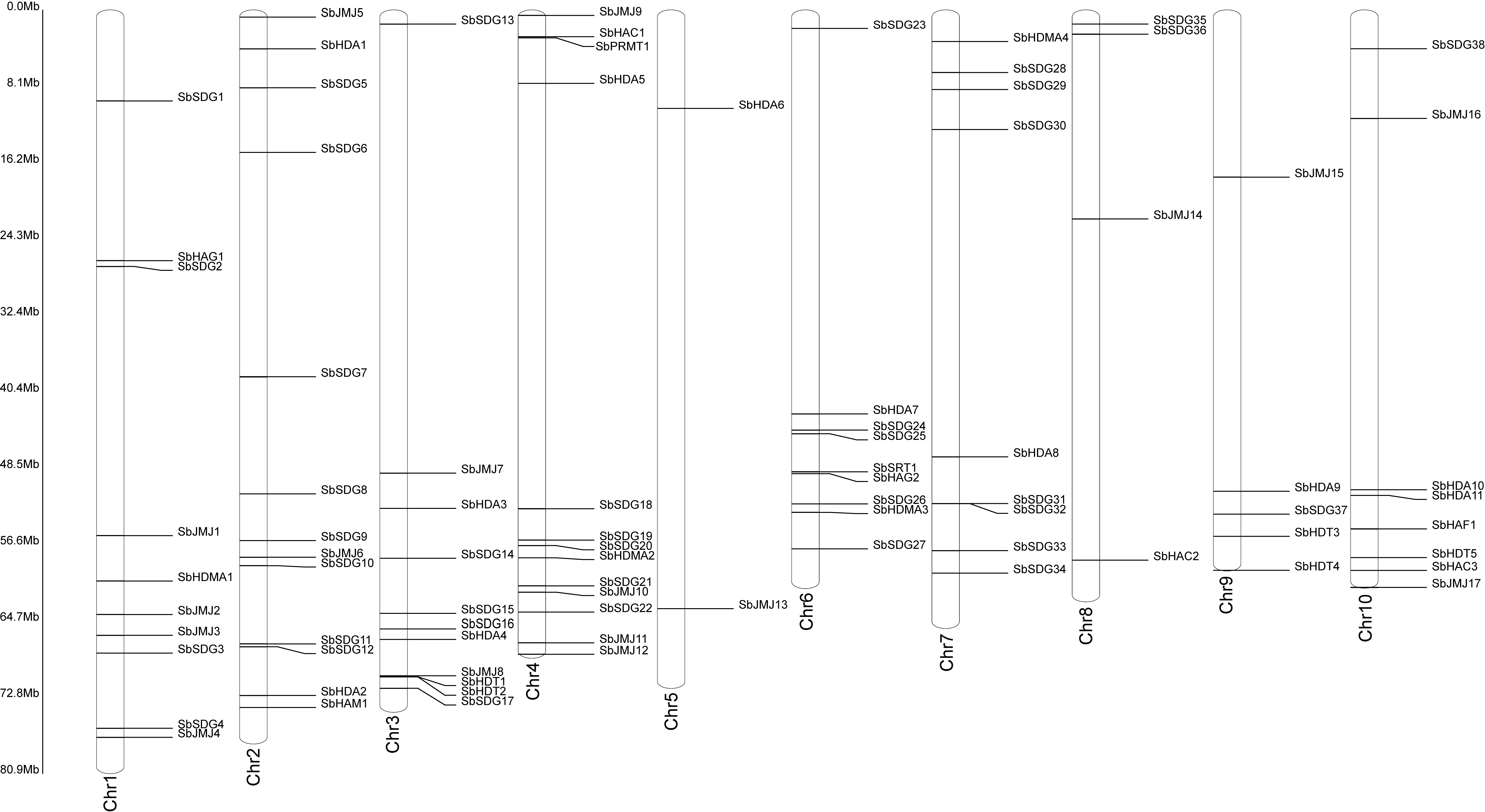


Figure S1-4 Chromosome location analysis of *S. viridis* *HM* genes.


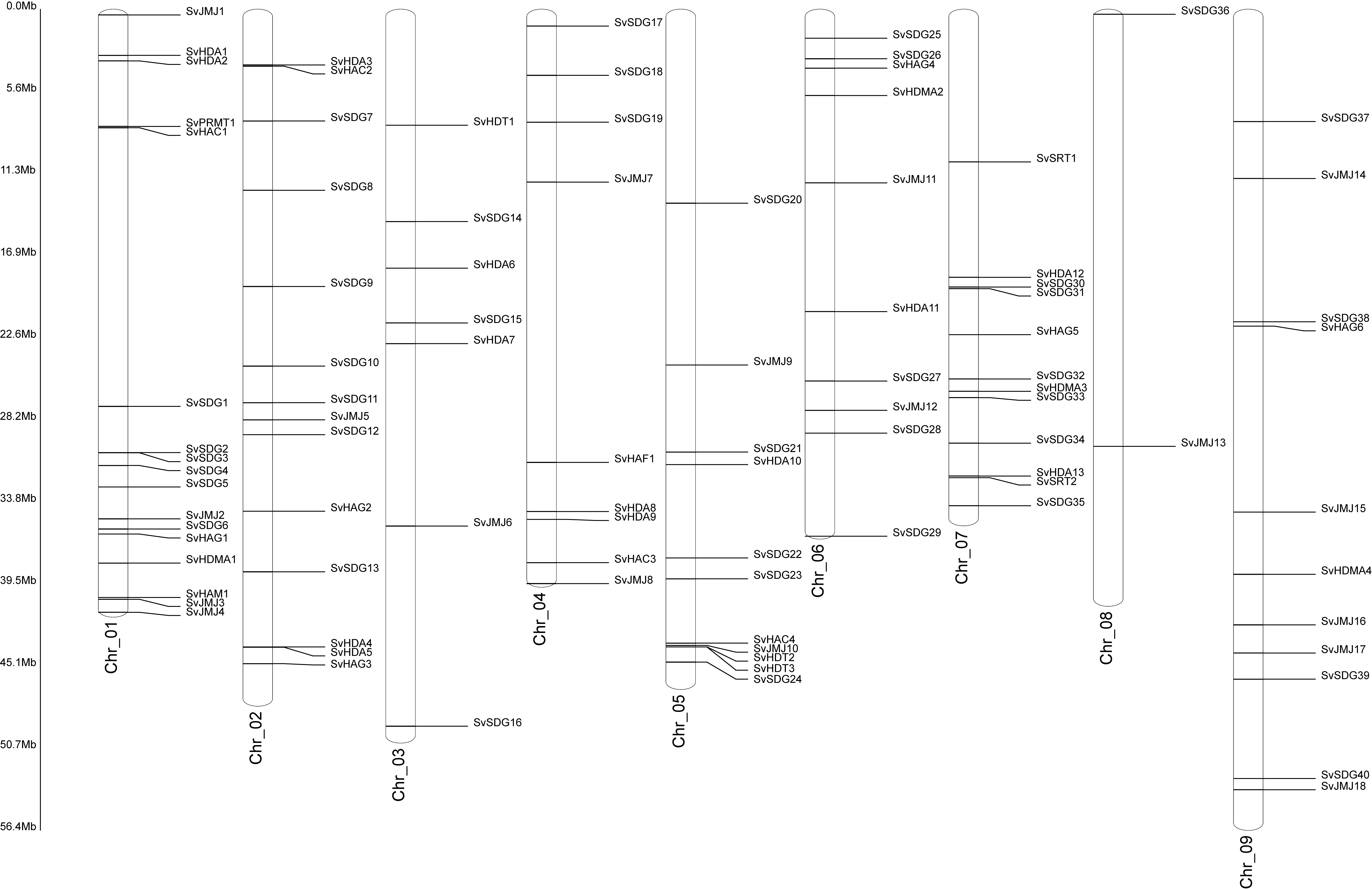


Figure S1-5 Chromosome location analysis of *S. italica* *HM* genes.


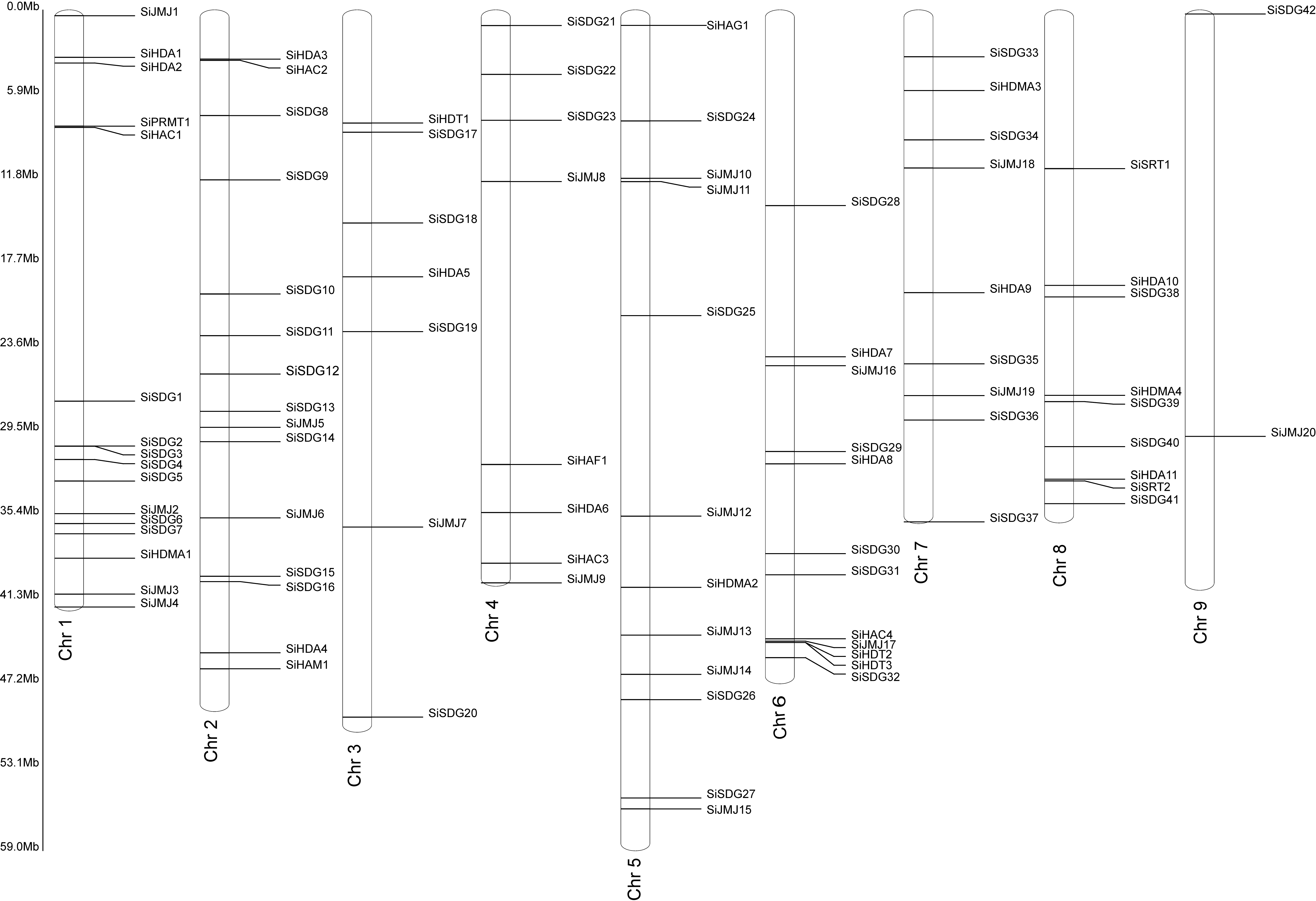


Figure S1-6 Chromosome location analysis of *Z. mays* *HM* genes.


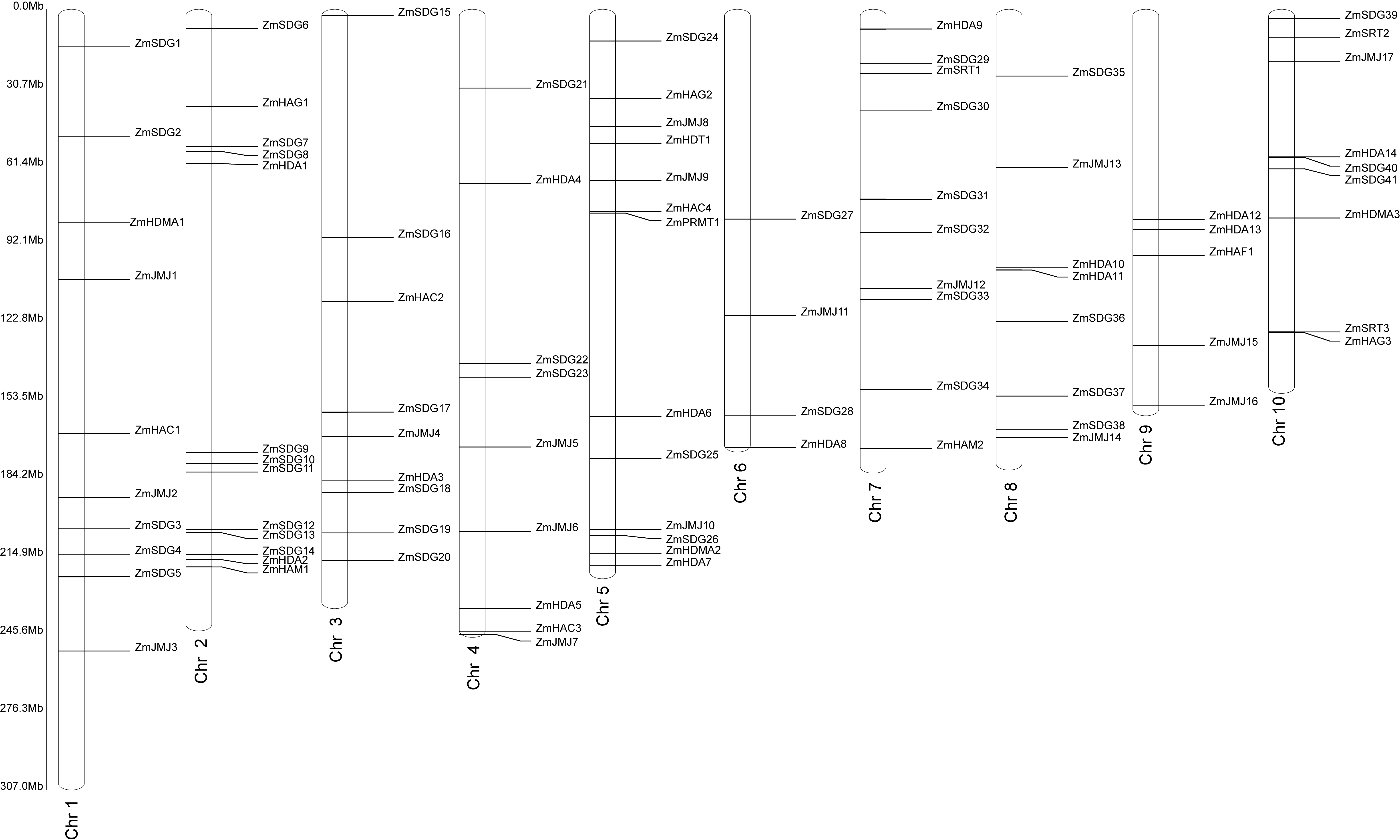

Supplement: Supplementary file 1 — Additional file 1: Figure S1. Chromosome location analysis of HM genes. [file 12870_2021_3332_MOESM1_ESM.docx]
