# Supplementary material for: Genome-wide identification of Gramineae histone modification genes and their potential roles in regulating wheat and maize growth and stress responses
Source: BMC Plant Biol. 2021 Nov 20;21:543. doi: 10.1186/s12870-021-03332-8 (PMC8605605; doi:10.1186/s12870-021-03332-8)

**Figure S2 Conserved domain analysis of HM proteins.**

Figure S2-1 Conserved domain analysis of *Arabidopsis* and rice SDG proteins.


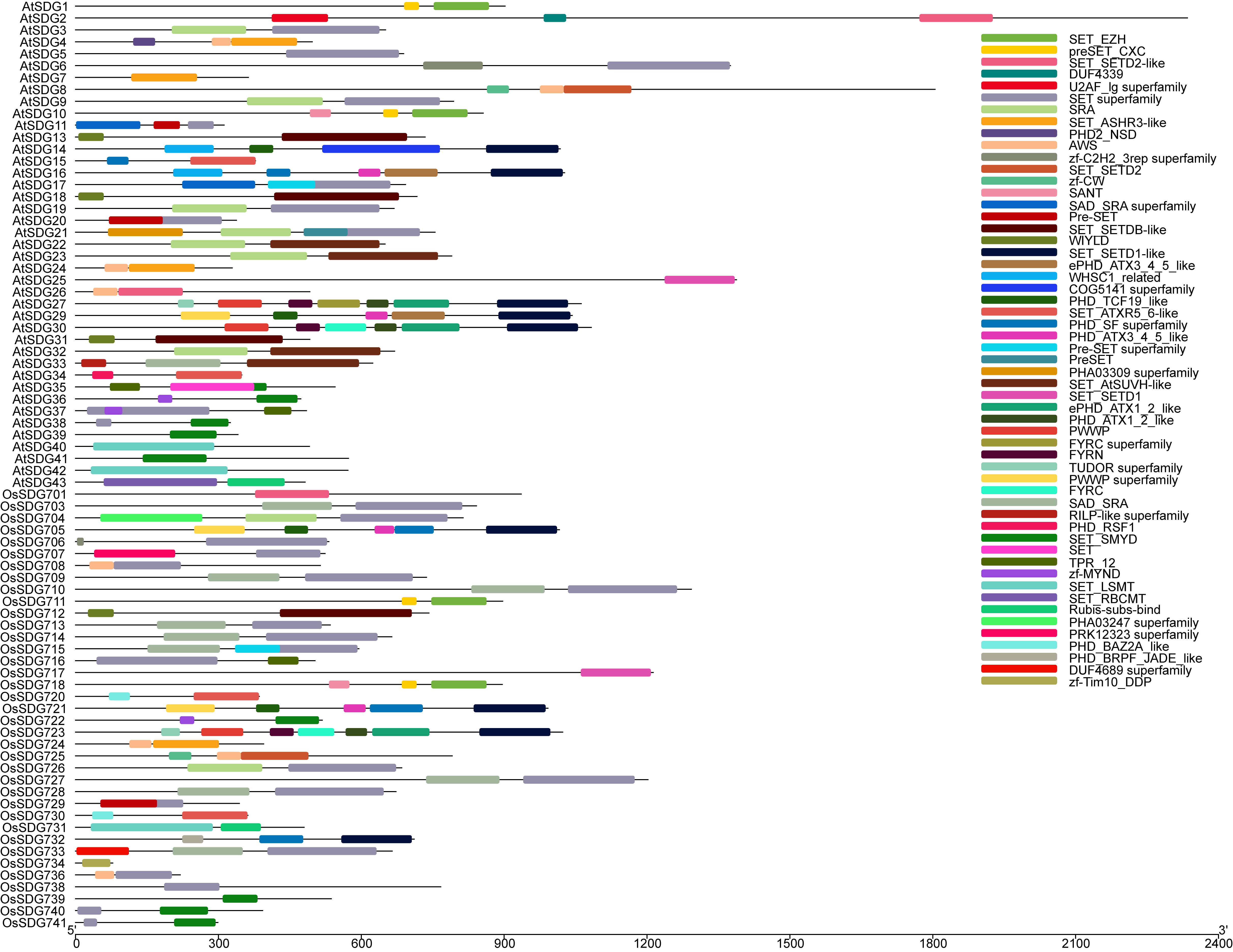


Figure S2-2 Conserved domain analysis of TaSDG proteins.


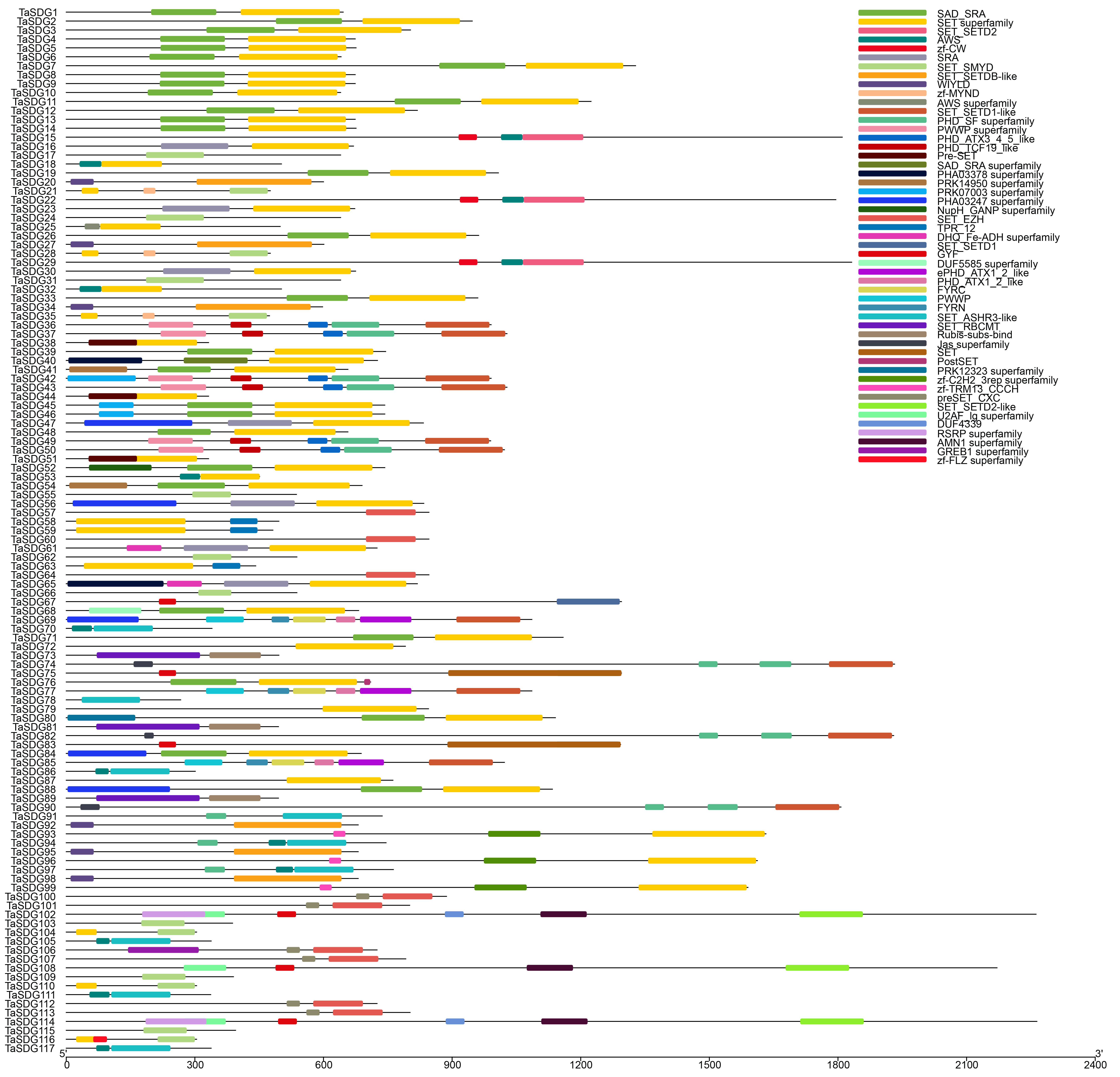


Figure S2-3 Conserved domain analysis of HvSDG proteins.


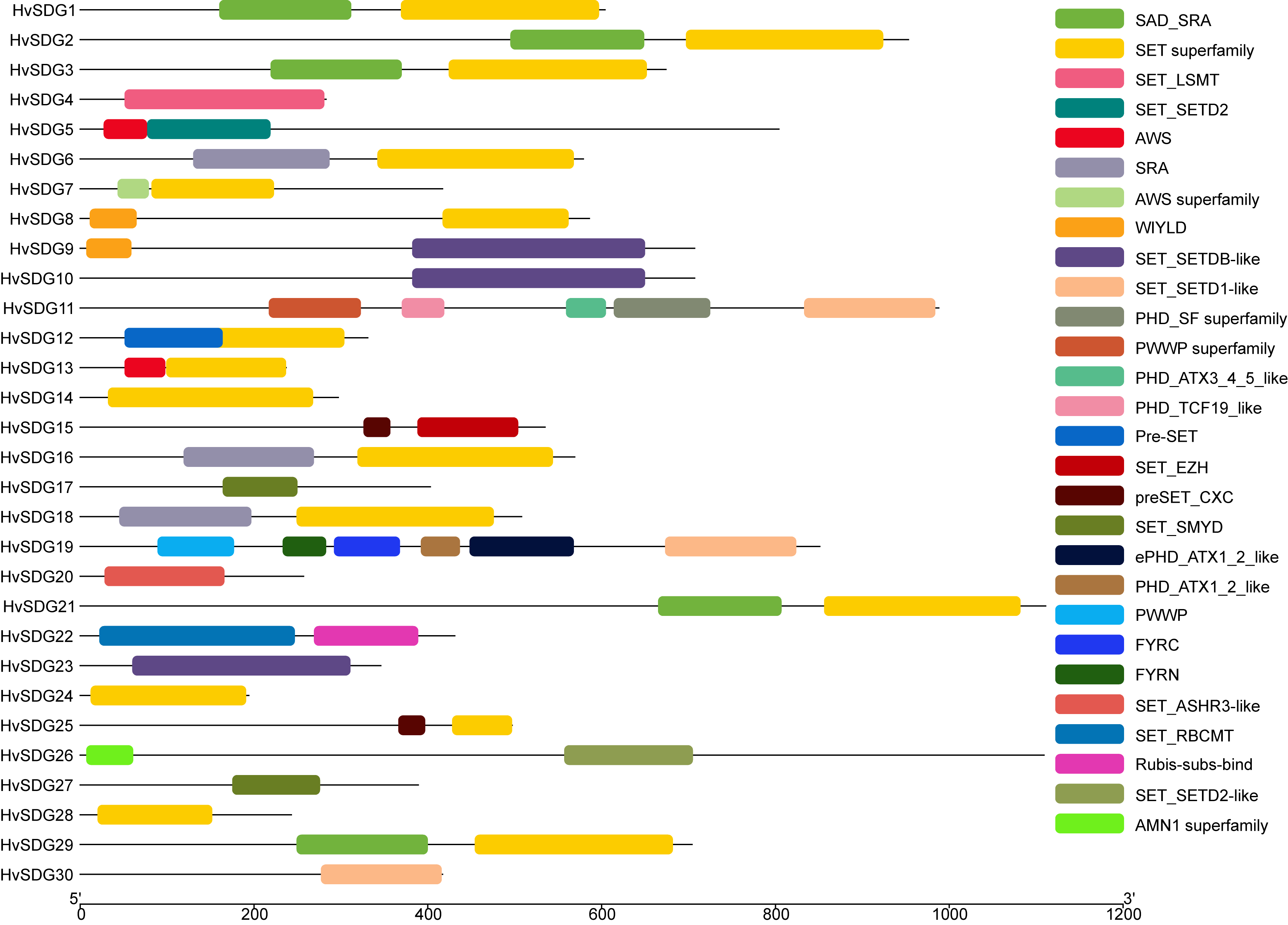


Figure S2-4 Conserved domain analysis of SbSDG proteins.


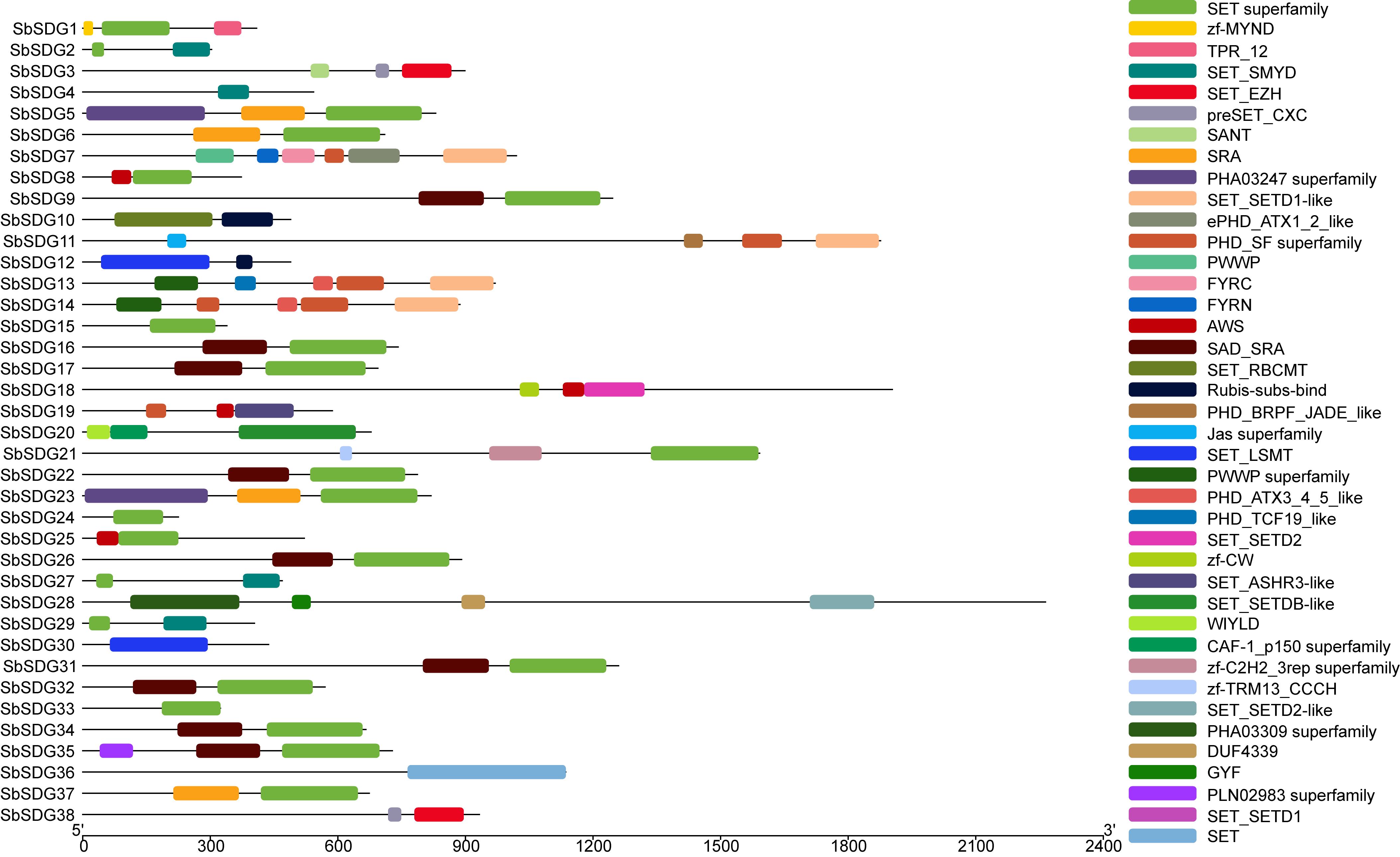


Figure S2-5 Conserved domain analysis of SvSDG proteins.


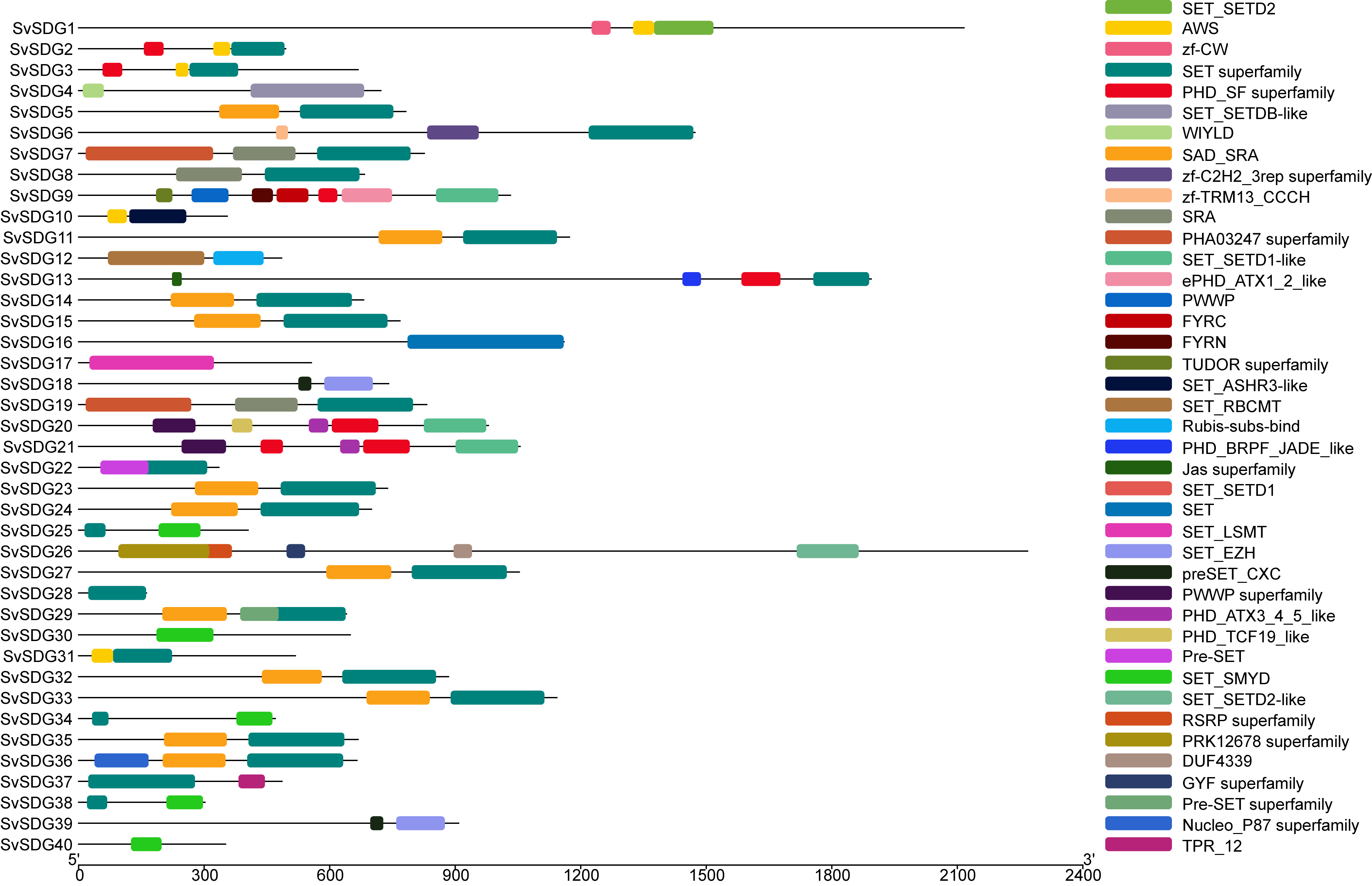


Figure S2-6 Conserved domain analysis of SiSDG proteins.


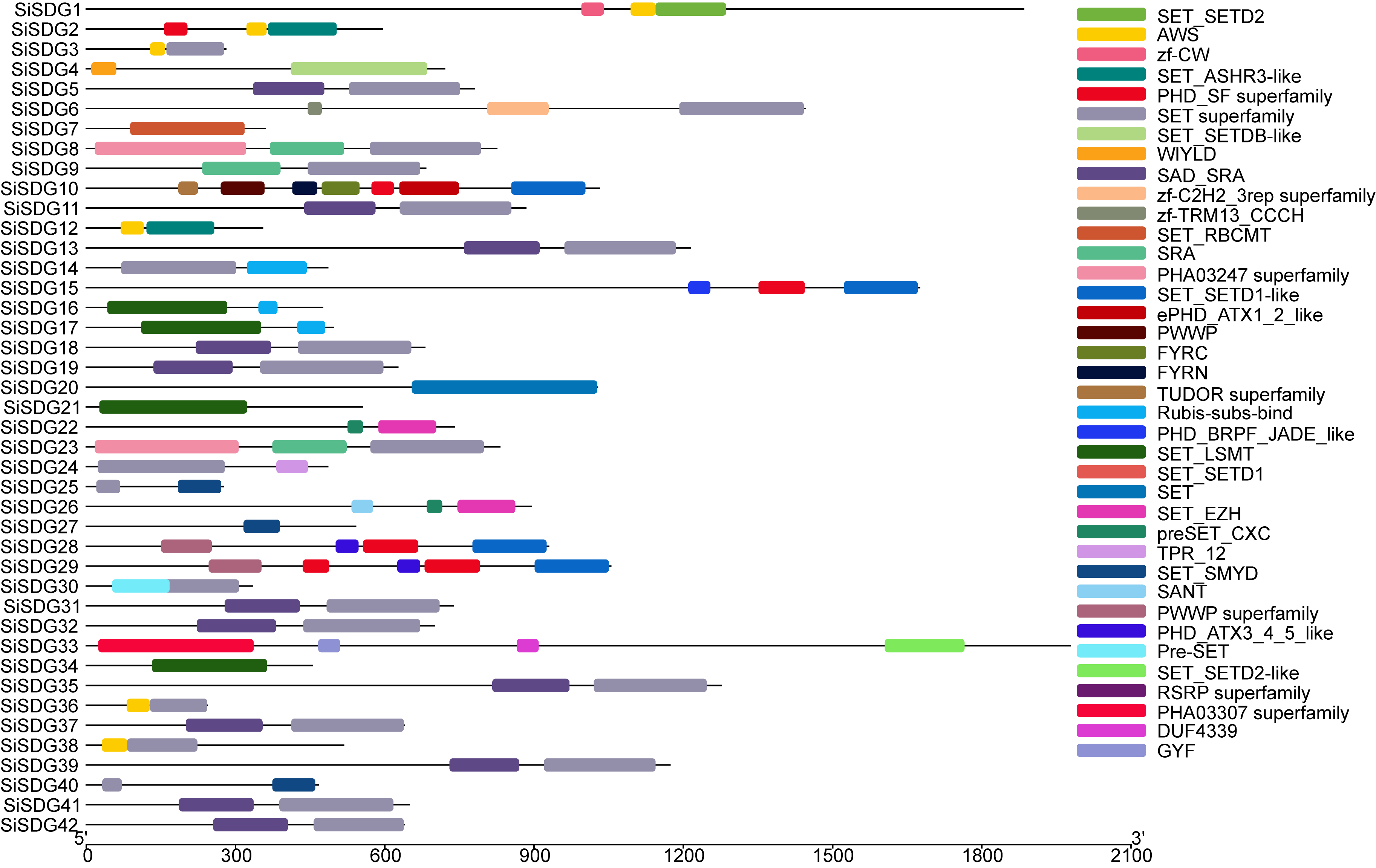


Figure S2-7 Conserved domain analysis of ZmSDG proteins.


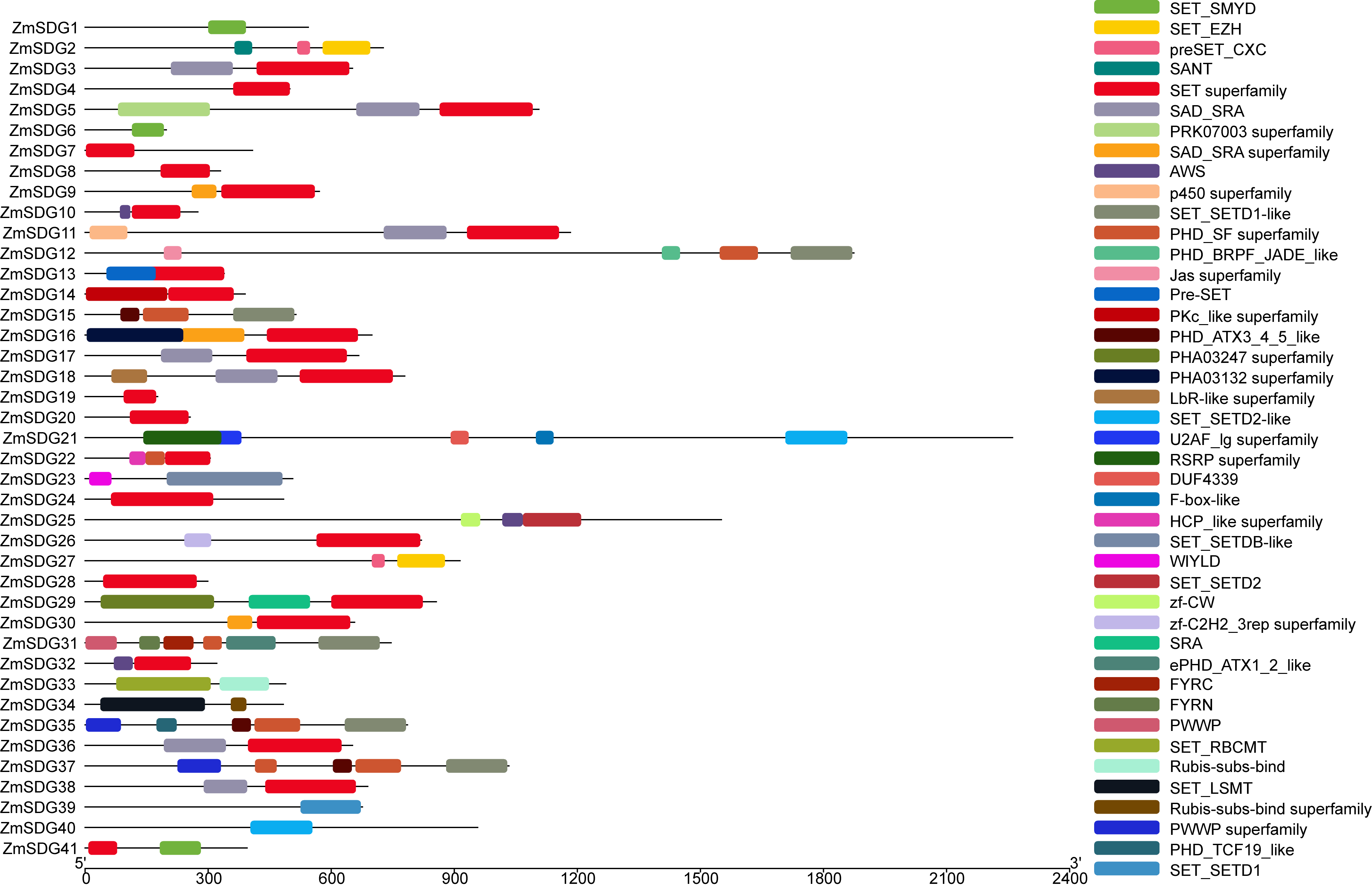


Figure S2-8 Conserved domain analysis of PRMT proteins.


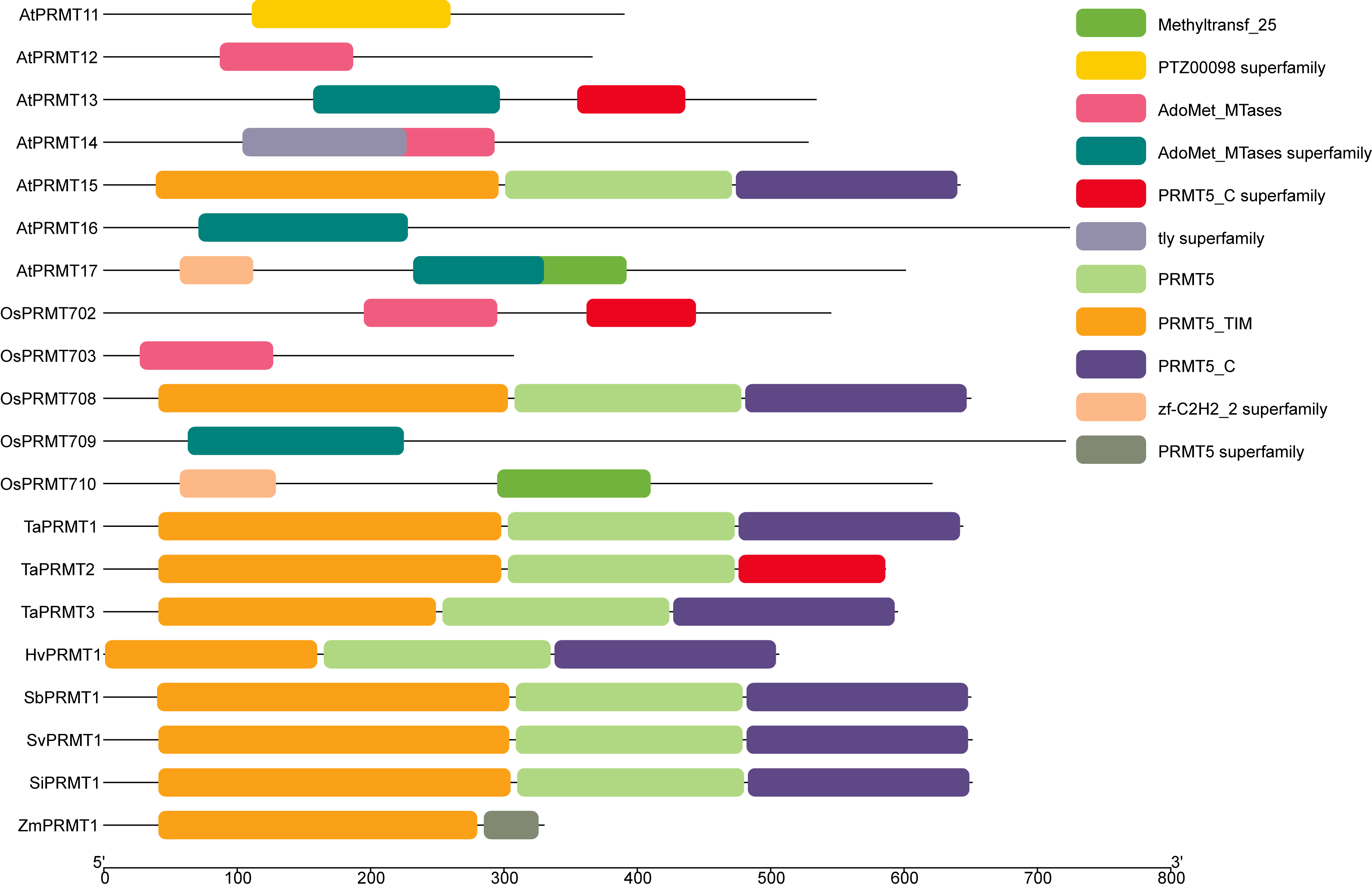


Figure S2-9 Conserved domain analysis of HDMA proteins.


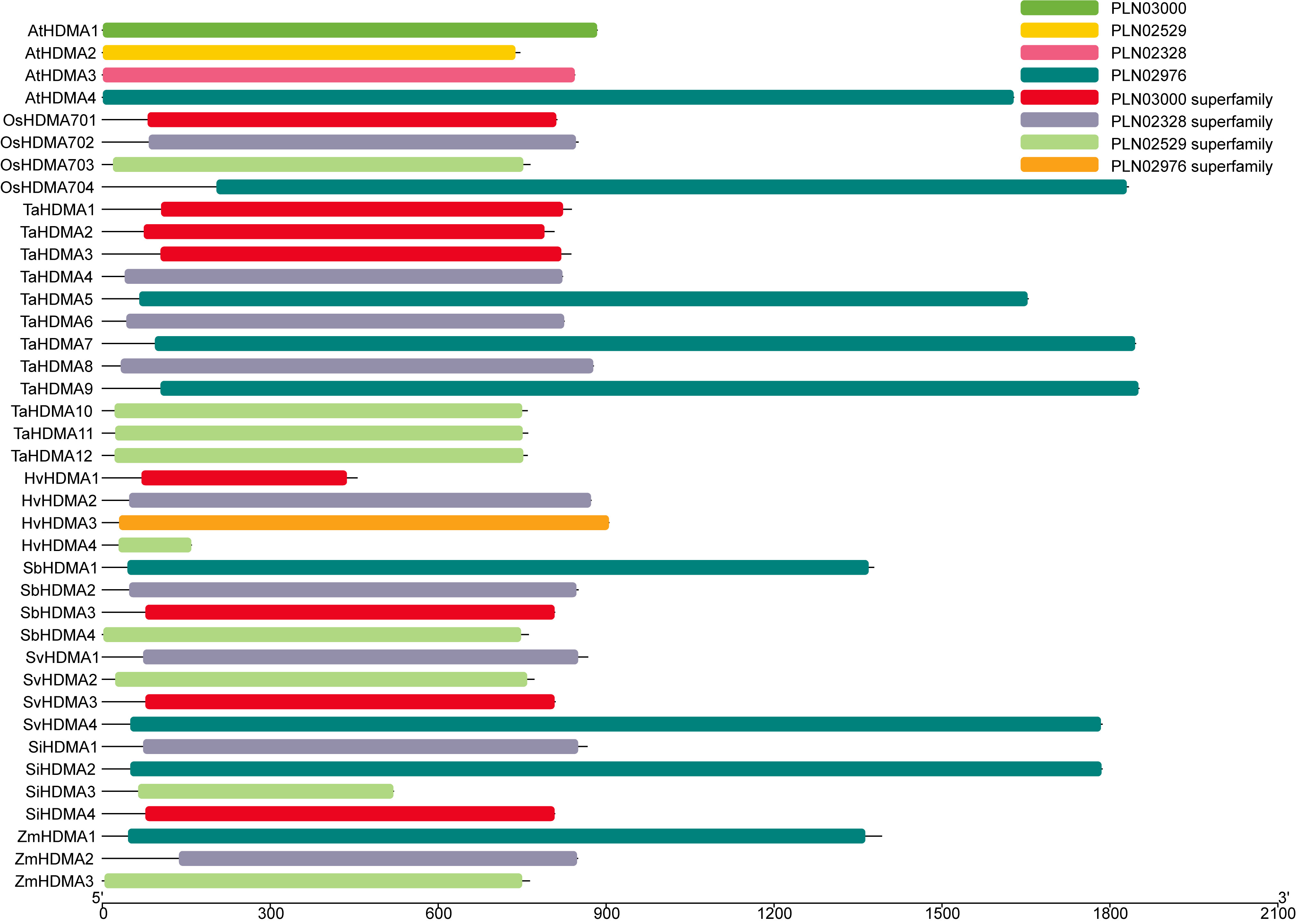


Figure S2-10 Conserved domain analysis of *Arabidopsis* and rice JMJ proteins.


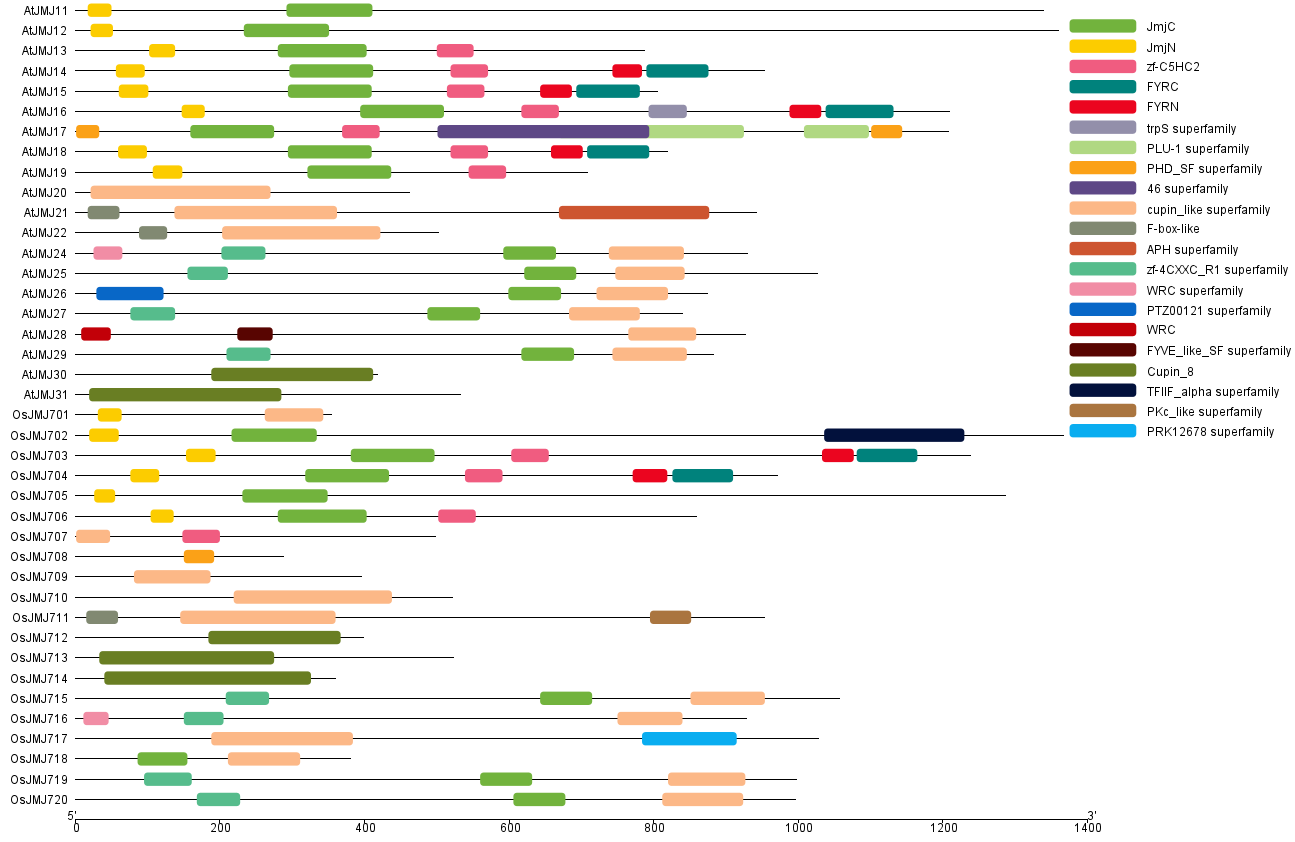


Figure S2-11 Conserved domain analysis of TaJMJ proteins.


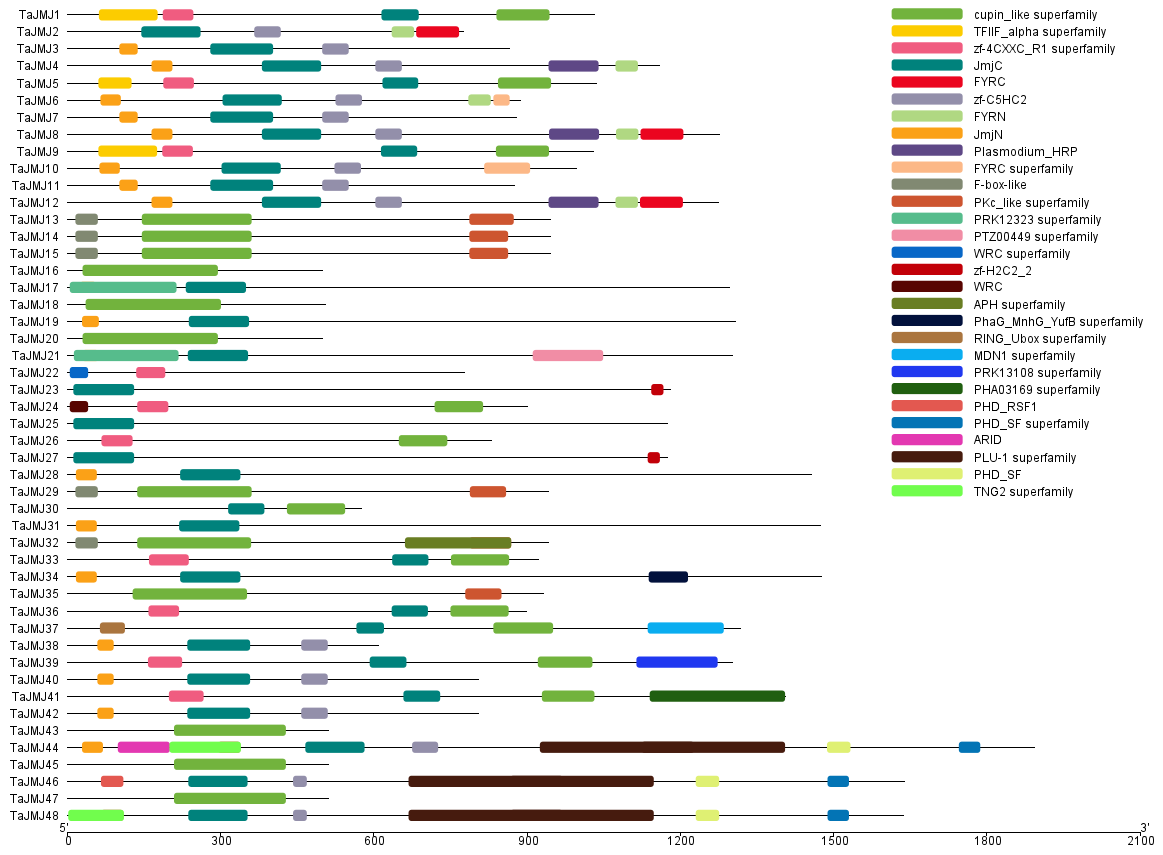


Figure S2-12 Conserved domain analysis of HvJMJ proteins.


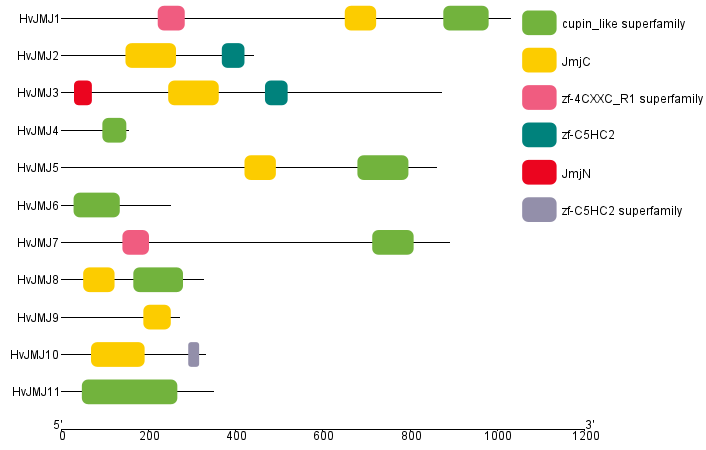


Figure S2-13 Conserved domain analysis of SbJMJ proteins.


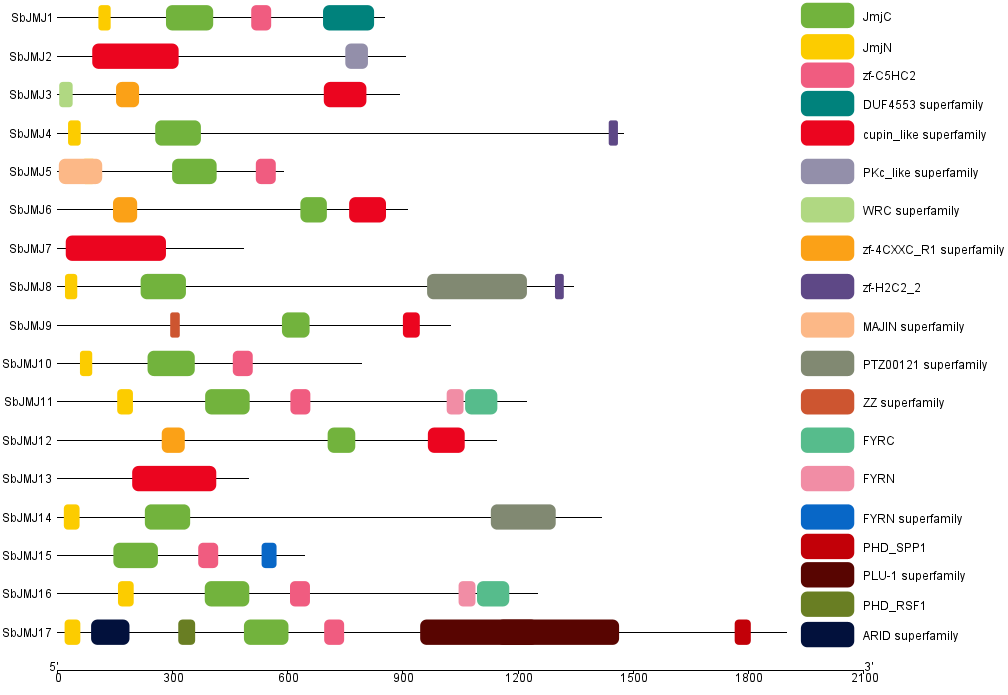


Figure S2-14 Conserved domain analysis of SvJMJ proteins.


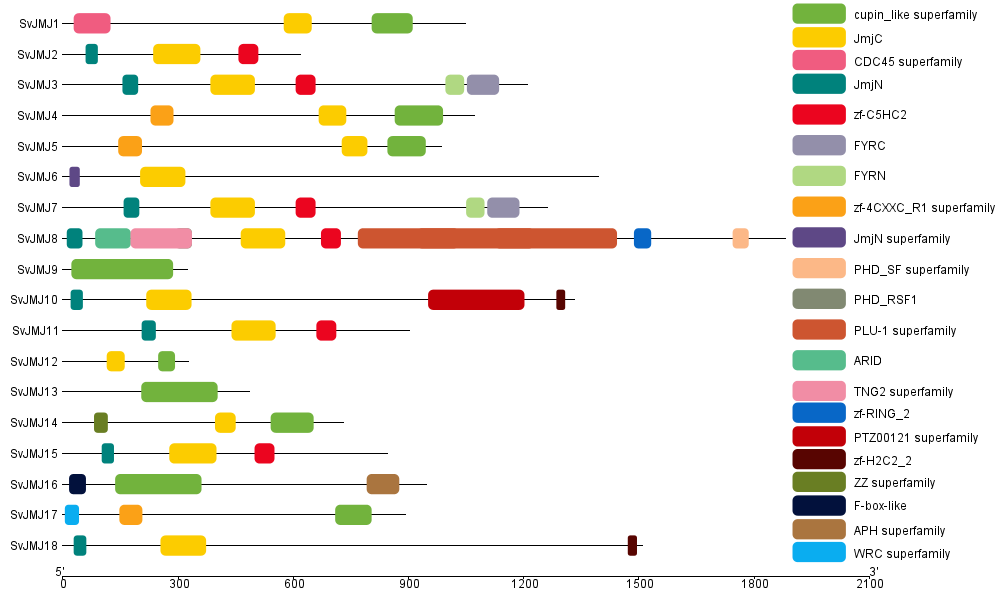


Figure S2-15 Conserved domain analysis of SiJMJ proteins.


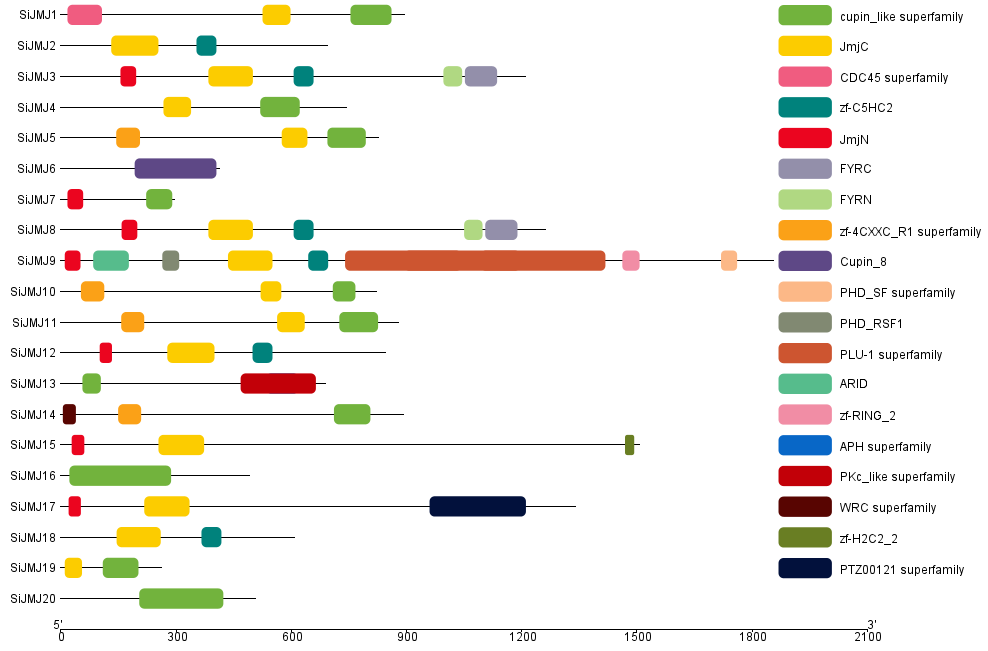


Figure S2-16 Conserved domain analysis of ZmJMJ proteins.


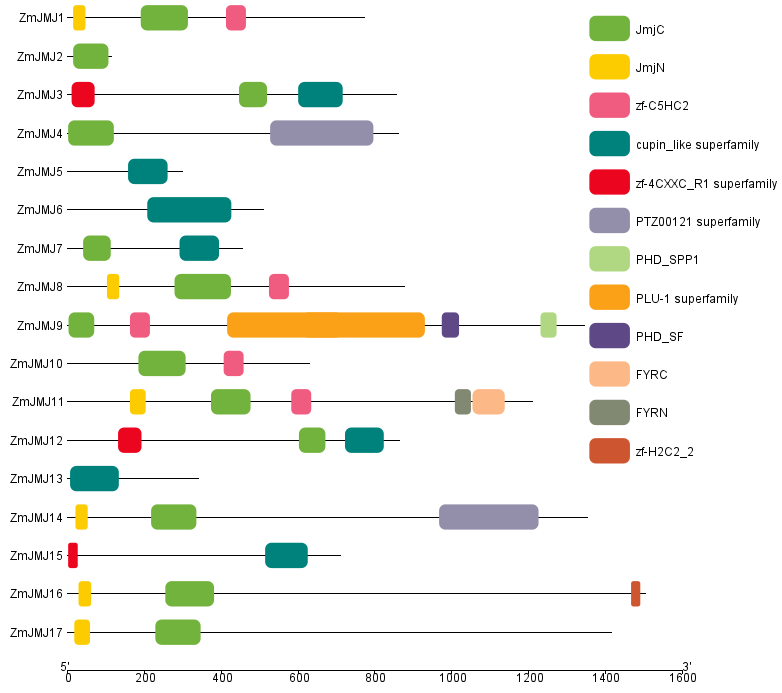


Figure S2-17 Conserved domain analysis of HAG proteins.


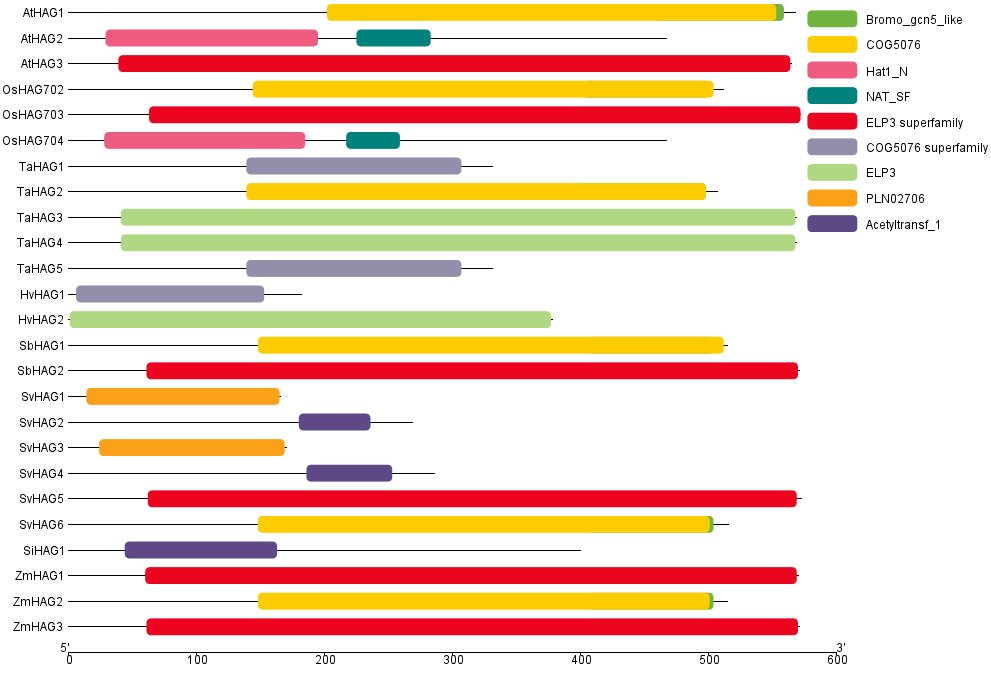


Figure S2-18 Conserved domain analysis of HAM proteins.


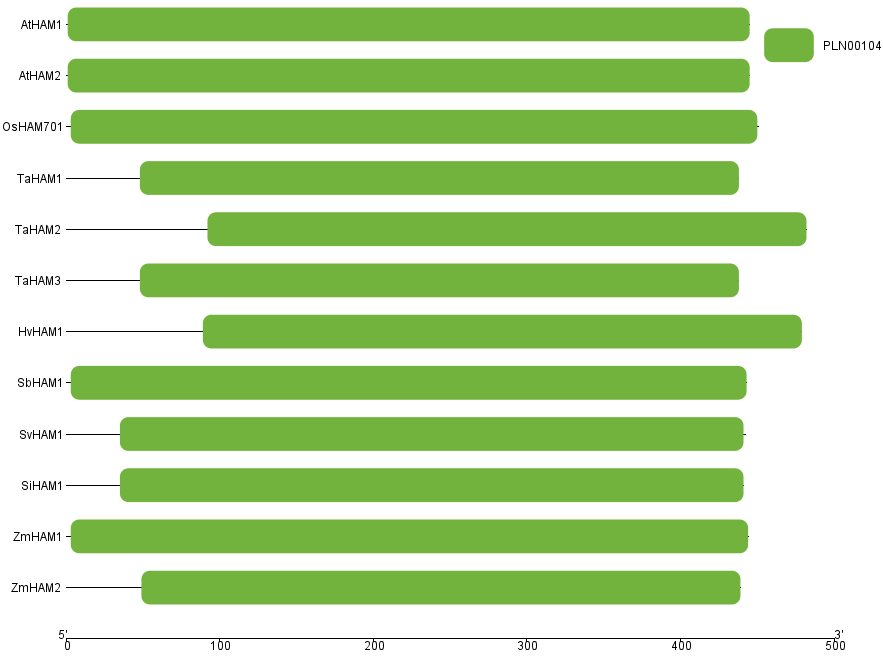


Figure S2-19 Conserved domain analysis of HAC proteins.


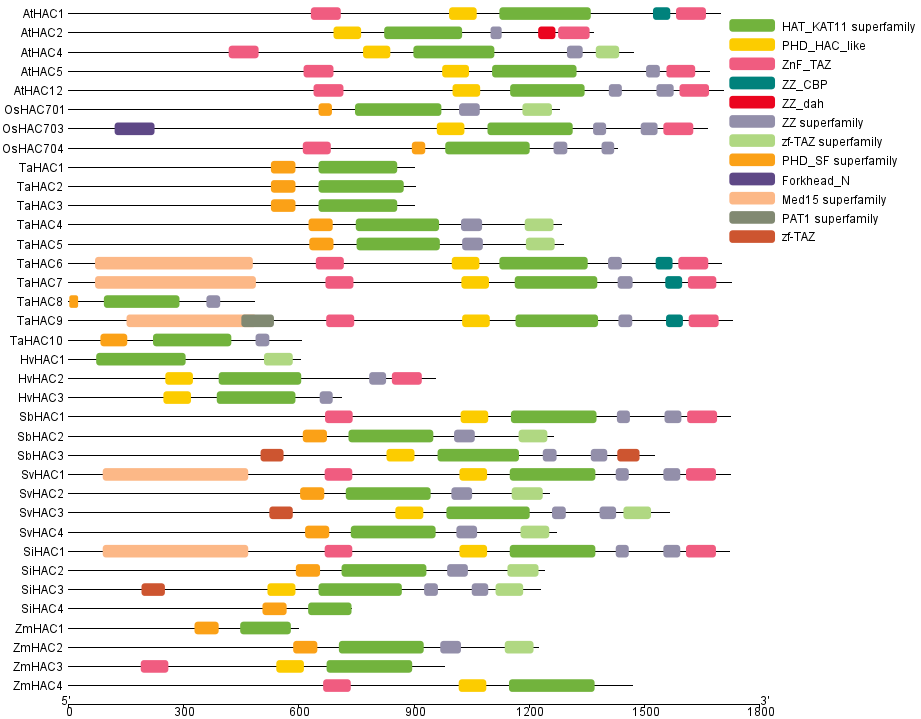


Figure S2-20 Conserved domain analysis of HAF proteins.


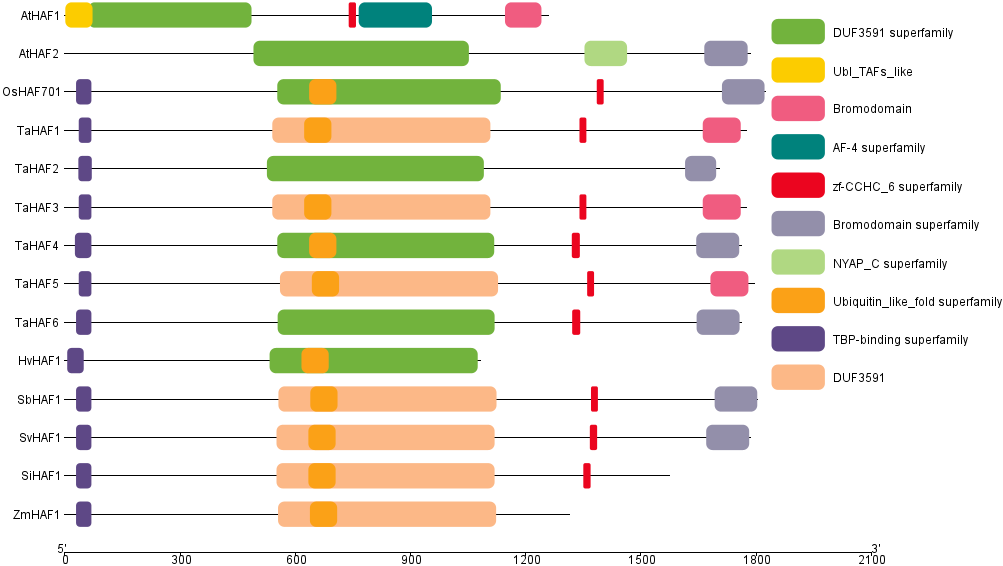


Figure S2-21 Conserved domain analysis of *Arabidopsis* and rice HDA proteins.


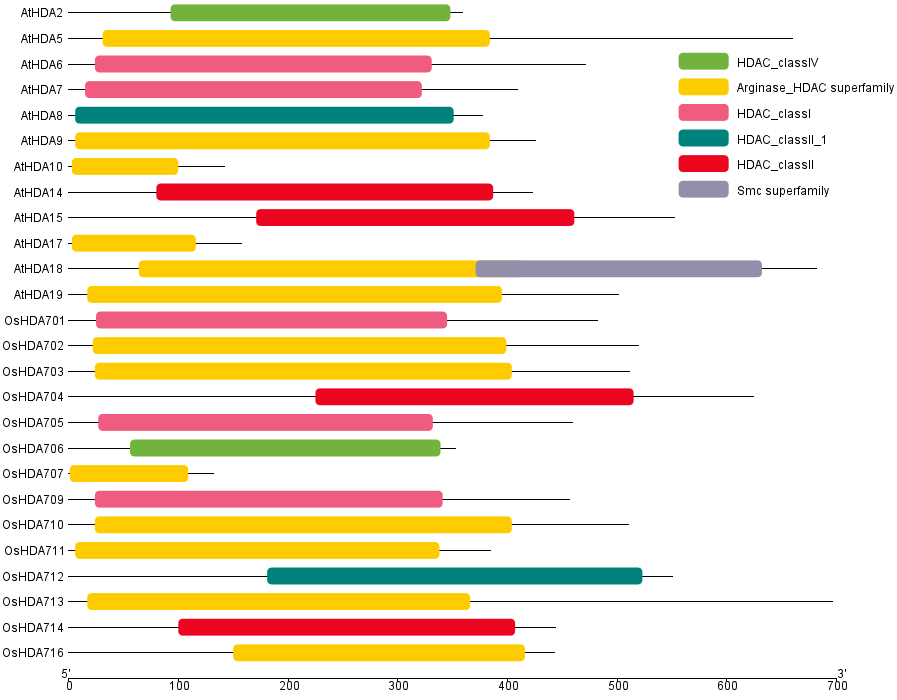


Figure S2-22 Conserved domain analysis of TaHDA proteins.


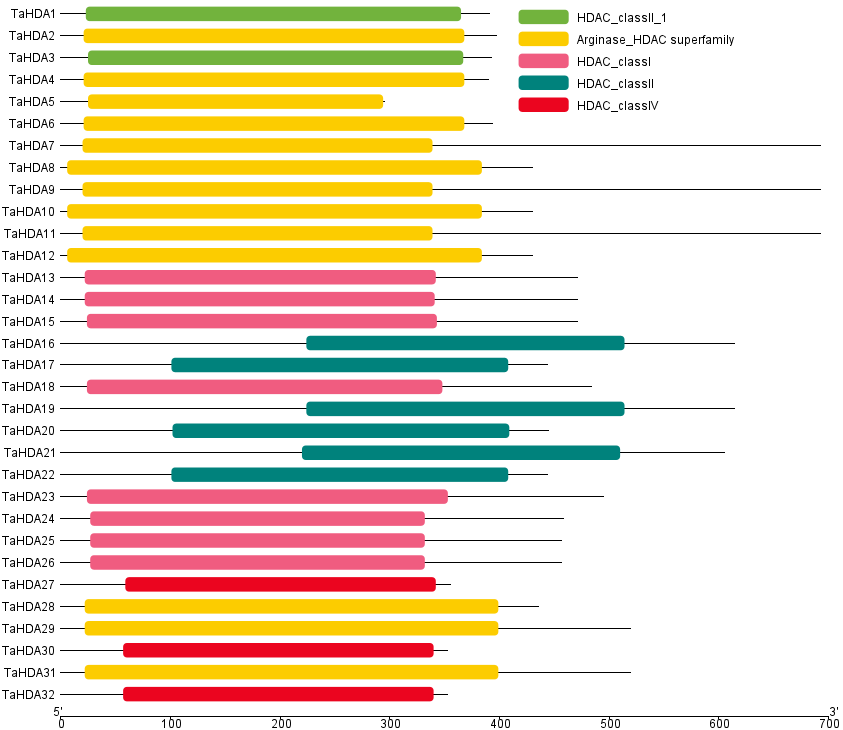


Figure S2-23 Conserved domain analysis of HvHDA proteins.


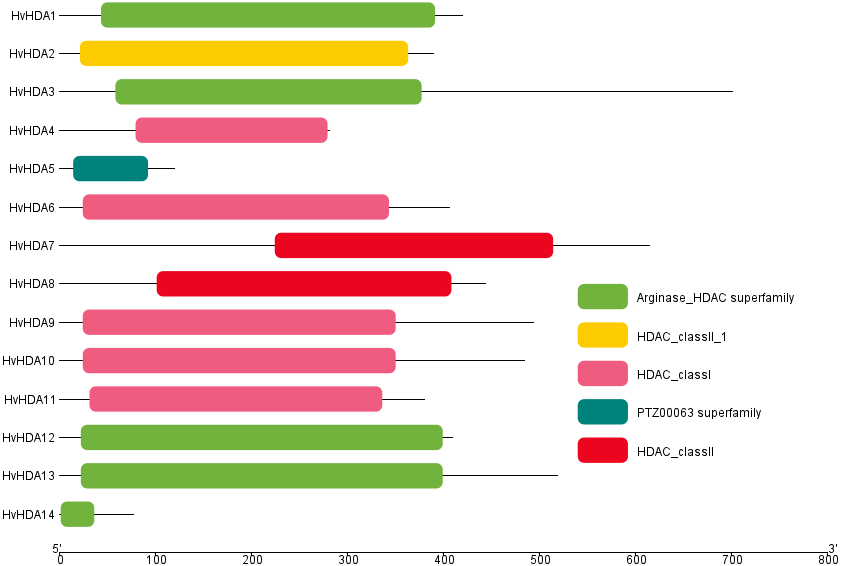


Figure S2-24 Conserved domain analysis of SbHDA proteins.


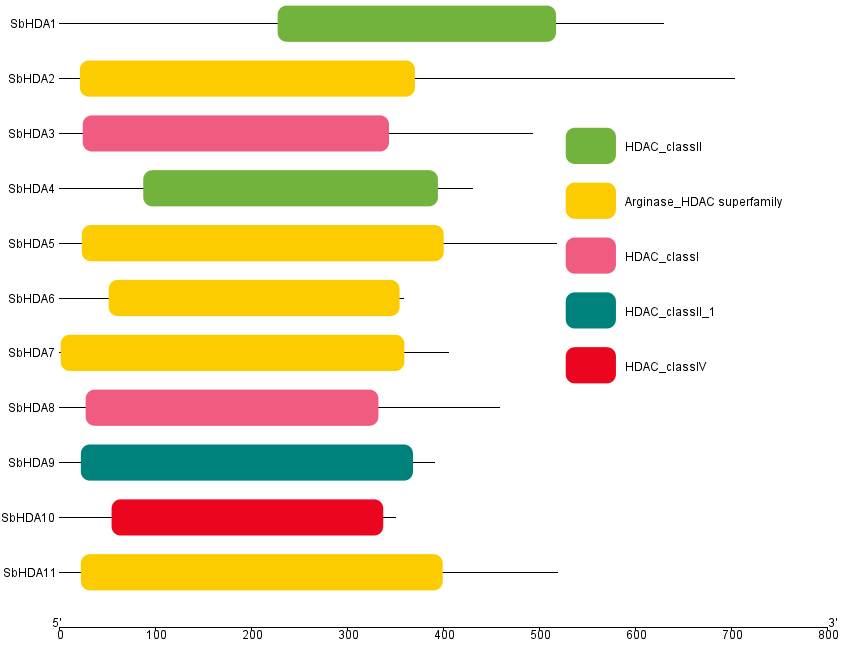


Figure S2-25 Conserved domain analysis of SvHDA proteins.


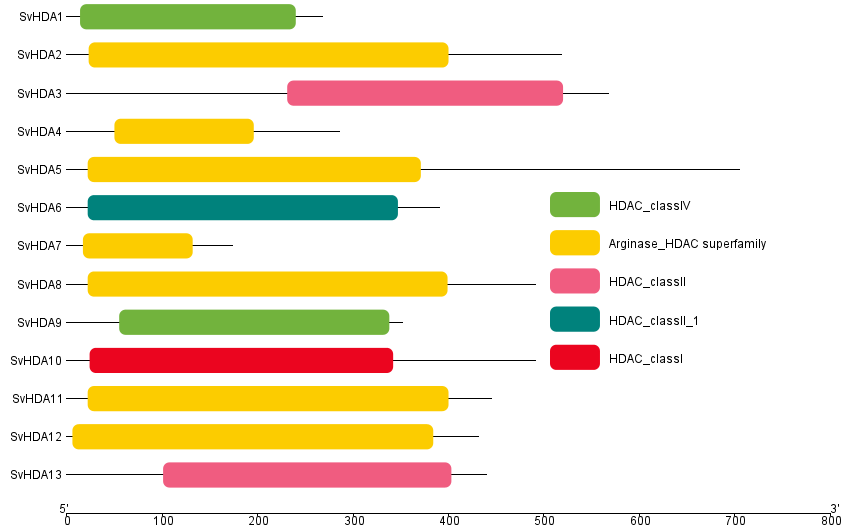


Figure S2-26 Conserved domain analysis of SiHDA proteins.


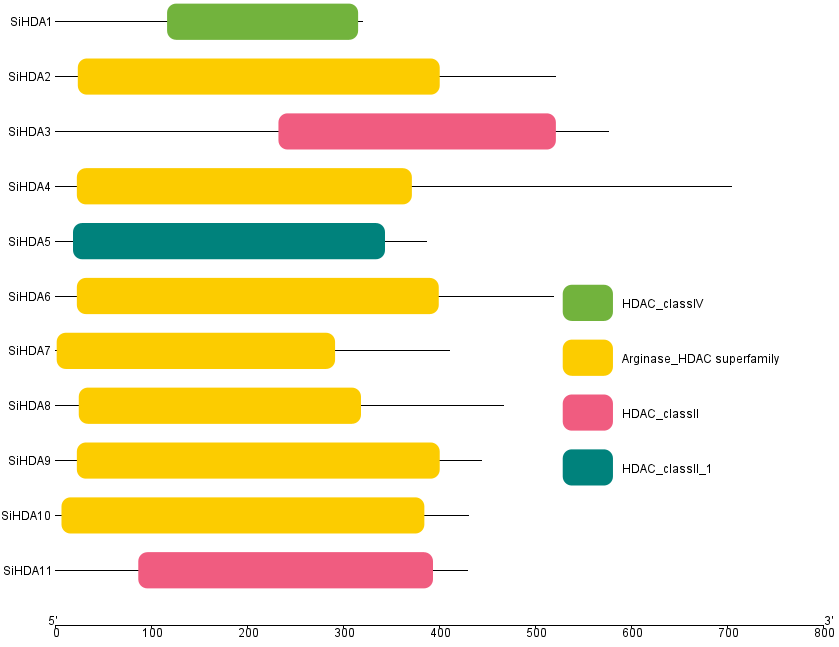


Figure S2-27 Conserved domain analysis of ZmHDA proteins.


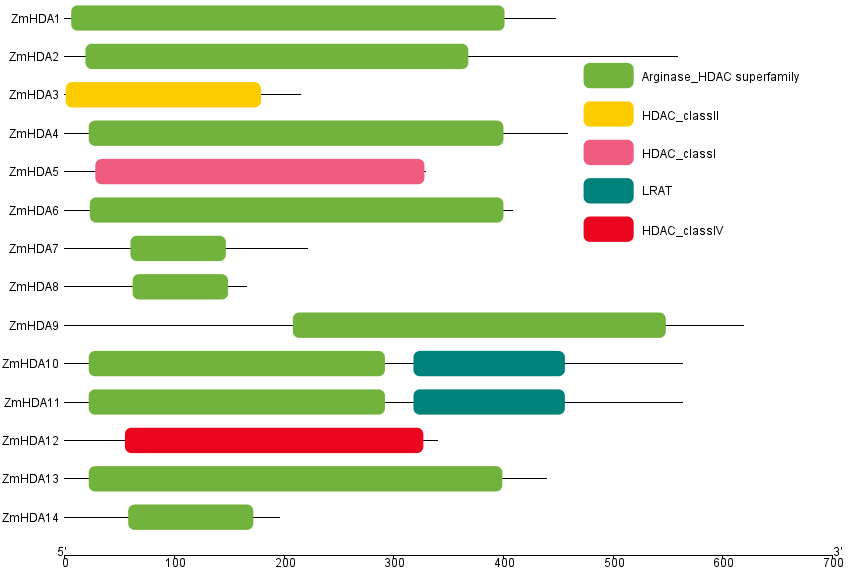


Figure S2-28 Conserved domain analysis of SRT proteins.


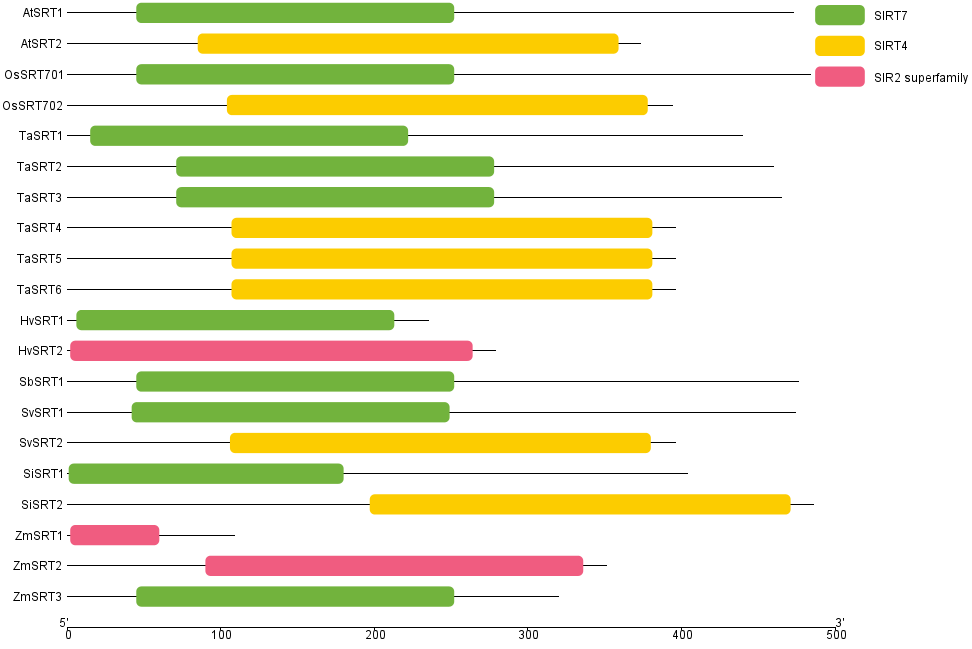


Figure S2-29 Conserved domain analysis of HDT proteins.


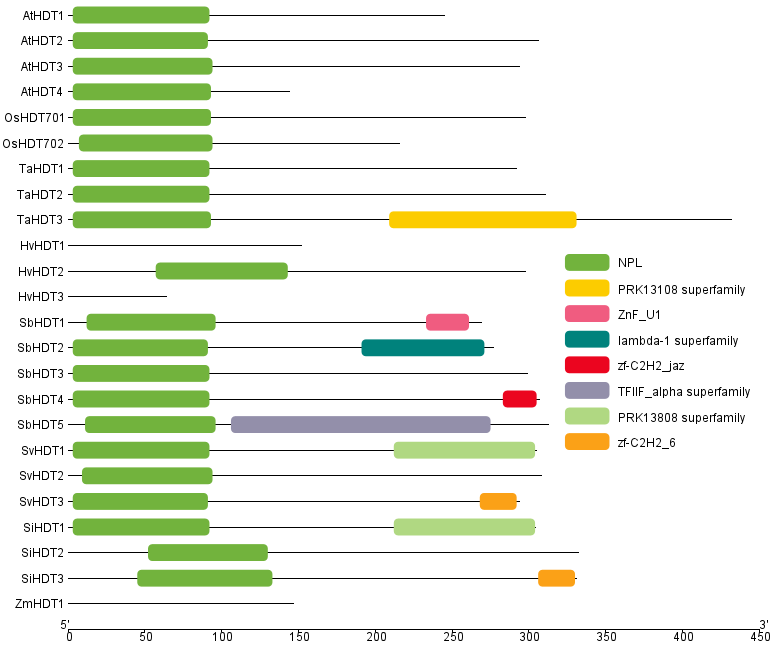

Supplement: Supplementary file 2 — Additional file 2: Figure S2. Conserved domain analysis of HM proteins. [file 12870_2021_3332_MOESM2_ESM.docx]
