# Supplementary material for: Genome-wide identification of Gramineae histone modification genes and their potential roles in regulating wheat and maize growth and stress responses
Source: BMC Plant Biol. 2021 Nov 20;21:543. doi: 10.1186/s12870-021-03332-8 (PMC8605605; doi:10.1186/s12870-021-03332-8)

**Figure S3 Phylogenetic analysis of *HM* genes.**

Figure S3-1 Phylogenetic analysis of *T. aestivum*, *Arabidopsis*, and rice *SDG* and *PRMT* genes.


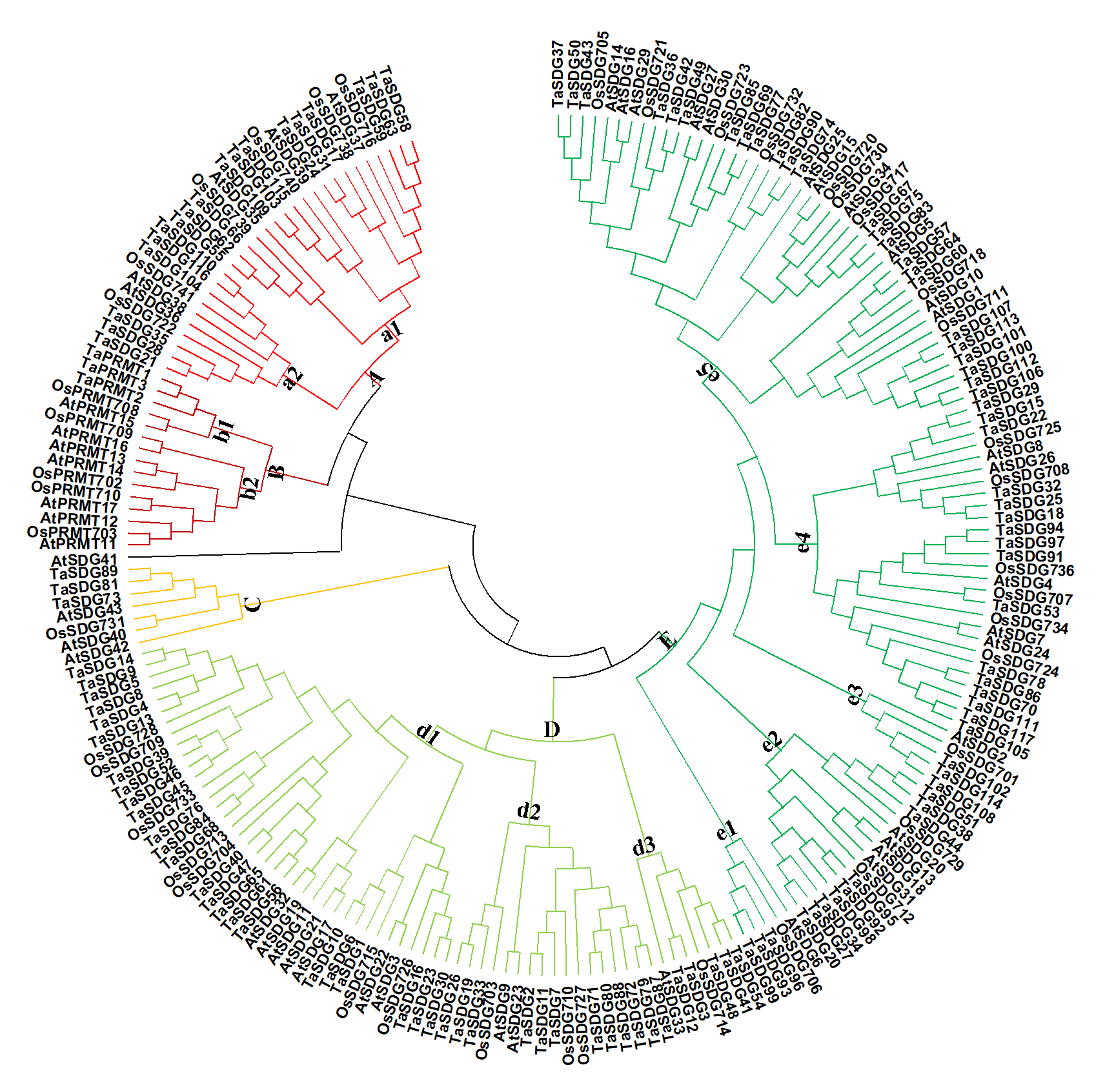


Figure S3-2 Phylogenetic analysis of *H. vulgare*, *Arabidopsis,* and rice *SDG* and *PRMT* genes.


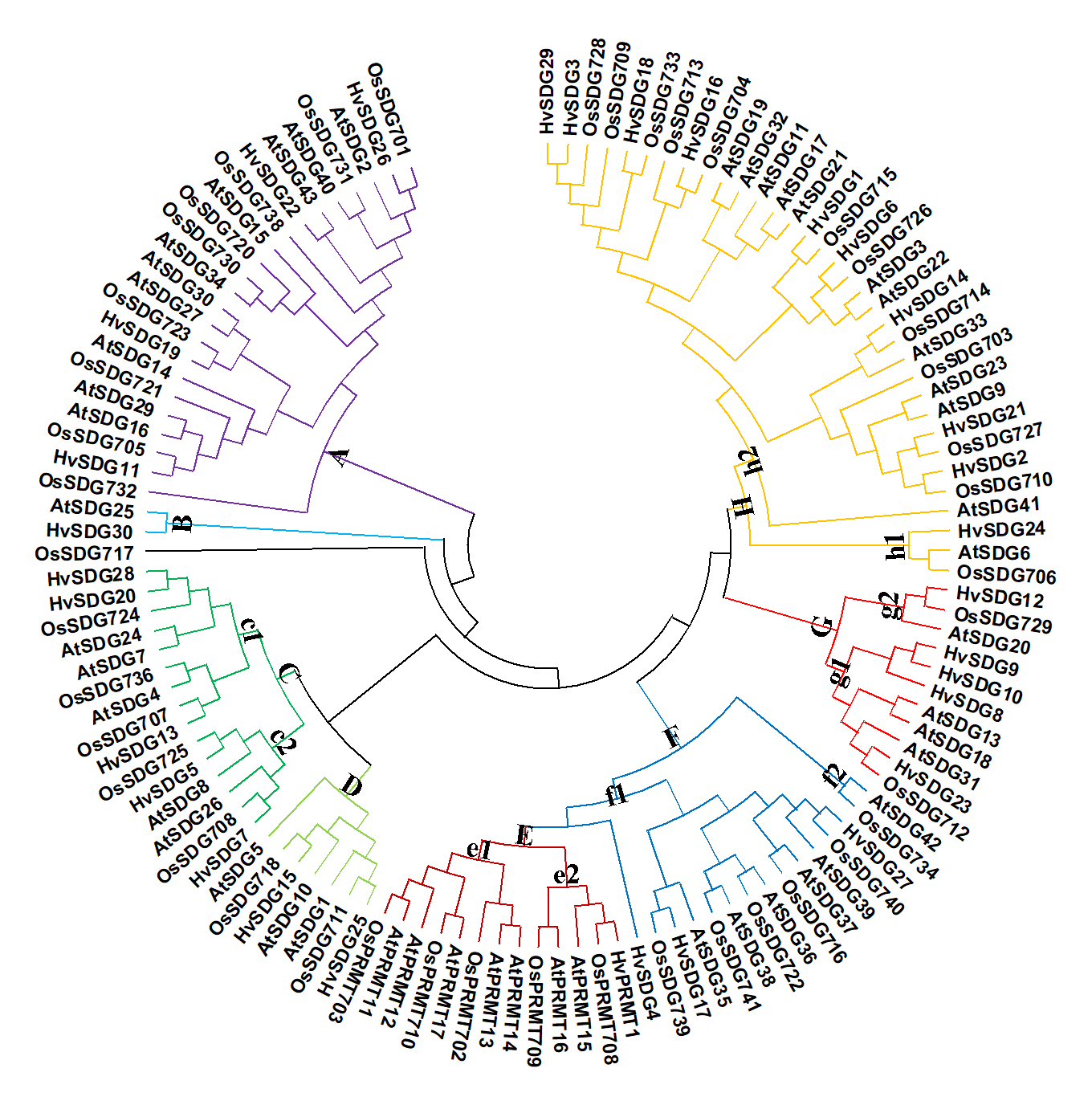


Figure S3-3 Phylogenetic analysis of *S. bicolor*, *Arabidopsis*, and rice *SDG* and *PRMT* genes.


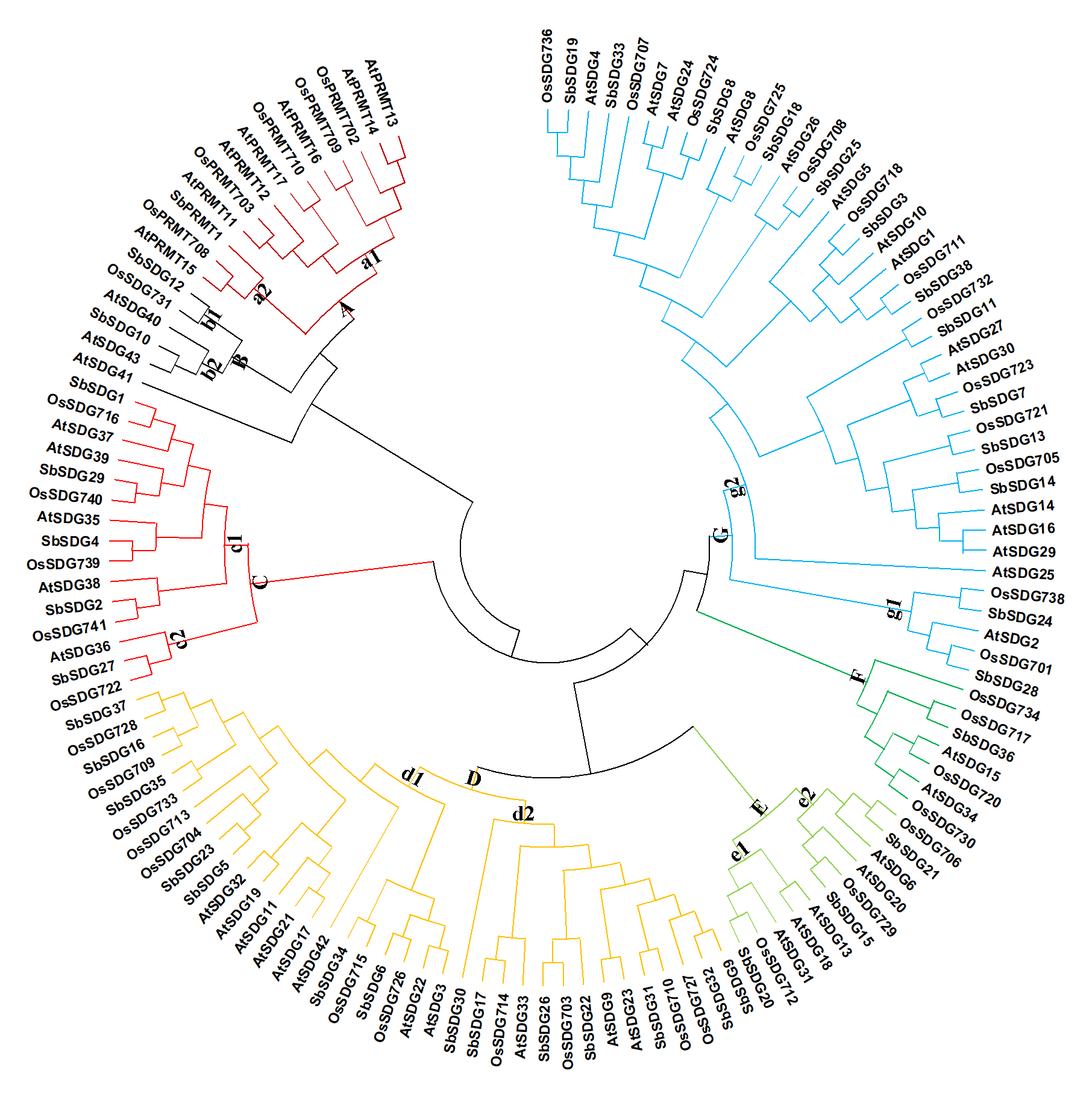


Figure S3-4 Phylogenetic analysis of *S. viridis*, *Arabidopsis*, and rice *SDG* and *PRMT* genes.


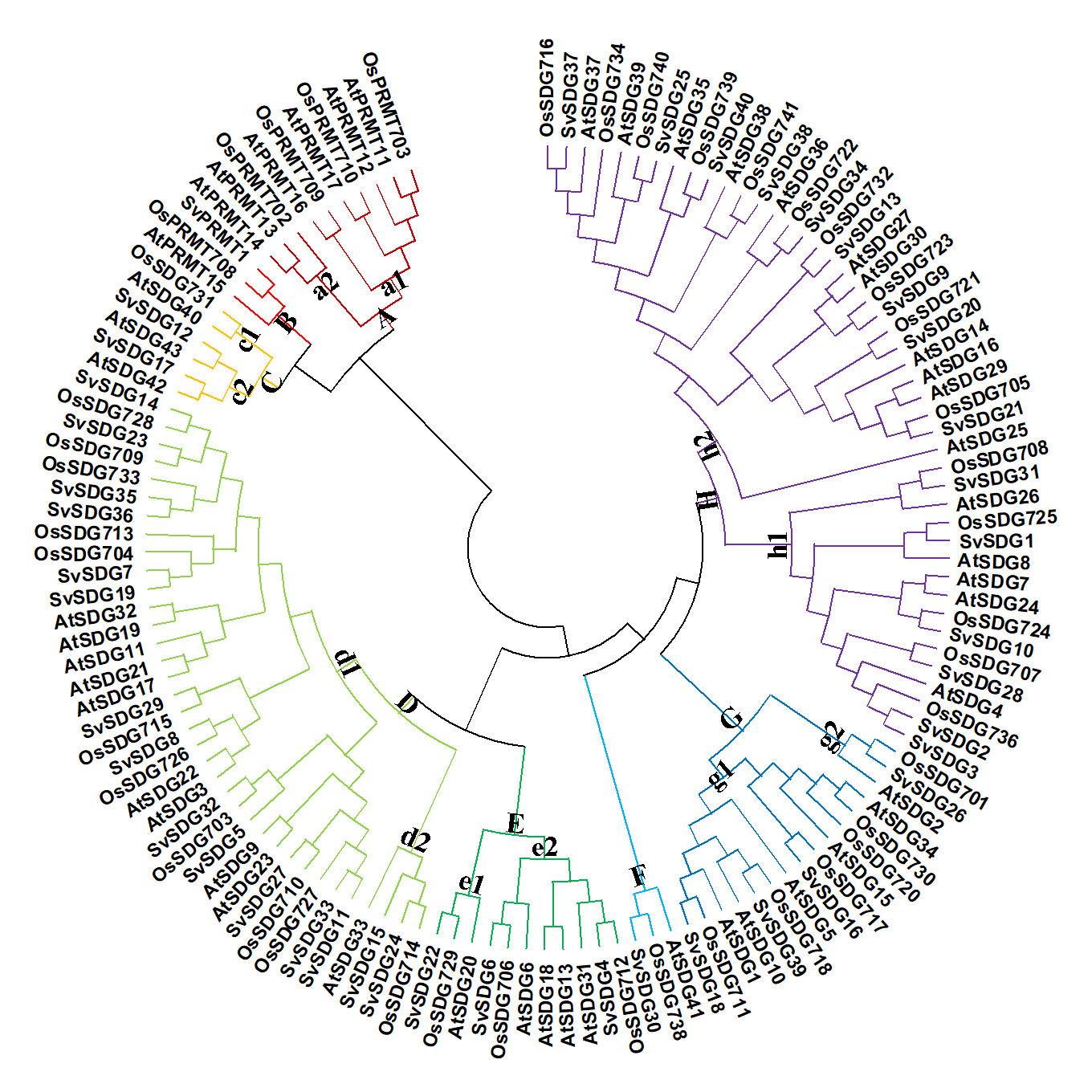


Figure S3-5 Phylogenetic analysis of *S. italica*, *Arabidopsis*, and rice *SDG* and *PRMT* genes.


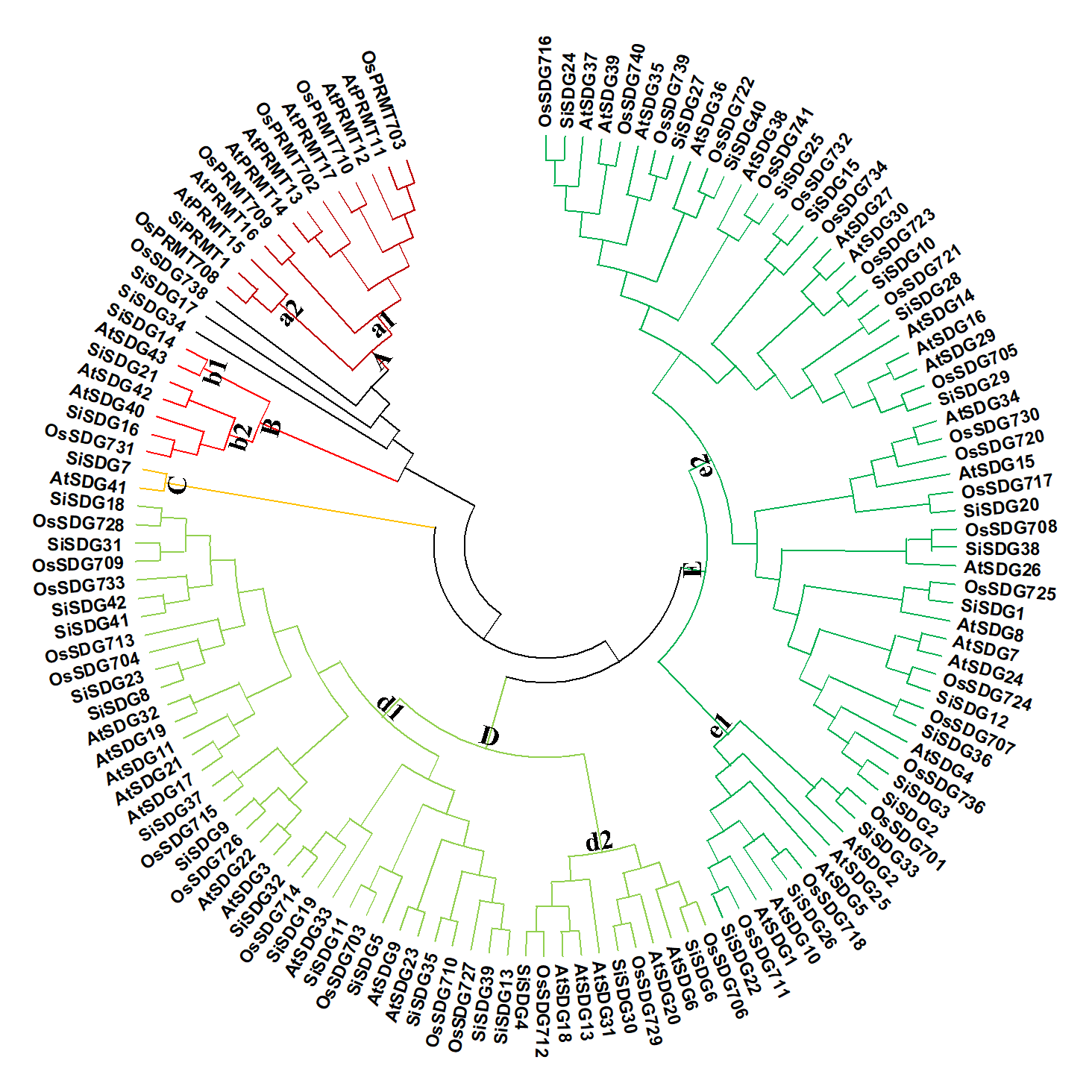


Figure S3-6 Phylogenetic analysis of *Z. mays*, *Arabidopsis*, and rice *SDG* and *PRMT* genes.


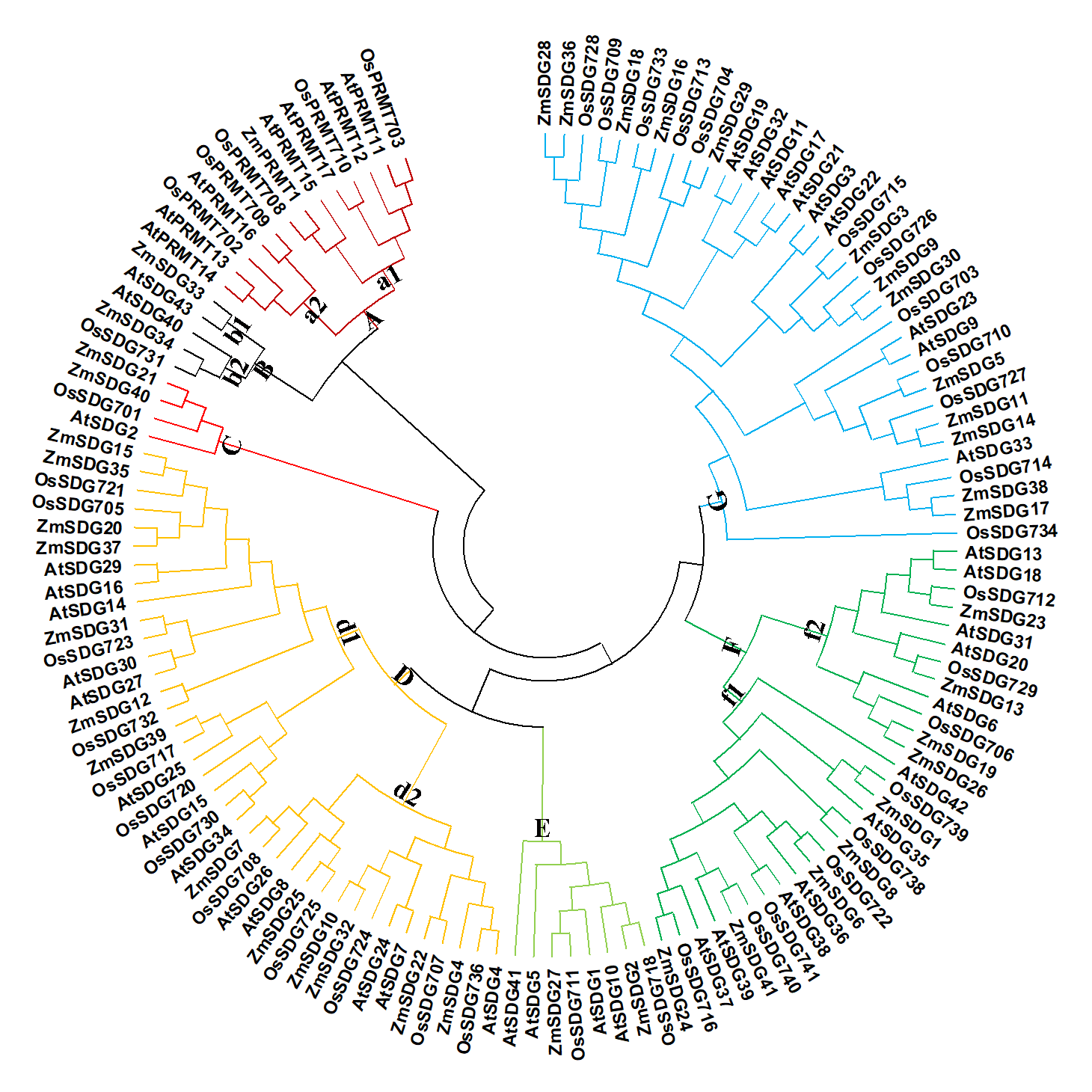


Figure S3-7 Phylogenetic analysis of *HDMA* and *JMJ* genes.


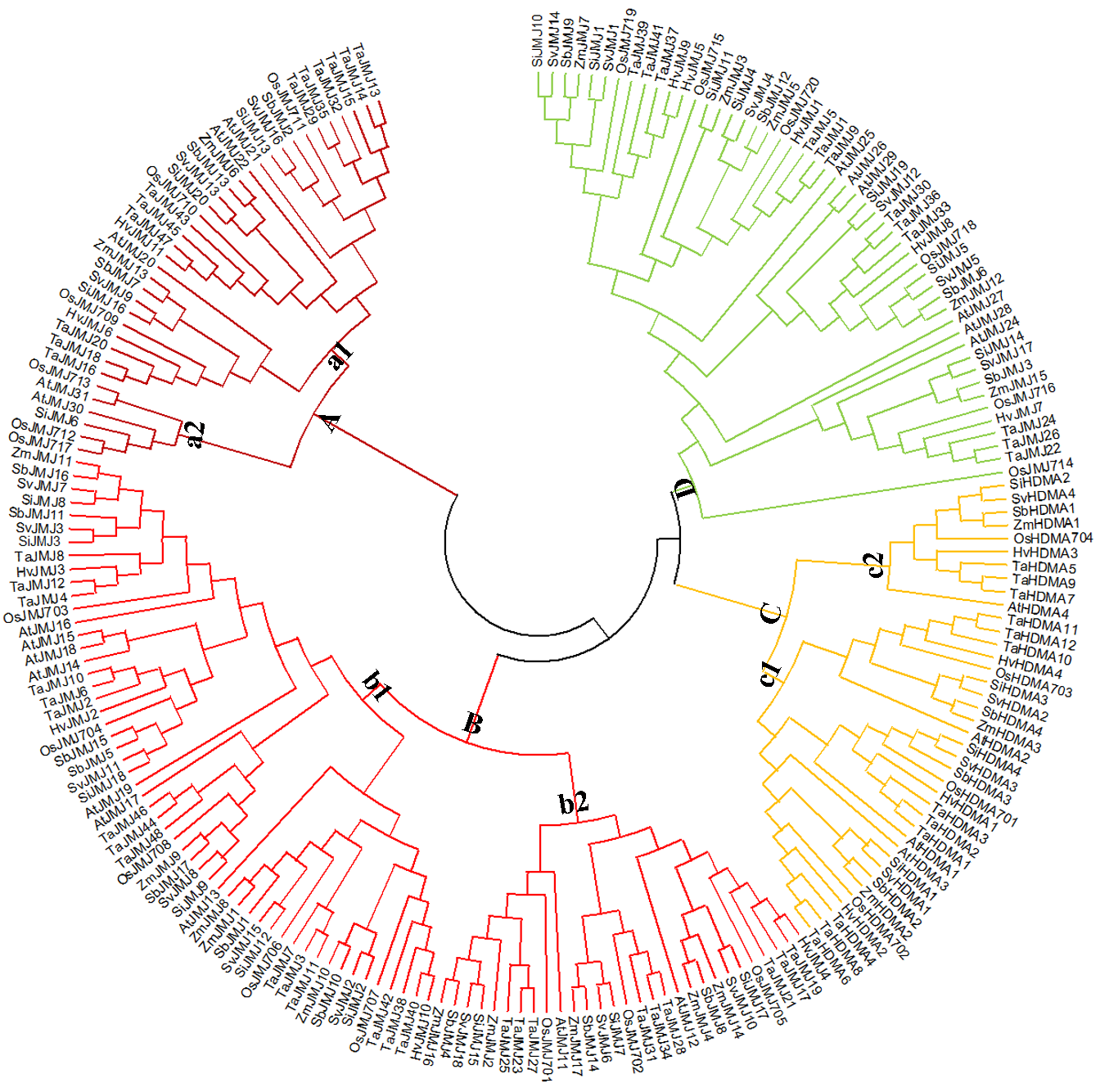


Figure S3-8 Phylogenetic analysis of *HAG*, *HAM*, *HAC*, and *HAF* genes.


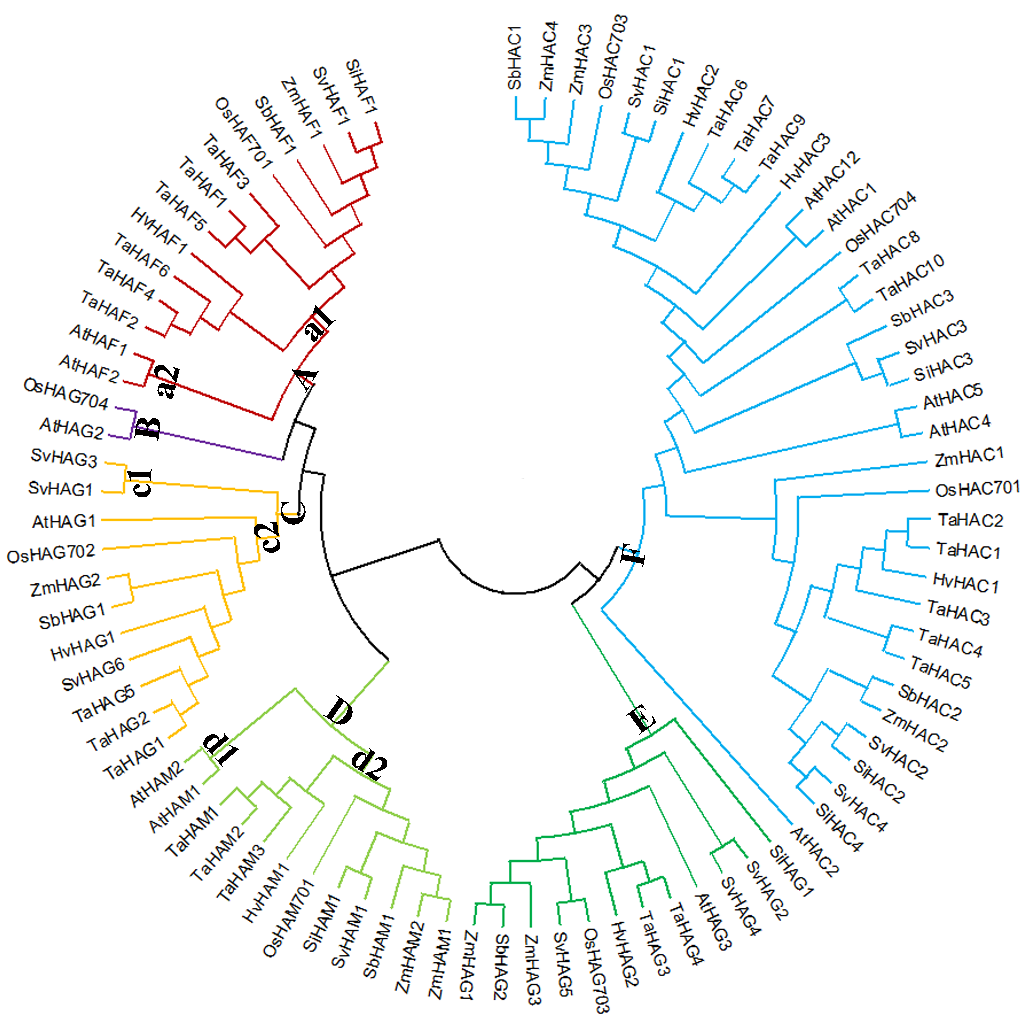


Figure S3-9 Phylogenetic analysis of *HDA*, *SRT*, and *HDT* genes


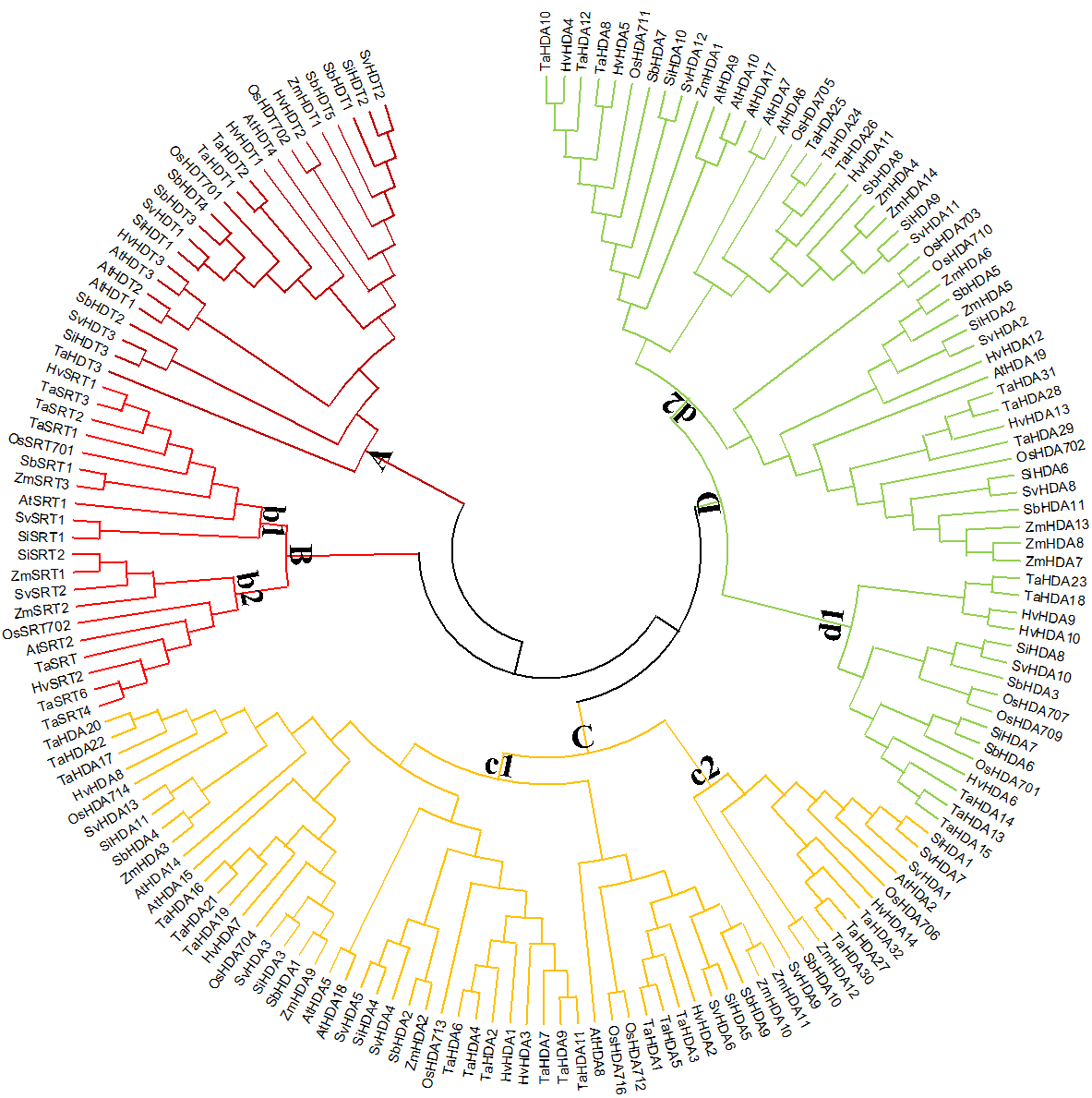

Supplement: Supplementary file 3 — Additional file 3: Figure S3. Phylogenetic analysis of HM genes. [file 12870_2021_3332_MOESM3_ESM.docx]
