# Supplementary material for: Genome-wide identification of Gramineae histone modification genes and their potential roles in regulating wheat and maize growth and stress responses
Source: BMC Plant Biol. 2021 Nov 20;21:543. doi: 10.1186/s12870-021-03332-8 (PMC8605605; doi:10.1186/s12870-021-03332-8)

**Figure S4 Synteny analysis of each Gramineae *HM* gene.**

Figure S4-1 Synteny analysis of *TaHM* genes.


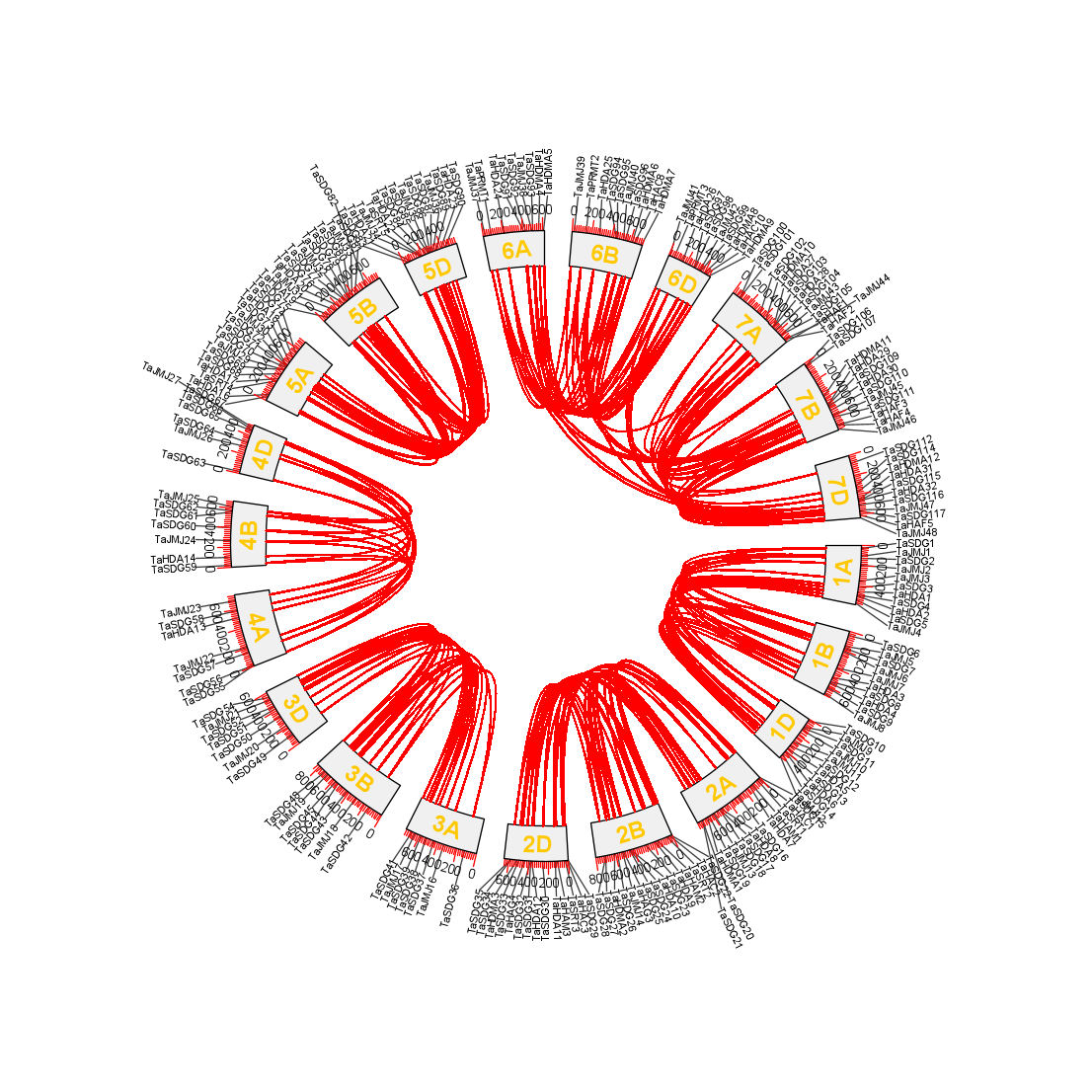


Figure S4-2 Synteny analysis of *SbHM* genes.


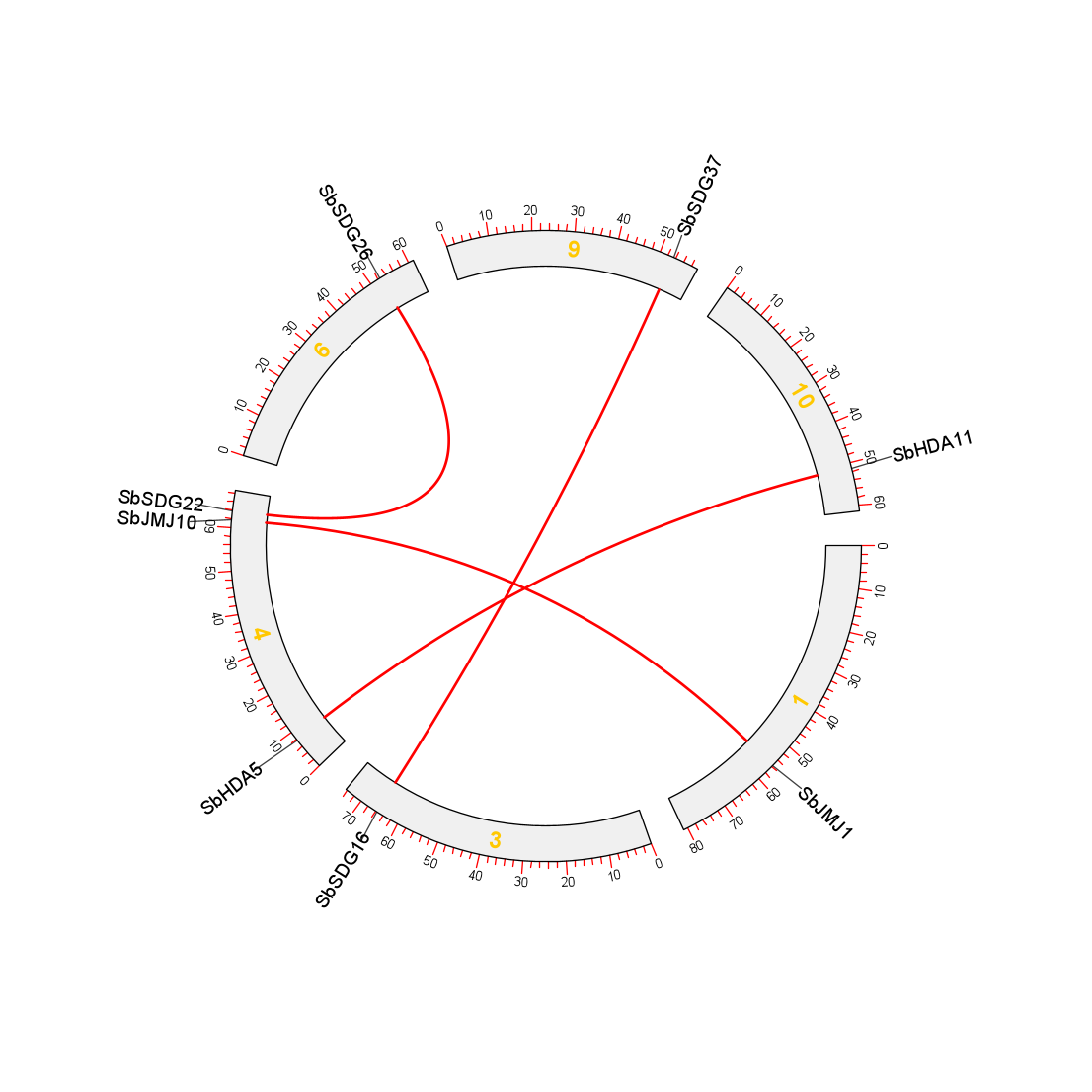


Figure S4-3 Synteny analysis of *SvHM* genes.


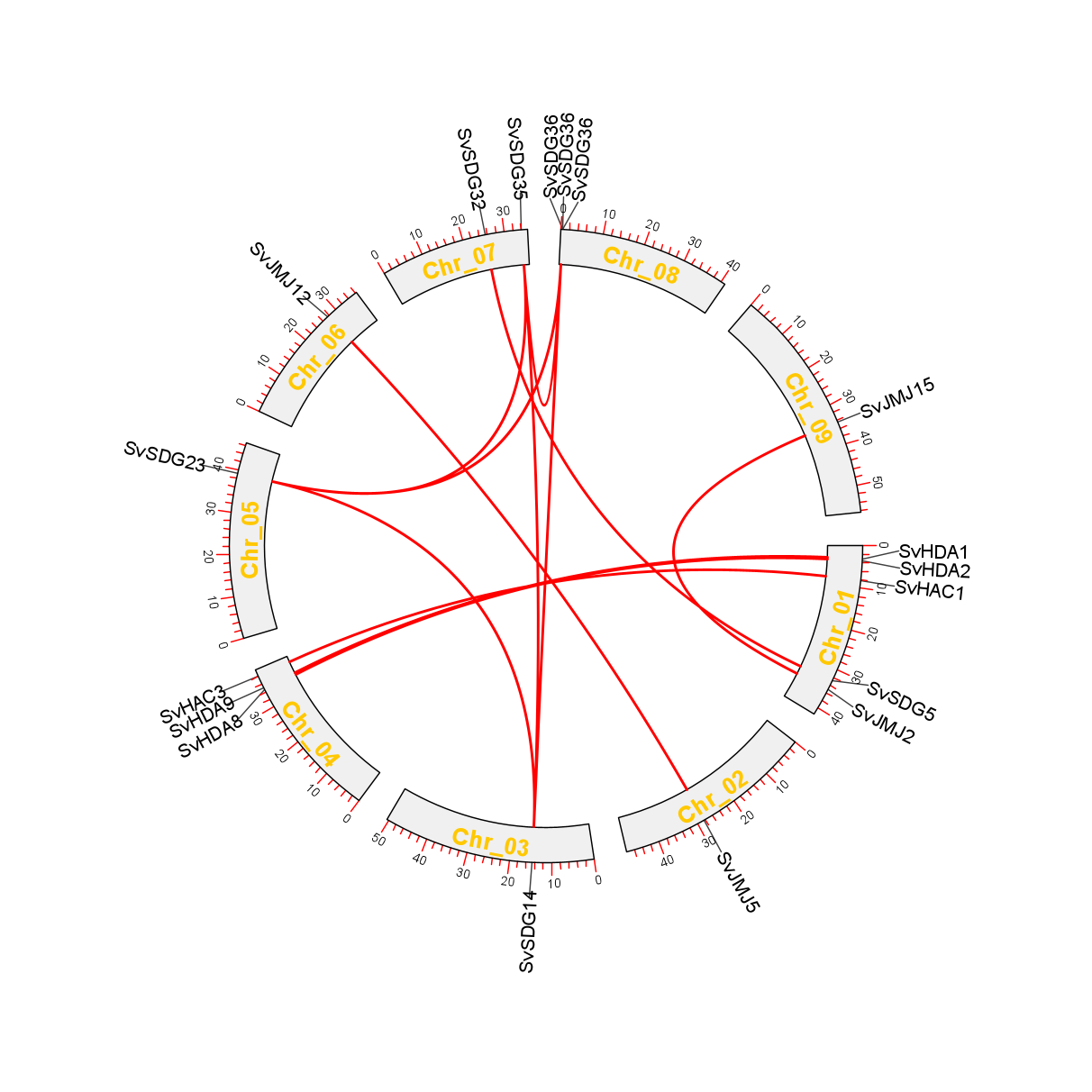


Figure S4-4 Synteny analysis of *SiHM* genes.


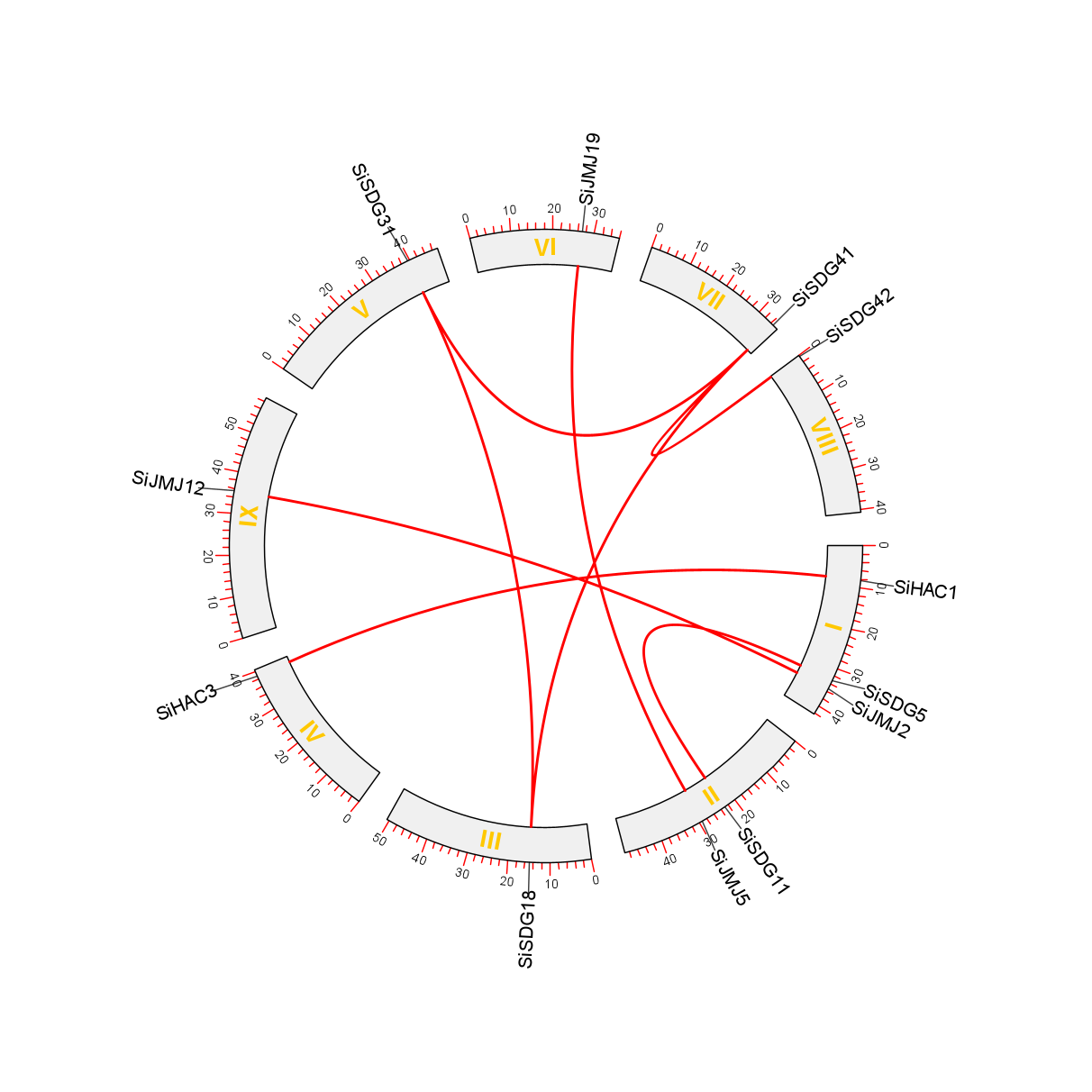


Figure S4-5 Synteny analysis of *ZmHM* genes.


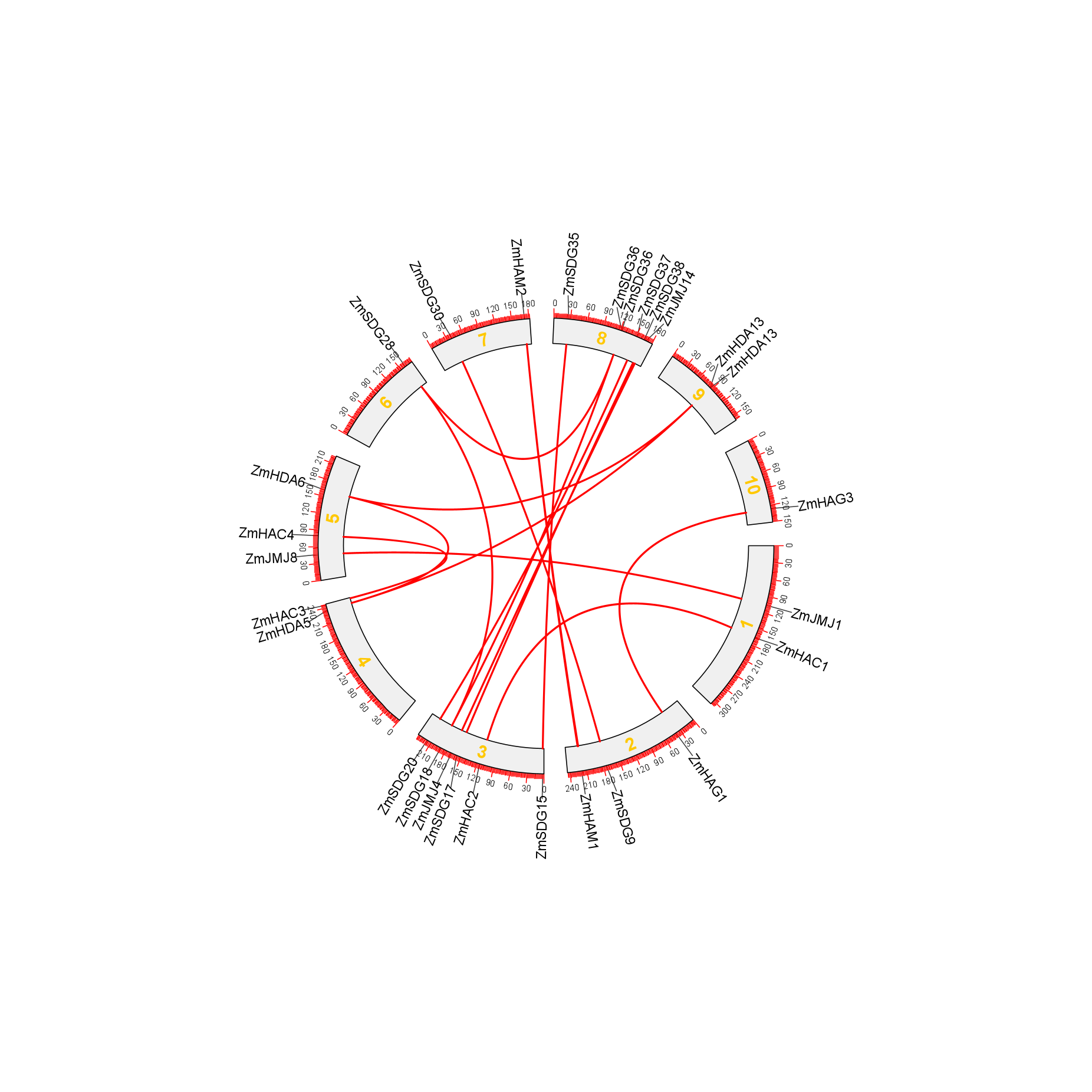

Supplement: Supplementary file 4 — Additional file 4: Figure S4. Synteny analysis of each Gramineae HM gene. [file 12870_2021_3332_MOESM4_ESM.docx]
