# Supplementary material for: Genome-wide identification of Gramineae histone modification genes and their potential roles in regulating wheat and maize growth and stress responses
Source: BMC Plant Biol. 2021 Nov 20;21:543. doi: 10.1186/s12870-021-03332-8 (PMC8605605; doi:10.1186/s12870-021-03332-8)

**Figure S5 Synteny analysis of *HM* genes between each Gramineae species and *Arabidopsis.***

Figure S5-1 Synteny analysis of *HM* genes between *T. aestivum* and *Arabidopsis.*


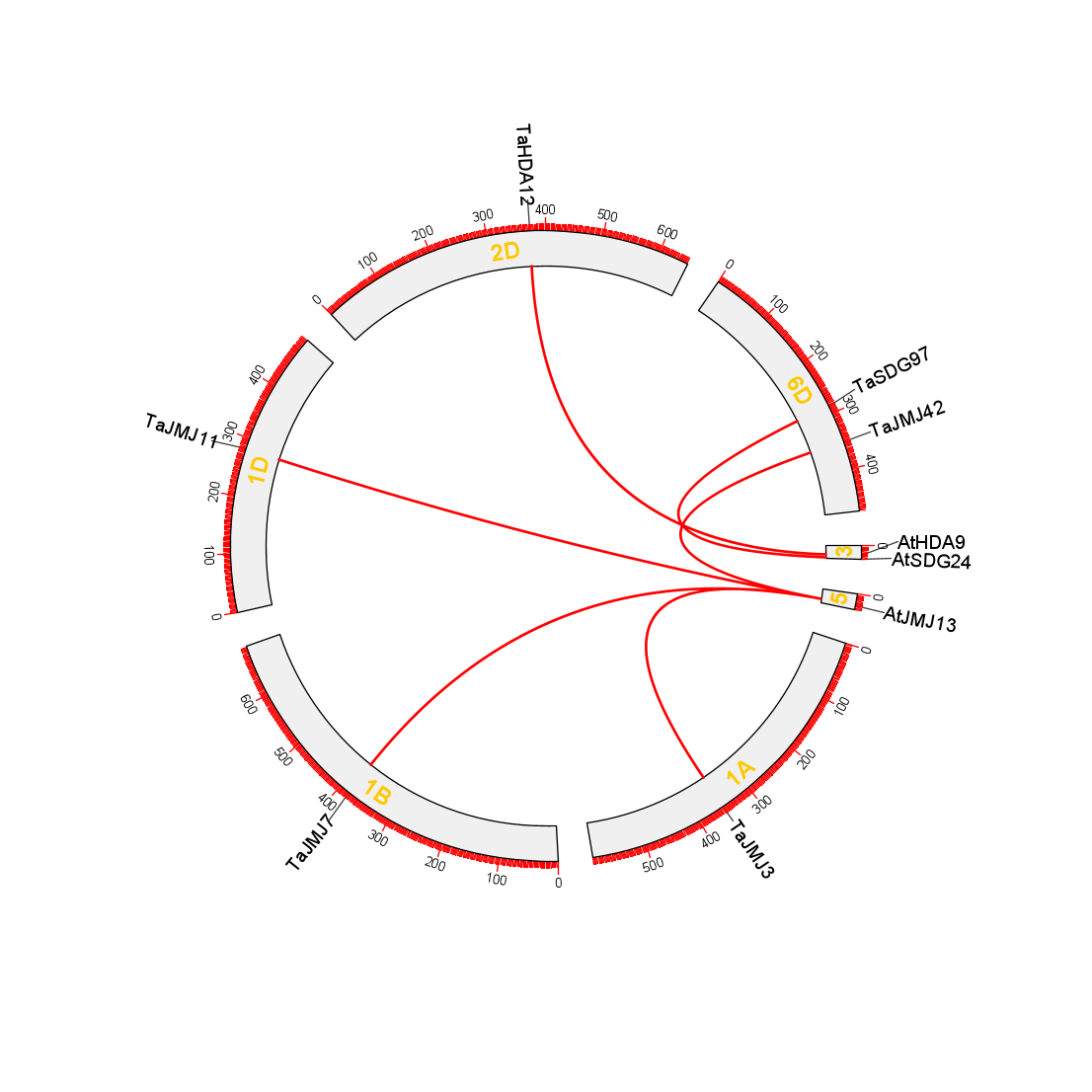


Figure S5-2 Synteny analysis of *HM* genes between *S. bicolor* and *Arabidopsis.*


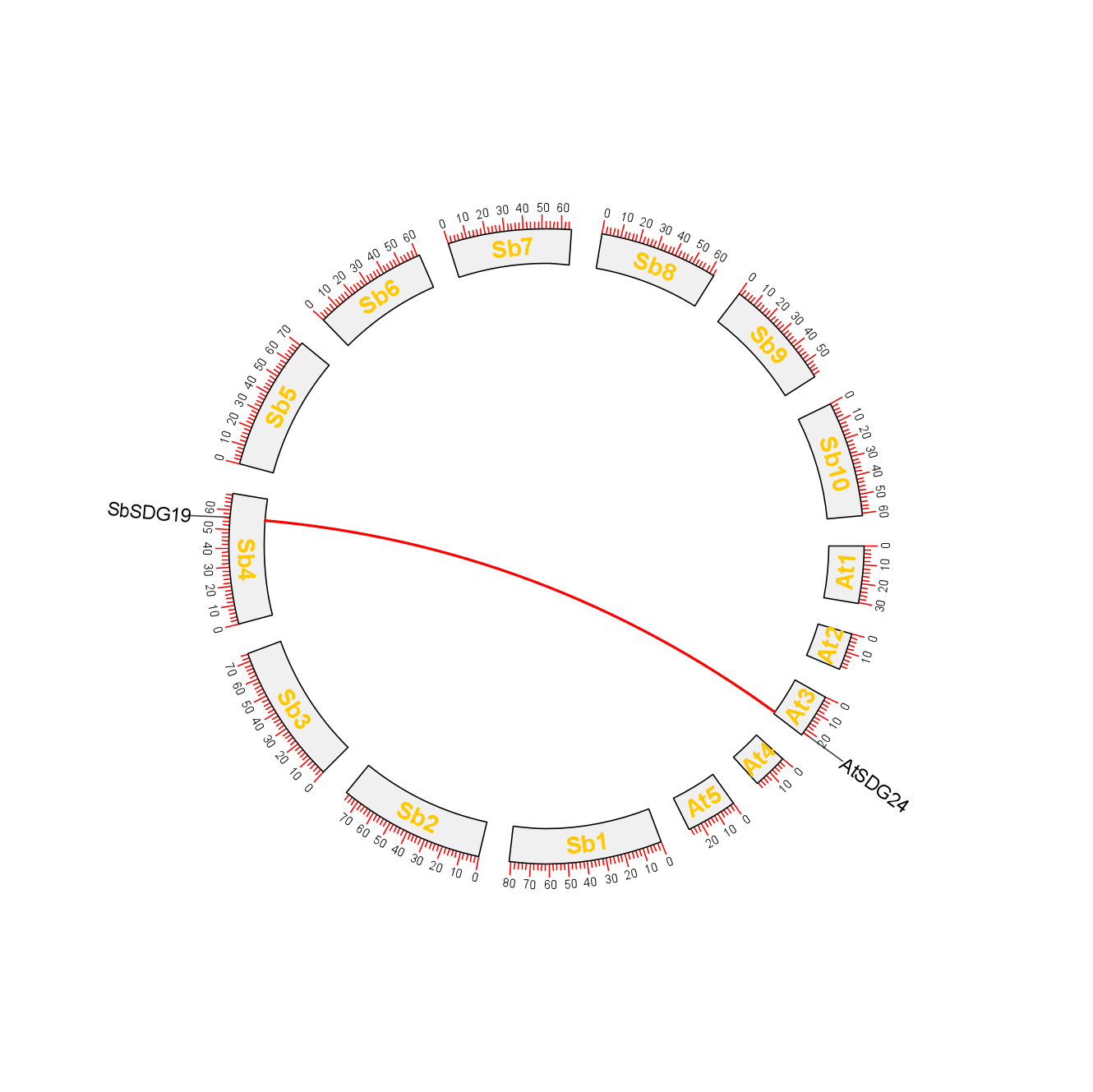


Figure S5-3 Synteny analysis of *HM* genes between *S. viridis* and *Arabidopsis.*


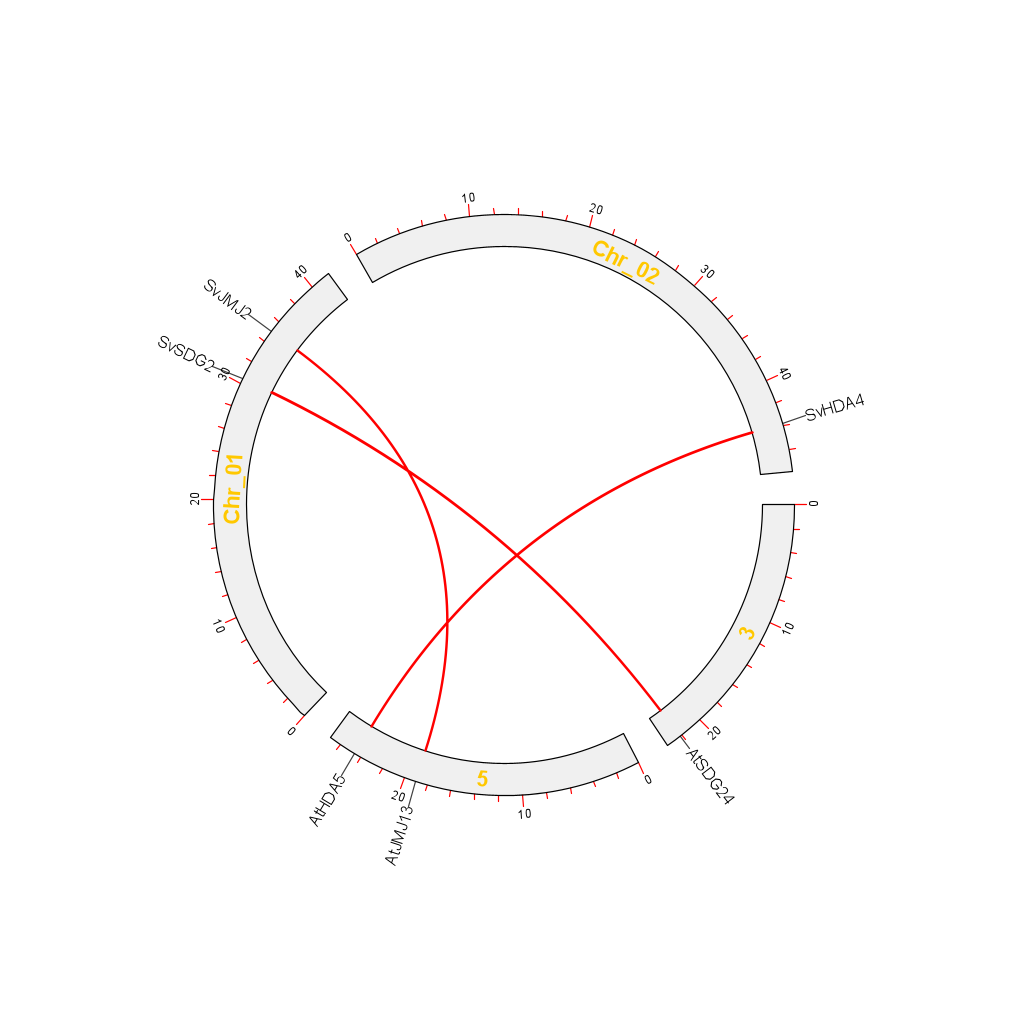


Figure S5-4 Synteny analysis of *HM* genes between *S. italica* and *Arabidopsis.*


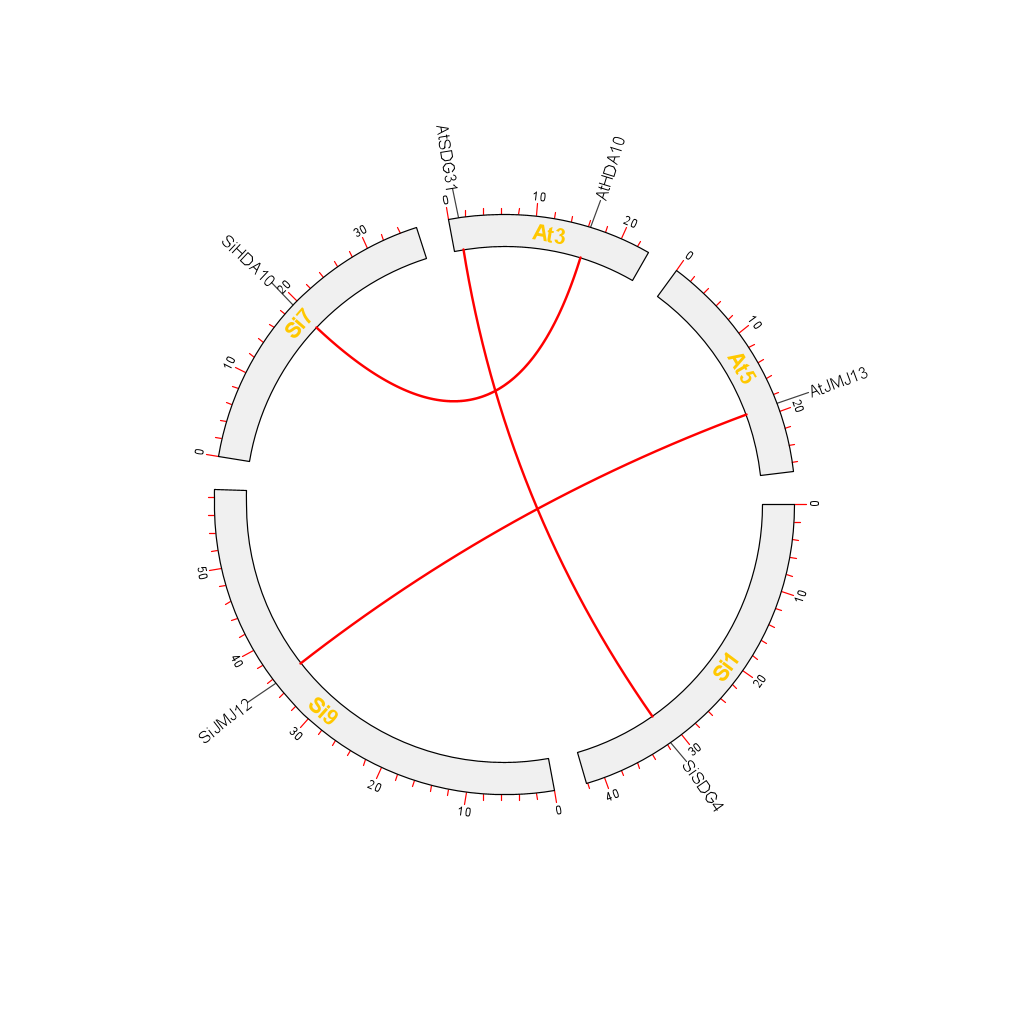

Supplement: Supplementary file 5 — Additional file 5: Figure S5. Synteny analysis of HM genes between each Gramineae species and Arabidopsis. [file 12870_2021_3332_MOESM5_ESM.docx]
