# Supplementary material for: Genome-wide identification of Gramineae histone modification genes and their potential roles in regulating wheat and maize growth and stress responses
Source: BMC Plant Biol. 2021 Nov 20;21:543. doi: 10.1186/s12870-021-03332-8 (PMC8605605; doi:10.1186/s12870-021-03332-8)

**Figure S6 Synteny analysis of *HM* genes between each Gramineae species and rice.**

Figure S6-1 Synteny analysis of *HM* genes between *T. aestivum* and rice.

*
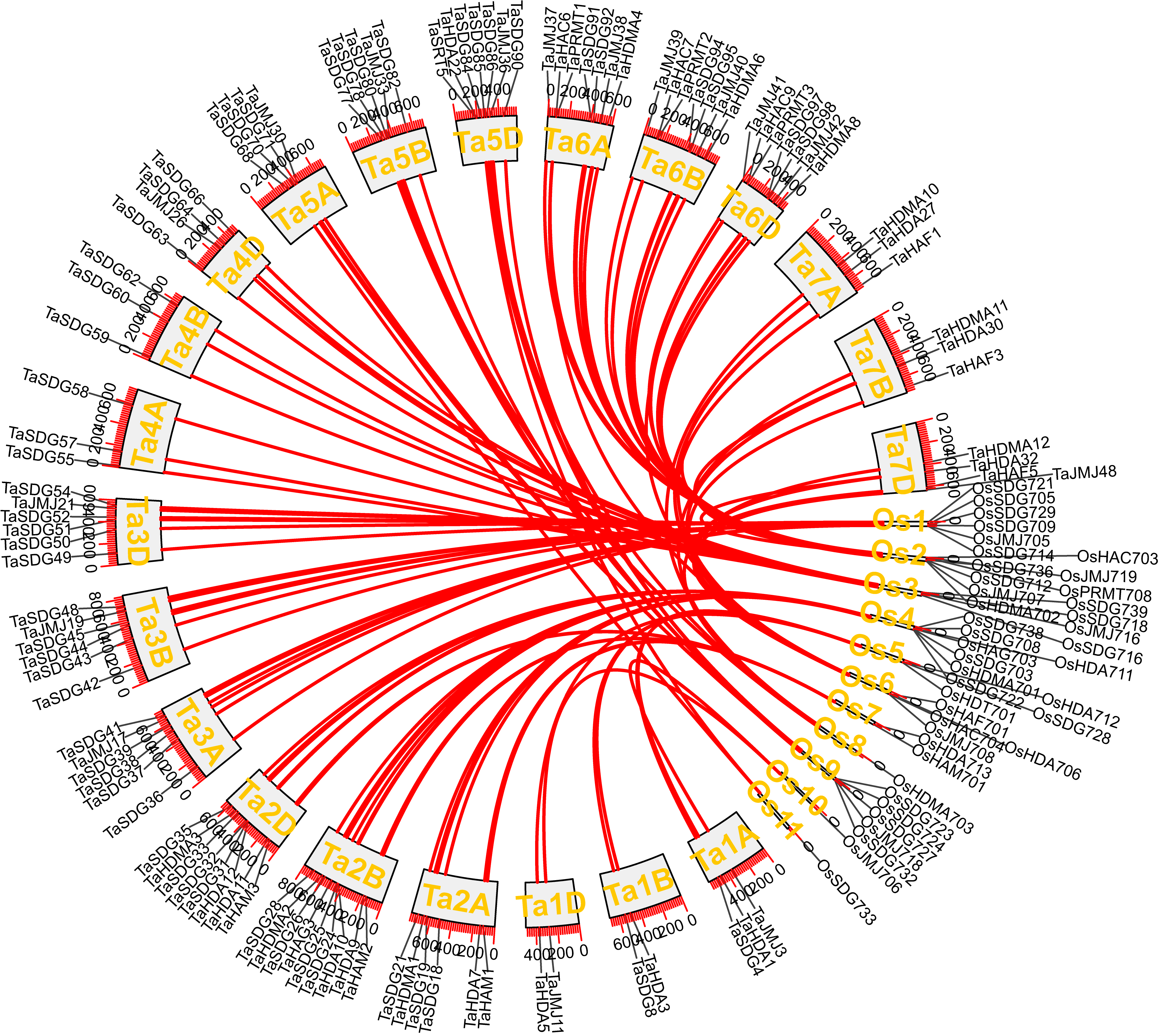
*

Figure S6-2 Synteny analysis of *HM* genes between *H. vulgare* and rice.


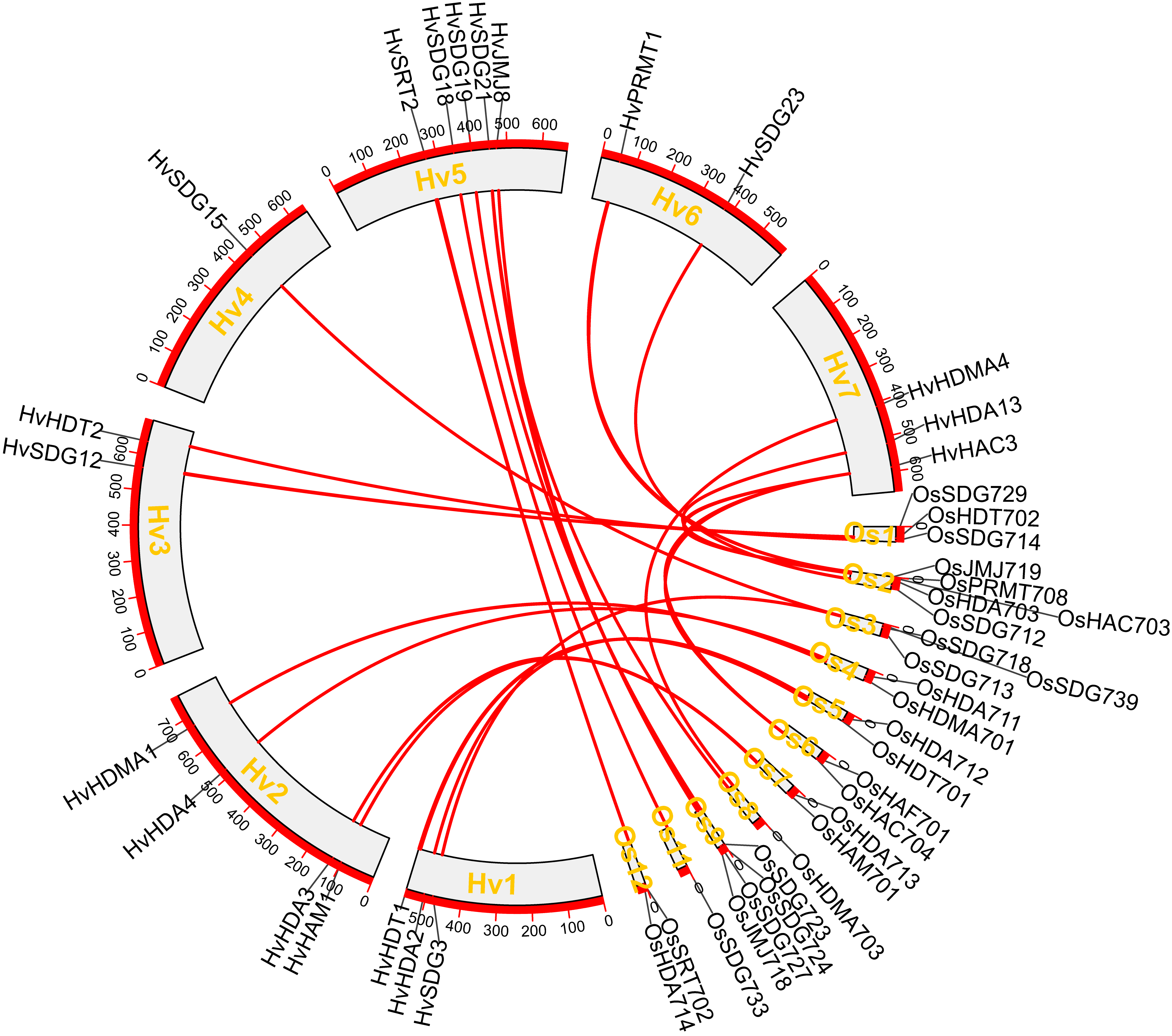


Figure S6-3 Synteny analysis of *HM* genes between *S. bicolor* and rice.


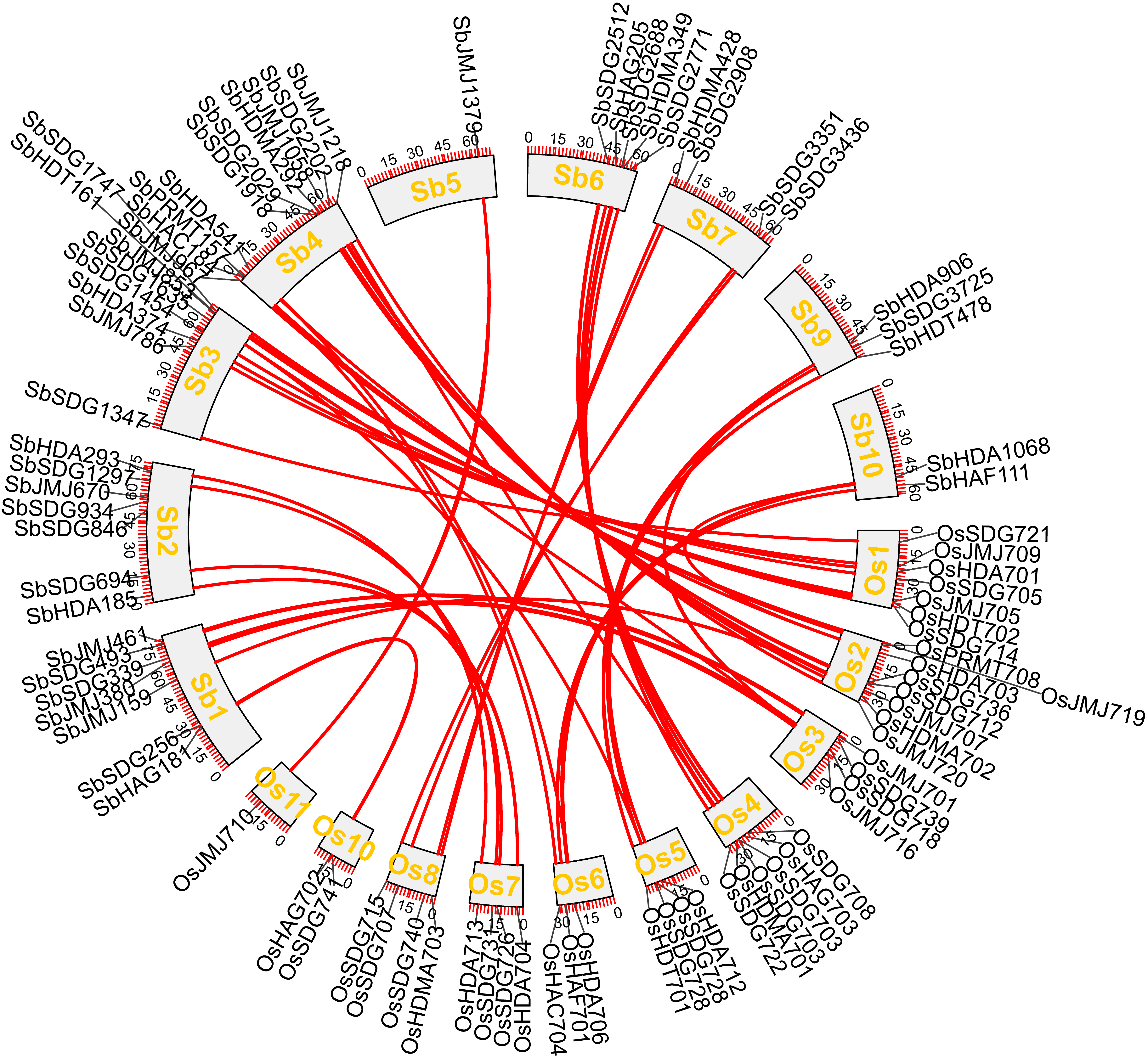


Figure S6-4 Synteny analysis of *HM* genes between *S. viridis* and rice.


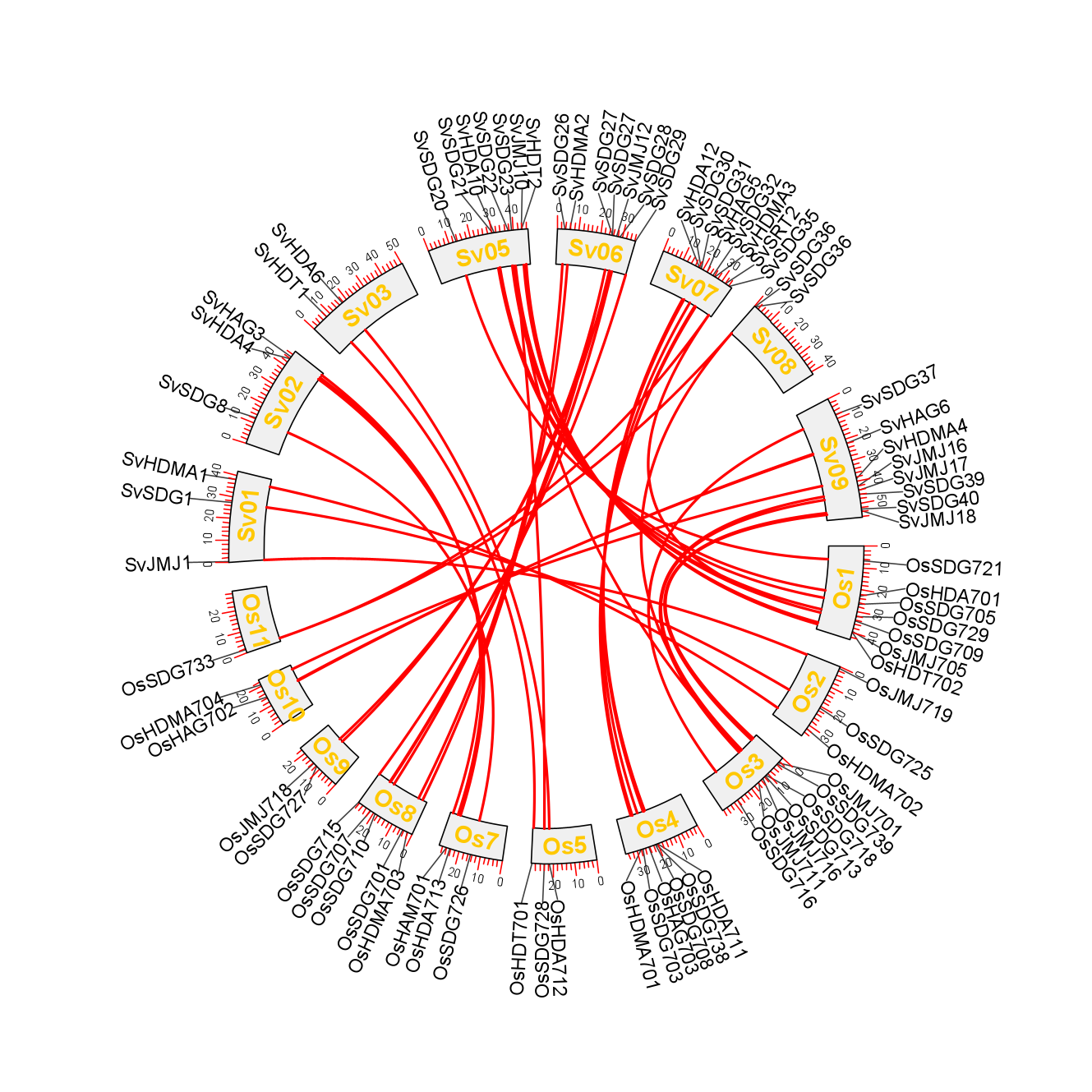


Figure S6-5 Synteny analysis of *HM* genes between *S. italica* and rice


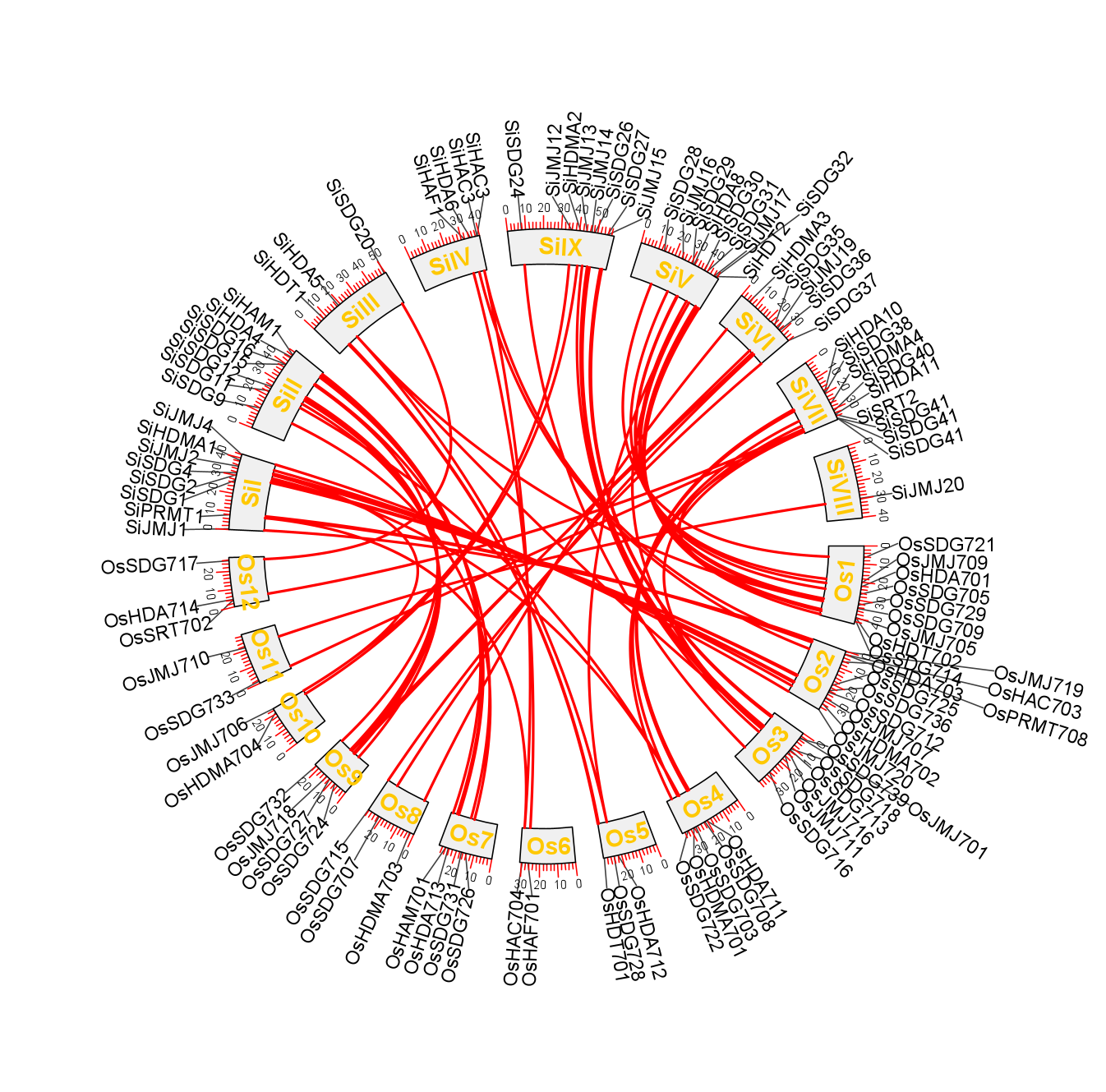


Figure S6-6 Synteny analysis of *HM* genes between *Z. mays* and rice.


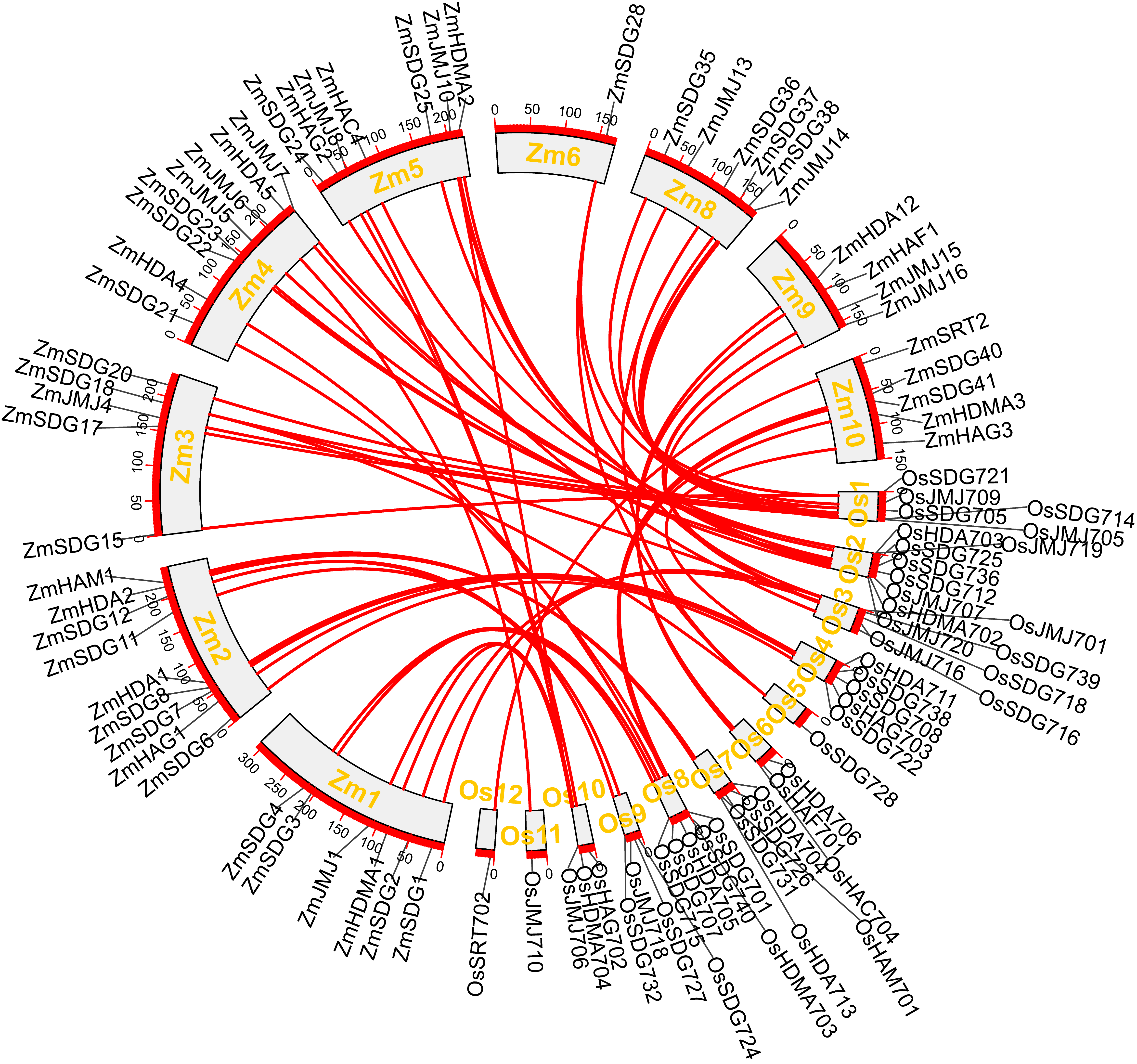

Supplement: Supplementary file 6 — Additional file 6: Figure S6. Synteny analysis of HM genes between each Gramineae species and rice. [file 12870_2021_3332_MOESM6_ESM.docx]
