# Supplementary material for: Genome-wide identification of Gramineae histone modification genes and their potential roles in regulating wheat and maize growth and stress responses
Source: BMC Plant Biol. 2021 Nov 20;21:543. doi: 10.1186/s12870-021-03332-8 (PMC8605605; doi:10.1186/s12870-021-03332-8)

**Figure S7 Promoter analysis of *T. aestivum*, *H. vulgare*, *S. bicolor*, *S. viridis*, *S. italica,* and *Z. mays HM* genes.**

Figure S7-1 Promoter analysis of *TaSDG* and *TaPRMT* genes.


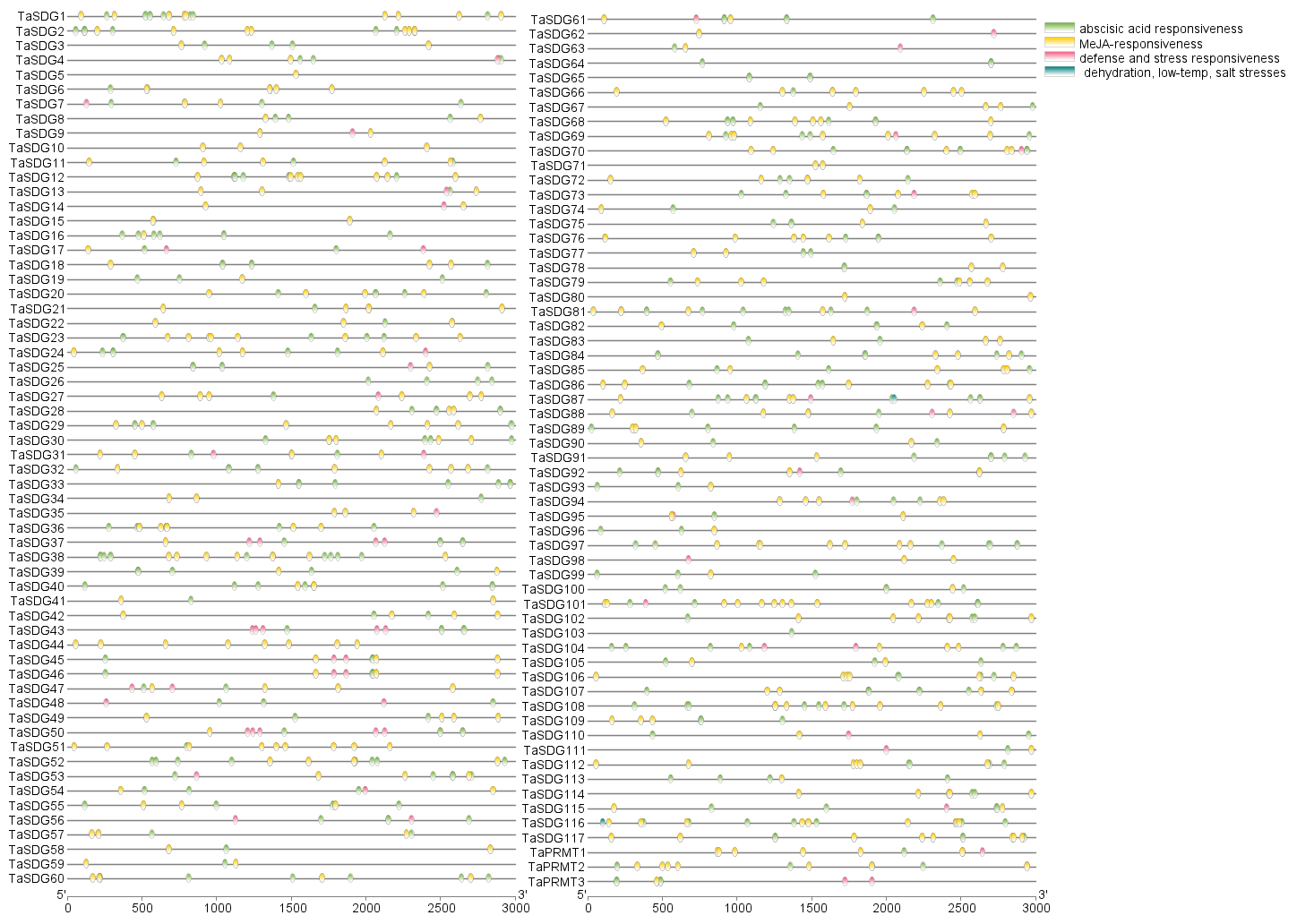


Figure S7-2 Promoter analysis of *TaHDMA* and *TaJMJ* genes.


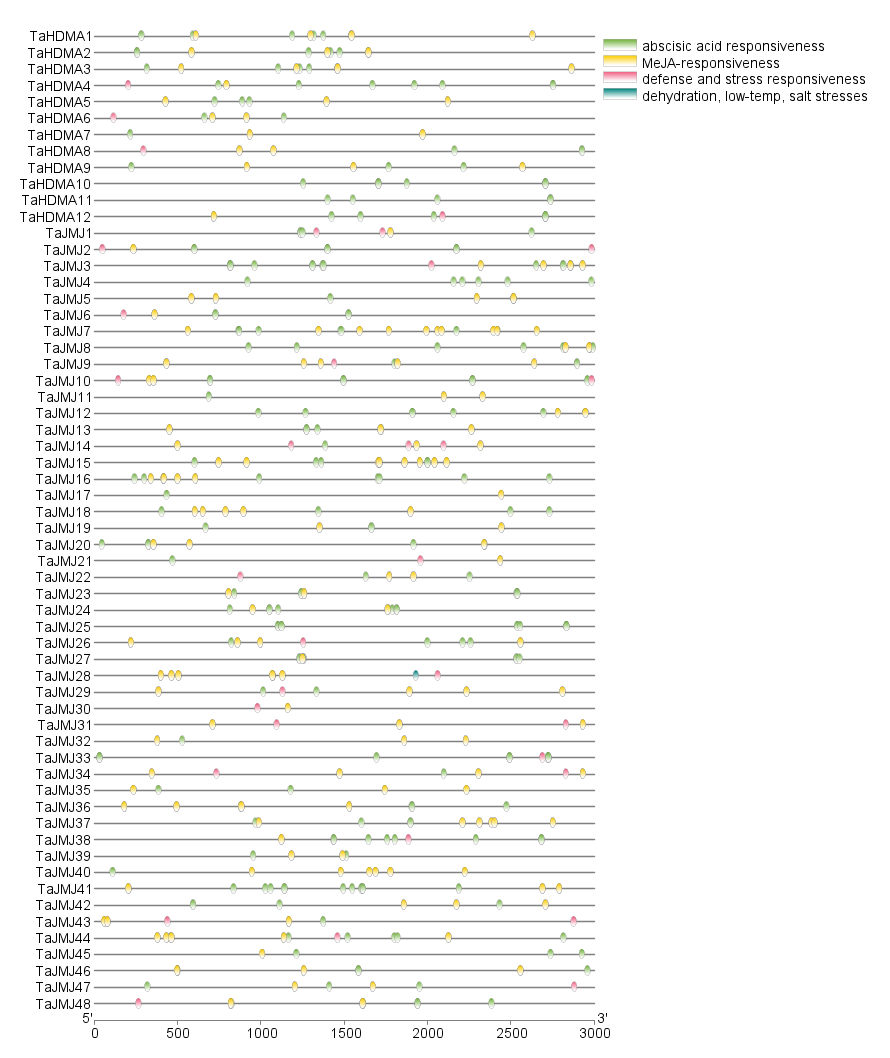


Figure S7-3 Promoter analysis of *TaHAG*, *TaHAM*, *TaHAC*, and *TaHAF* genes.


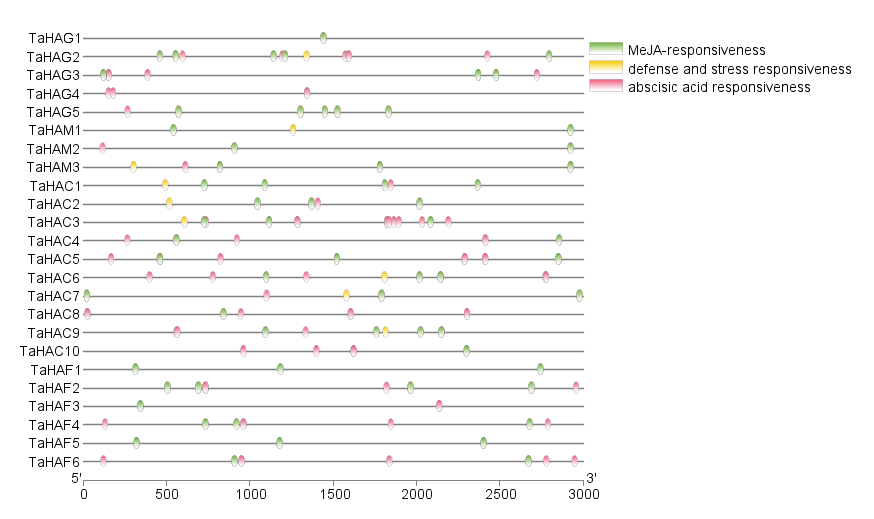


Figure S7-4 Promoter analysis of *TaHDA*, *TaSRT*, and *TaHDT* genes


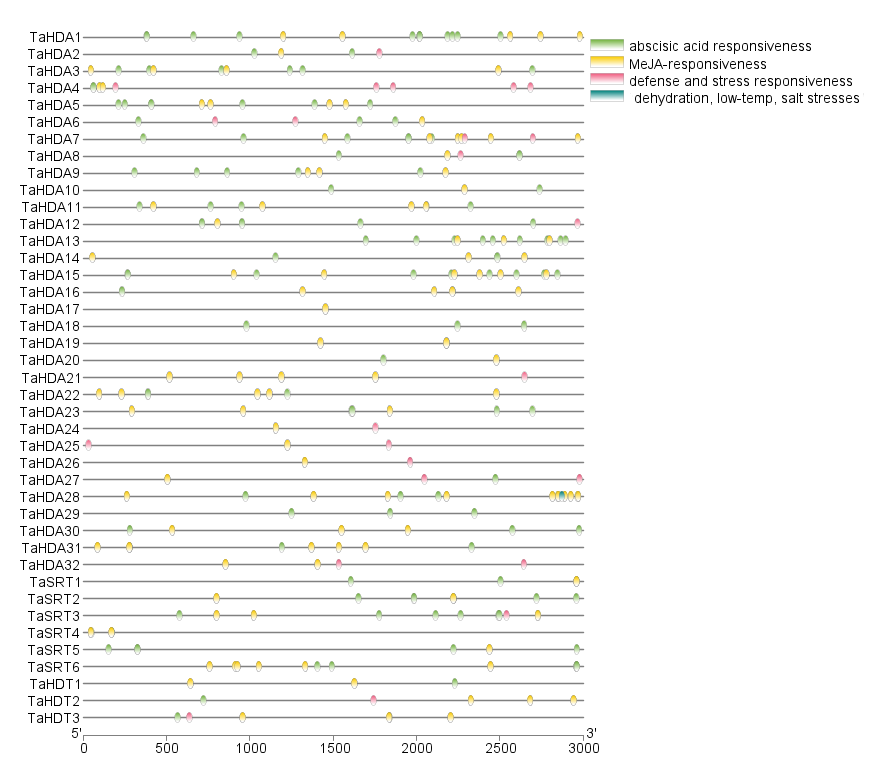


Figure S7-5 Promoter analysis of *HvHM* genes.


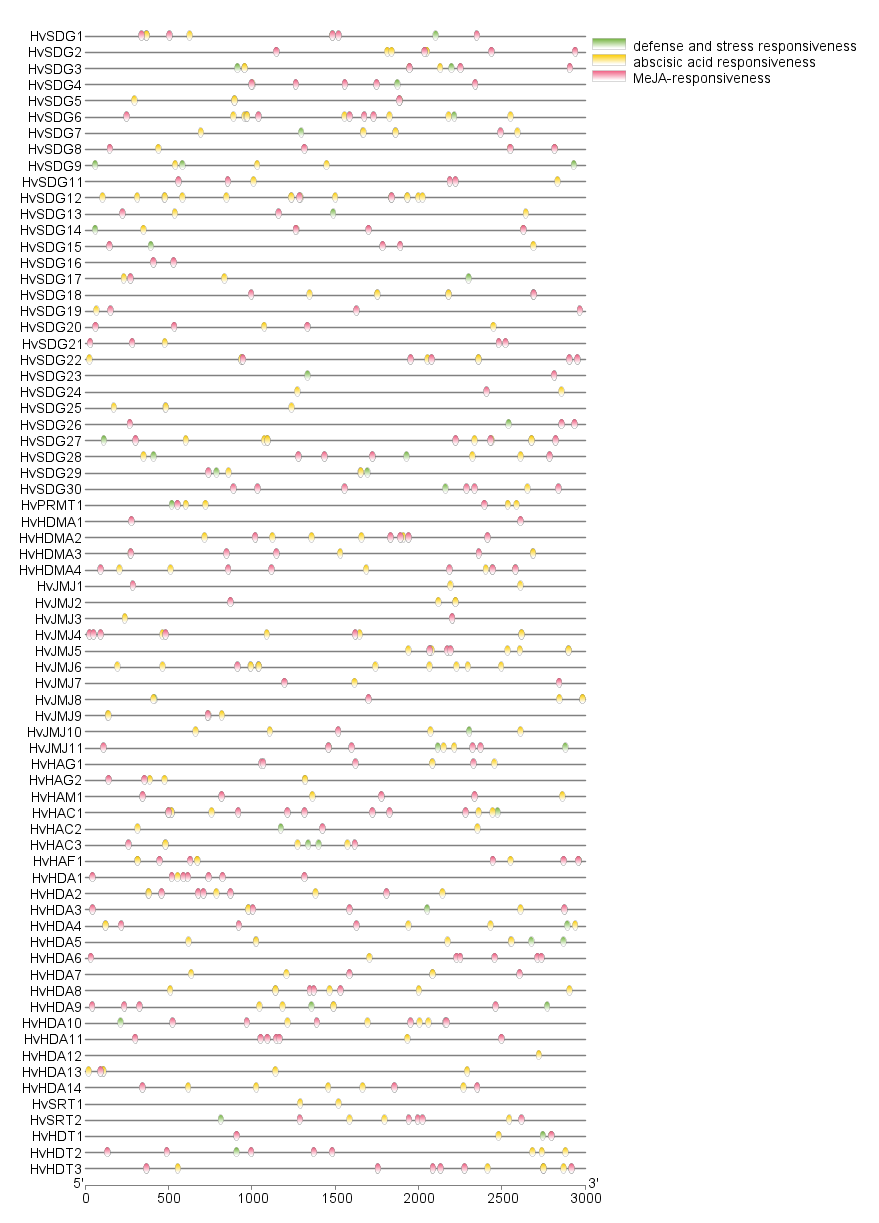


Figure S7-6 Promoter analysis of *SbHM* genes.


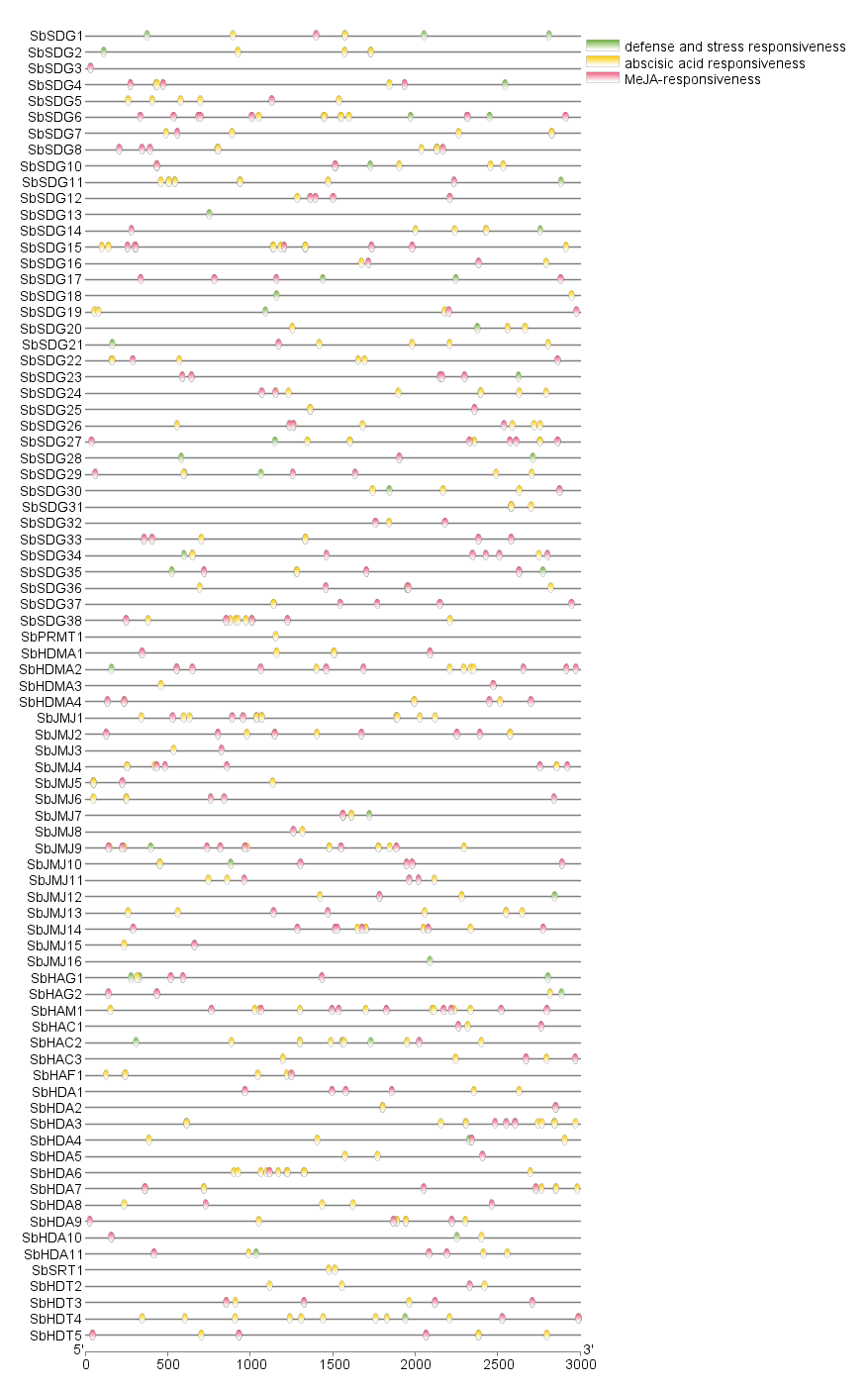


Figure S7-7 Promoter analysis of *SvHM* genes.


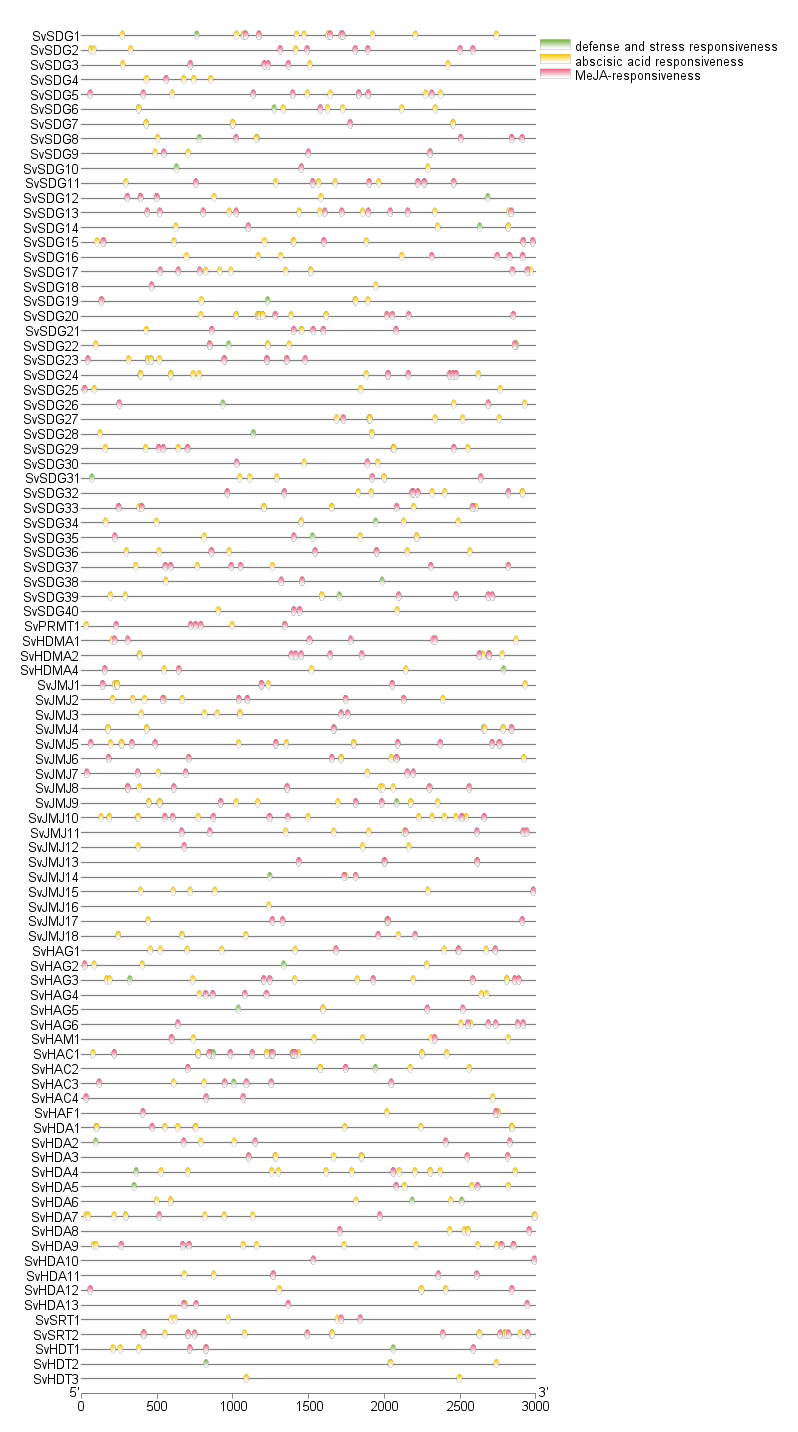


Figure S7-8 Promoter analysis of *SiHM* genes.


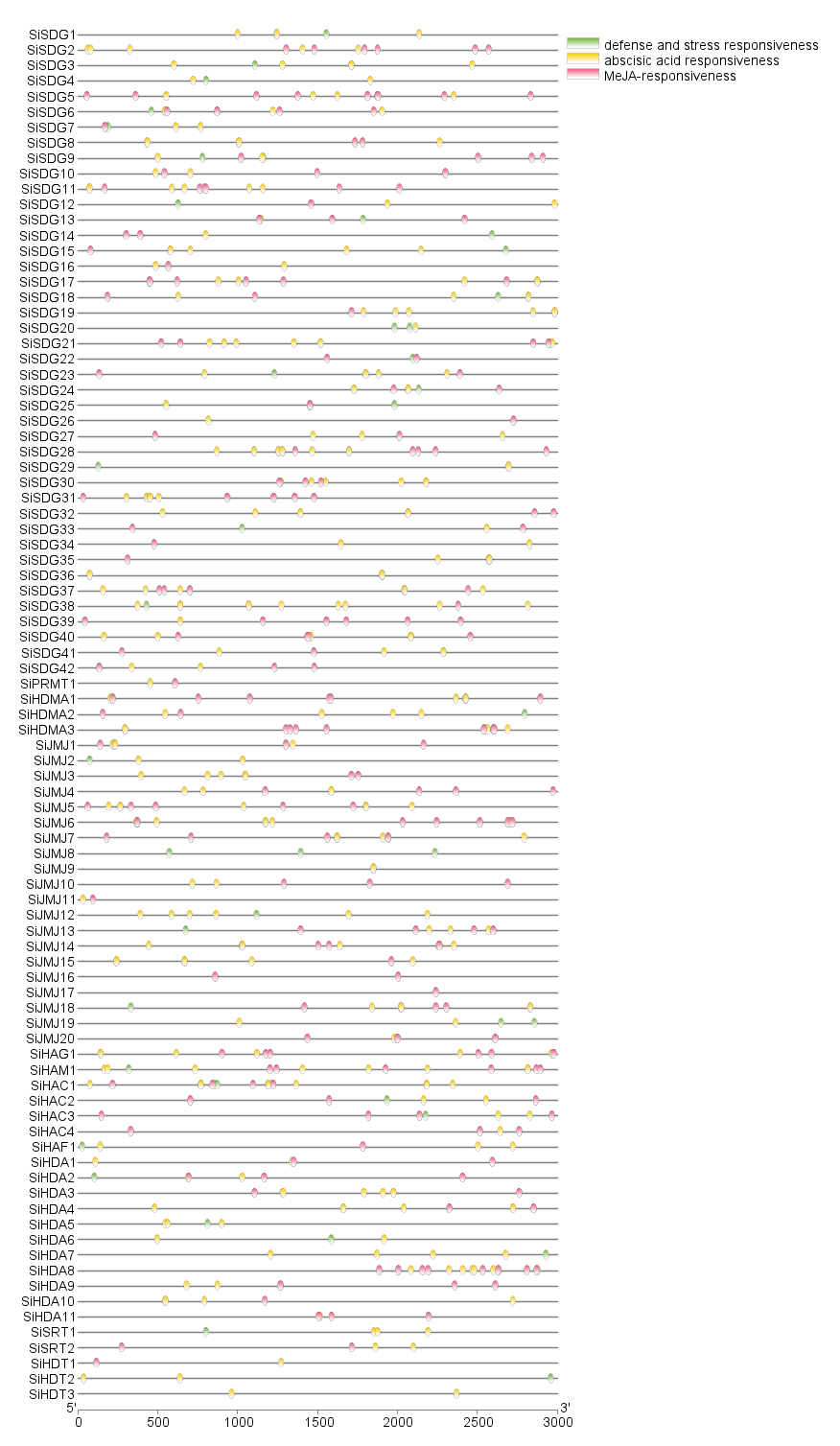


Figure S7-9 Promoter analysis of *ZmHM* genes.


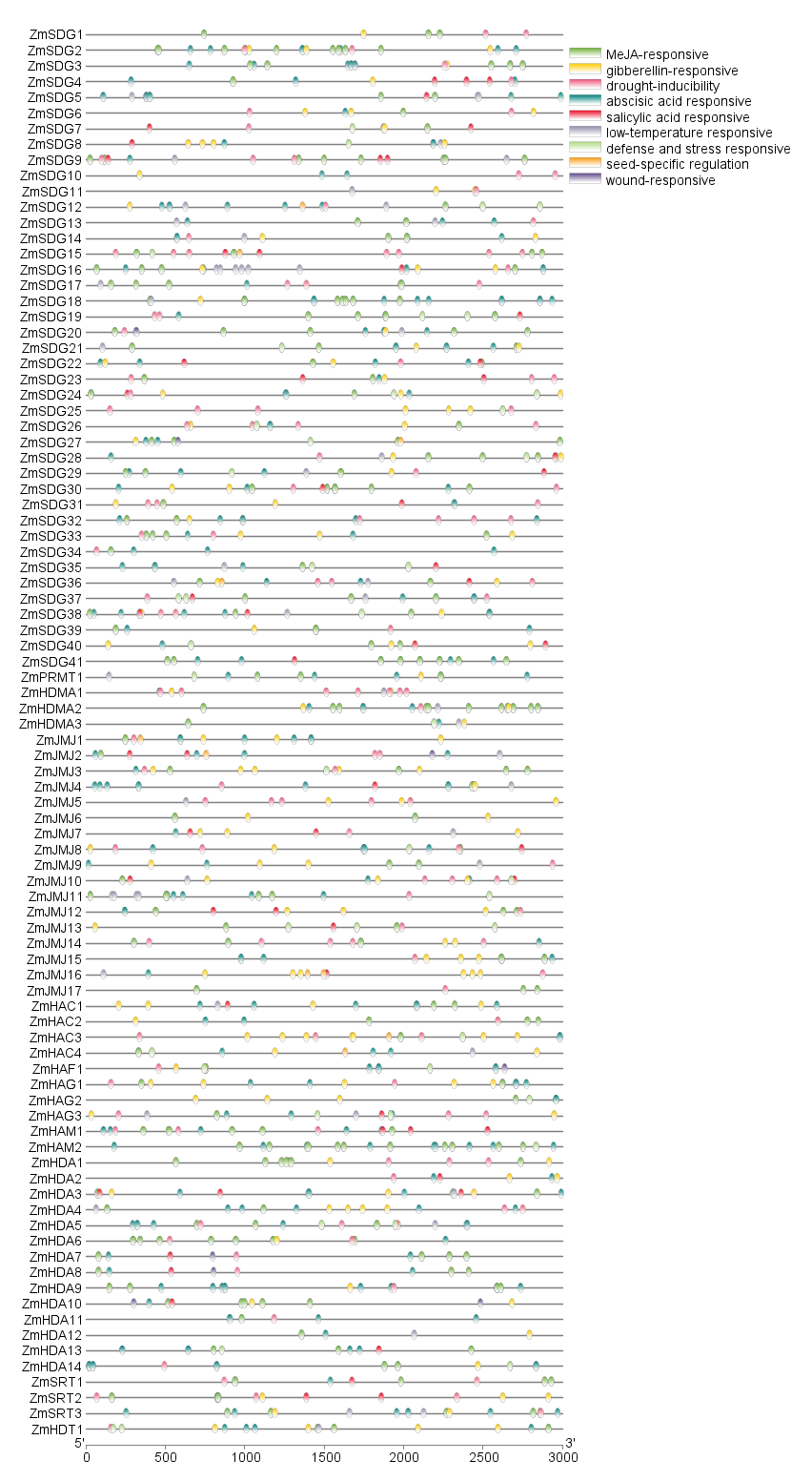

Supplement: Supplementary file 7 — Additional file 7: Figure S7. Promoter analysis of T. aestivum, H. vulgare, S. bicolor, S. viridis, S. italica, and Z. mays HM genes. [file 12870_2021_3332_MOESM7_ESM.docx]
