# Supplementary material for: Genome-wide identification of Gramineae histone modification genes and their potential roles in regulating wheat and maize growth and stress responses
Source: BMC Plant Biol. 2021 Nov 20;21:543. doi: 10.1186/s12870-021-03332-8 (PMC8605605; doi:10.1186/s12870-021-03332-8)

**Figure S8 Gene structure analysis of *HM* genes.**

Figure S8-1 Gene structure analysis of *TaSDG* and *TaPRMT* genes.


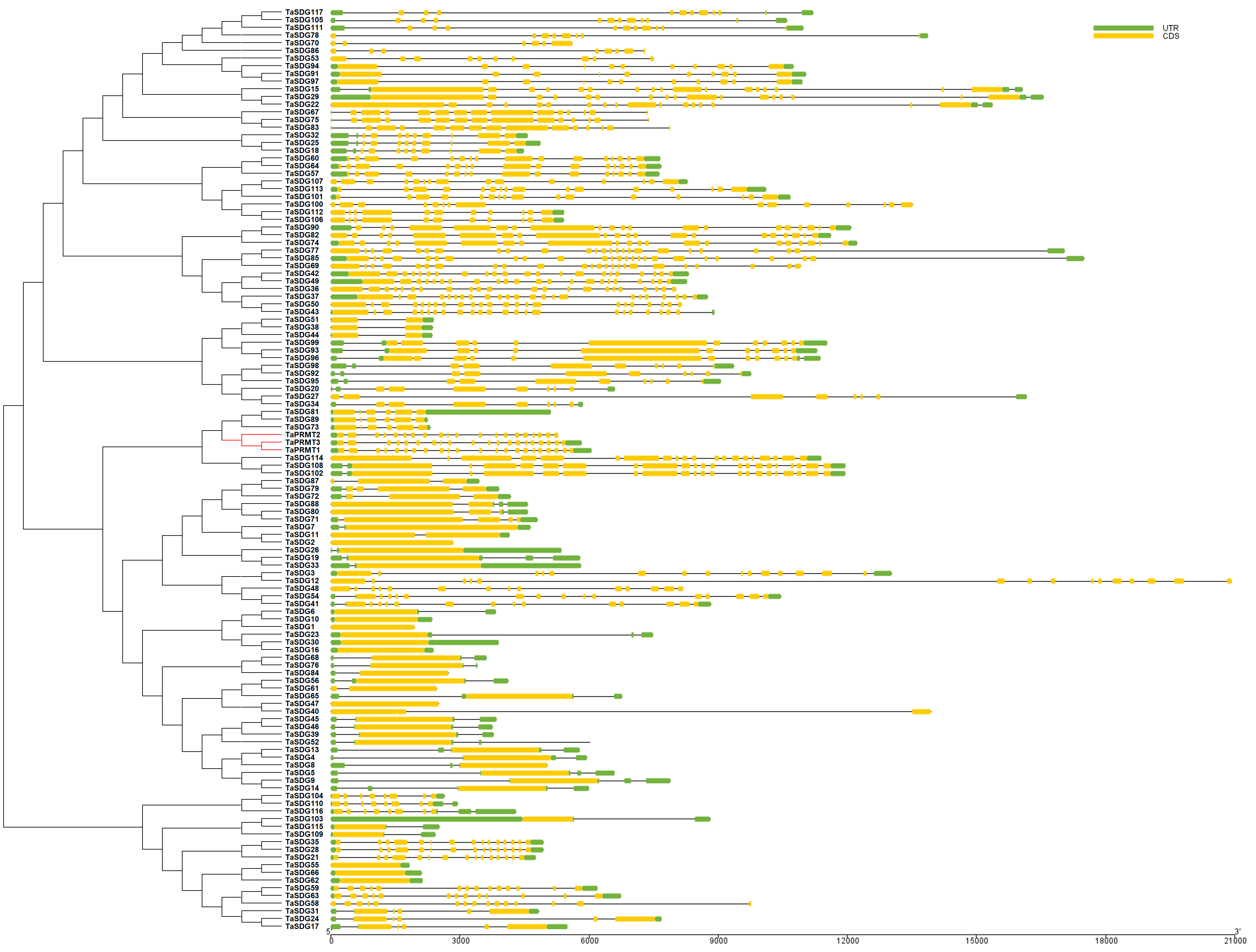


Figure S8-2 Gene structure analysis of *TaHDMA* and *TaJMJ* genes.


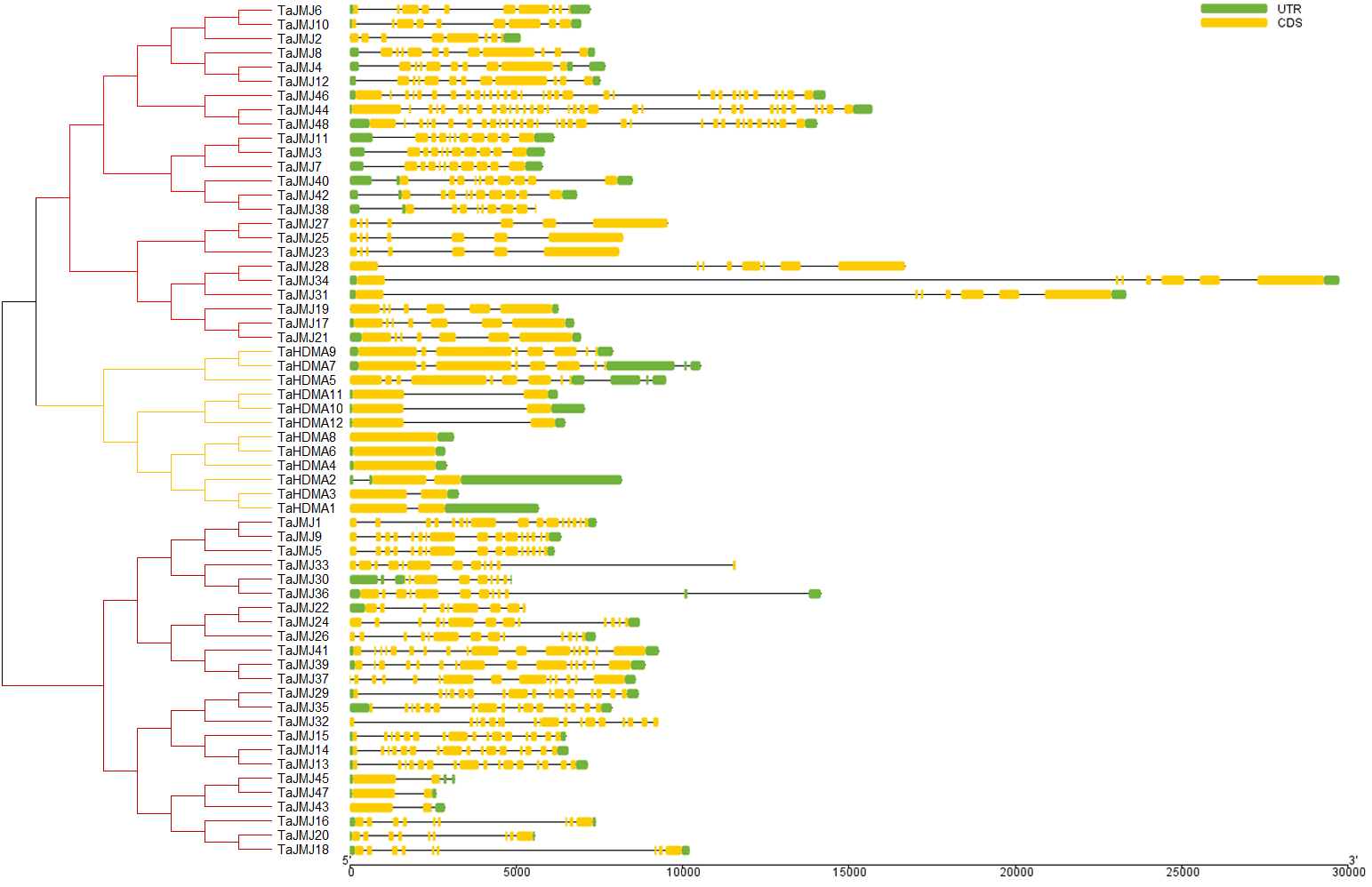


Figure S8-3 Gene structure analysis of *TaHAG,* *TaHAM*, *TaHAC*, and *TaHAF* genes.


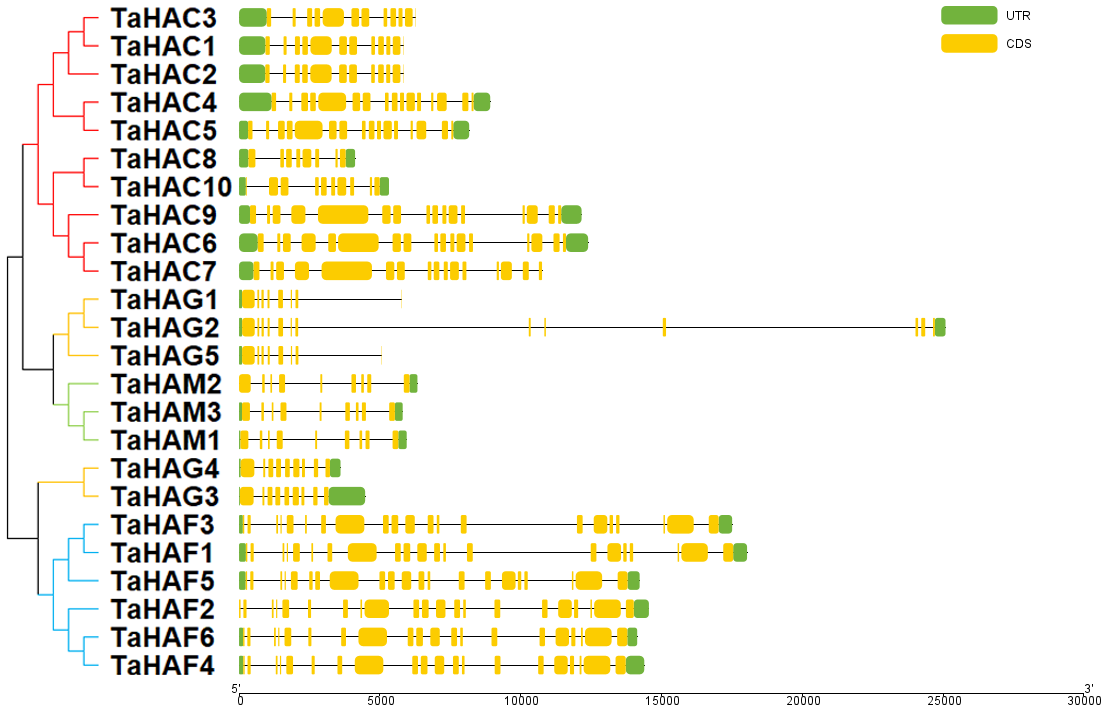


Figure S8-4 Gene structure analysis of *TaHDA,* *TaSRT*, and *TaHDT* genes.


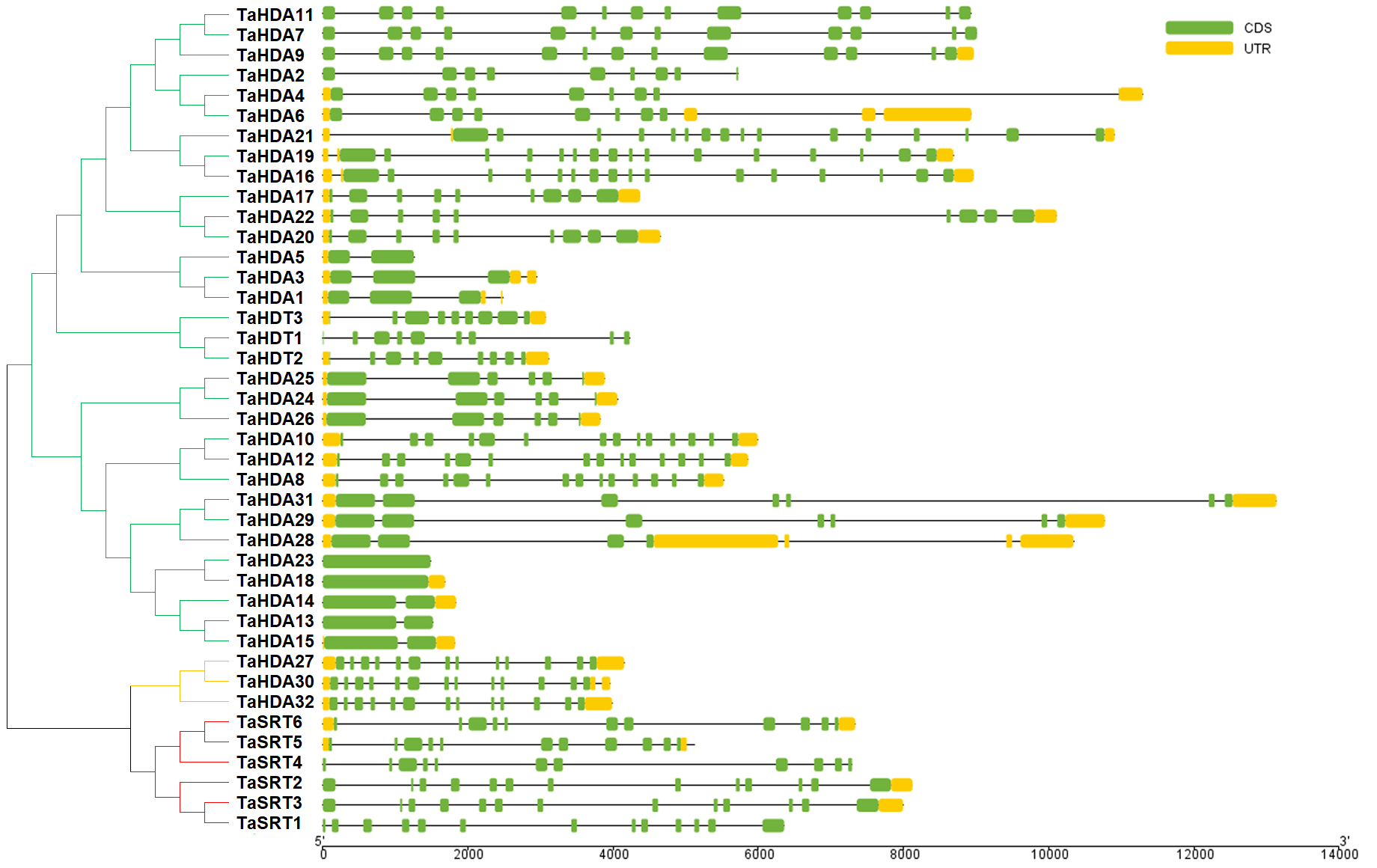


Figure S8-5 Gene structure analysis of *HvSDG* and *HvPRMT* genes.


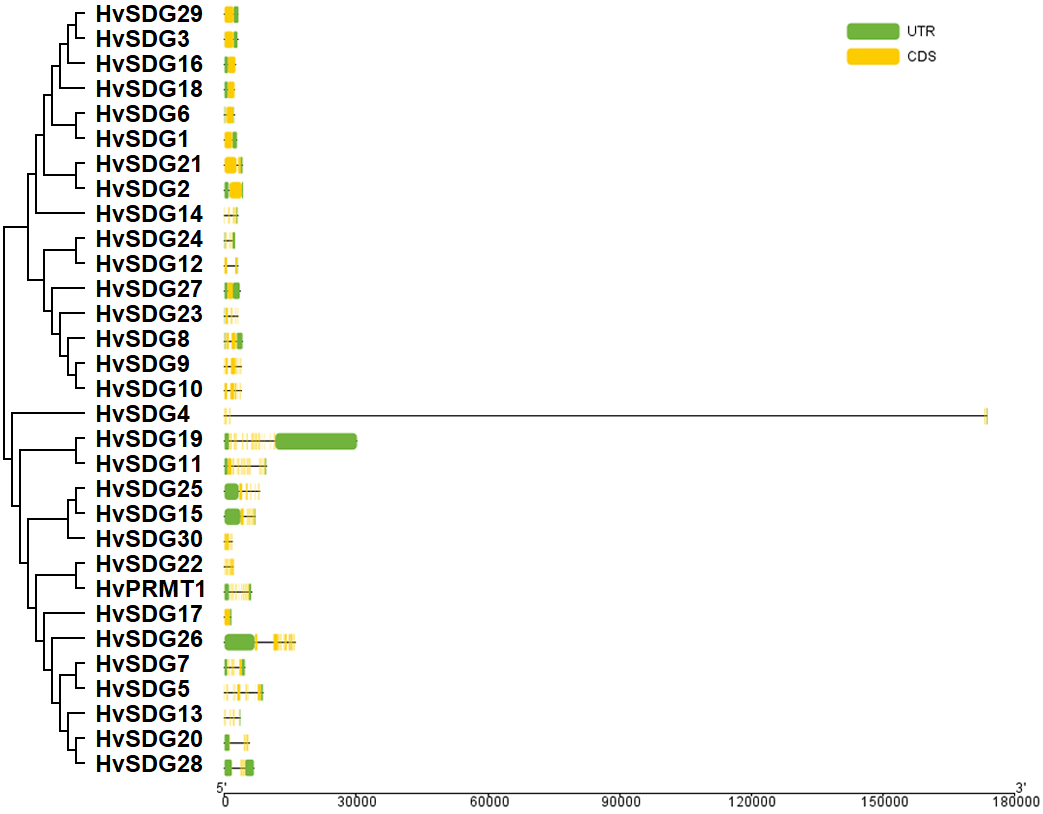


Figure S8-6 Gene structure analysis of *HvHDMA* and *HvJMJ* genes.


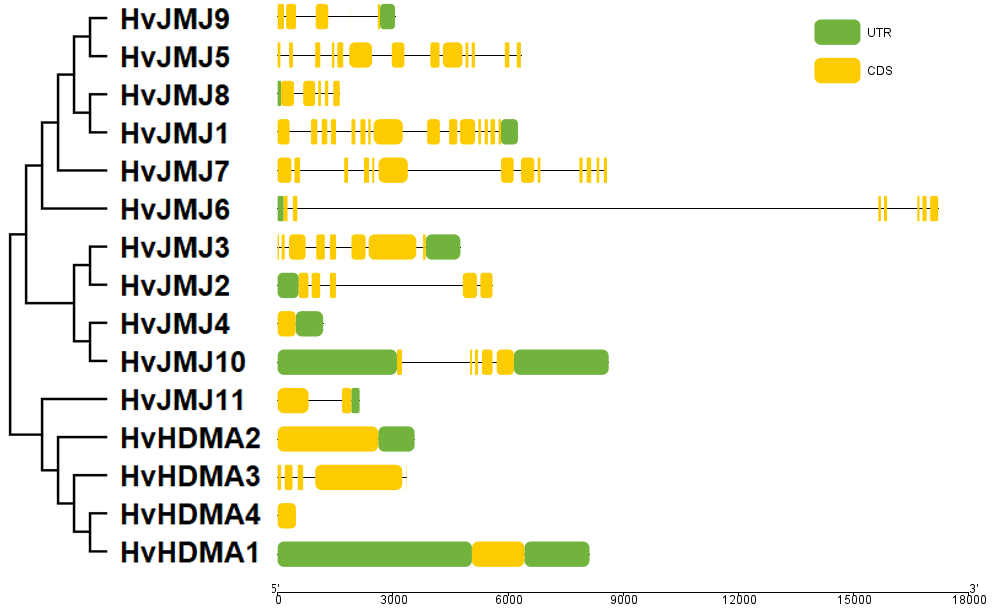


Figure S8-7 Gene structure analysis of *HvHAG,* *HvHAM*, *HvHAC*, and *HvHAF* genes.


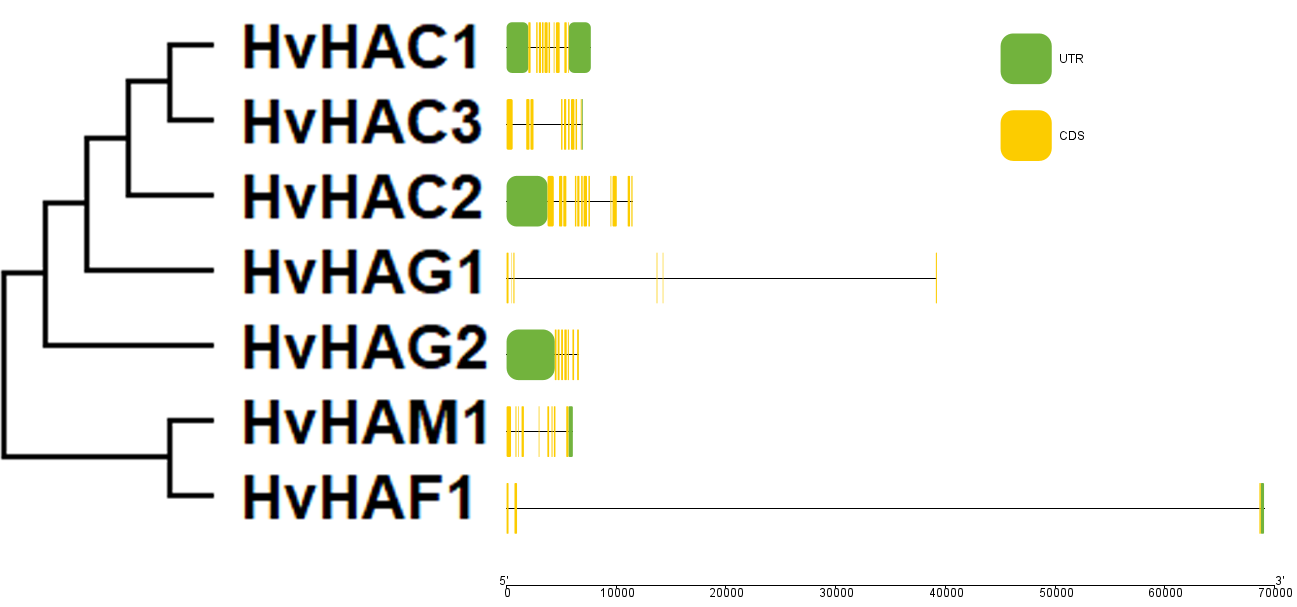


Figure S8-8 Gene structure analysis of *HvHDA,* *HvSRT*, and *HvHDT* genes.


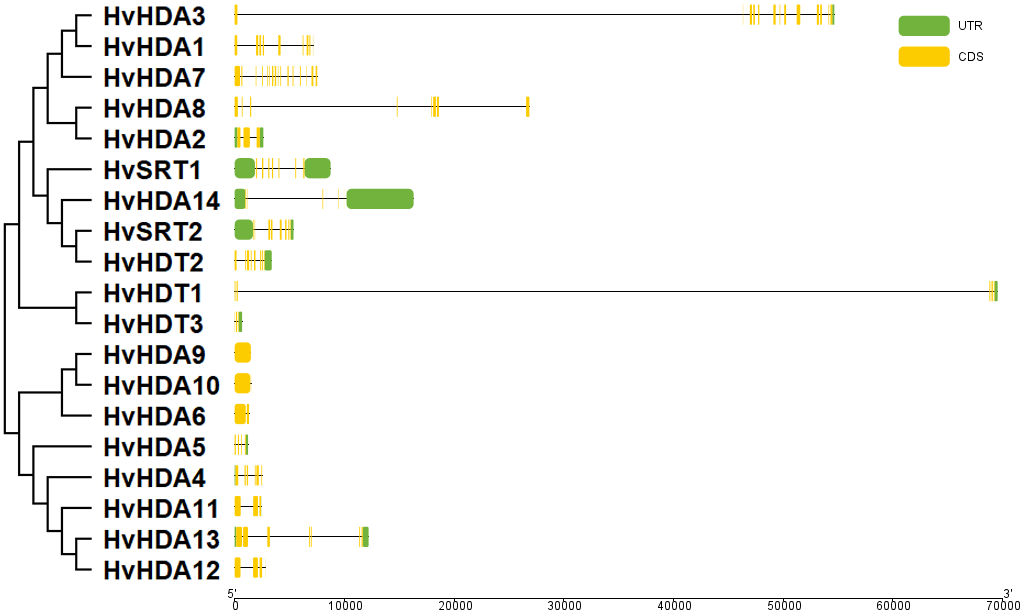


Figure S8-9 Gene structure analysis of *SbSDG* and *SbPRMT* genes.


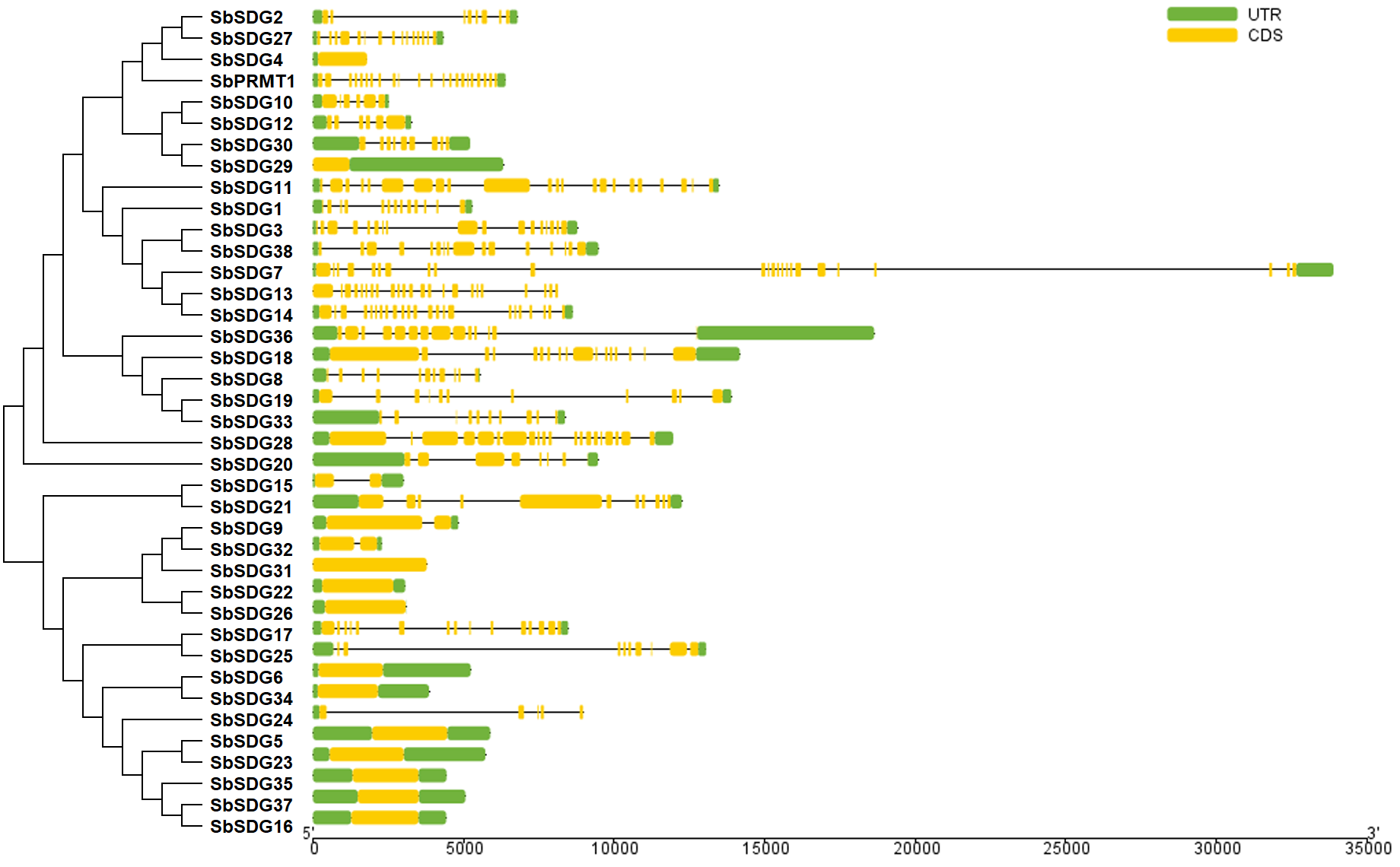


Figure S8-10 Gene structure analysis of *SbHDMA* and *SbJMJ* genes.


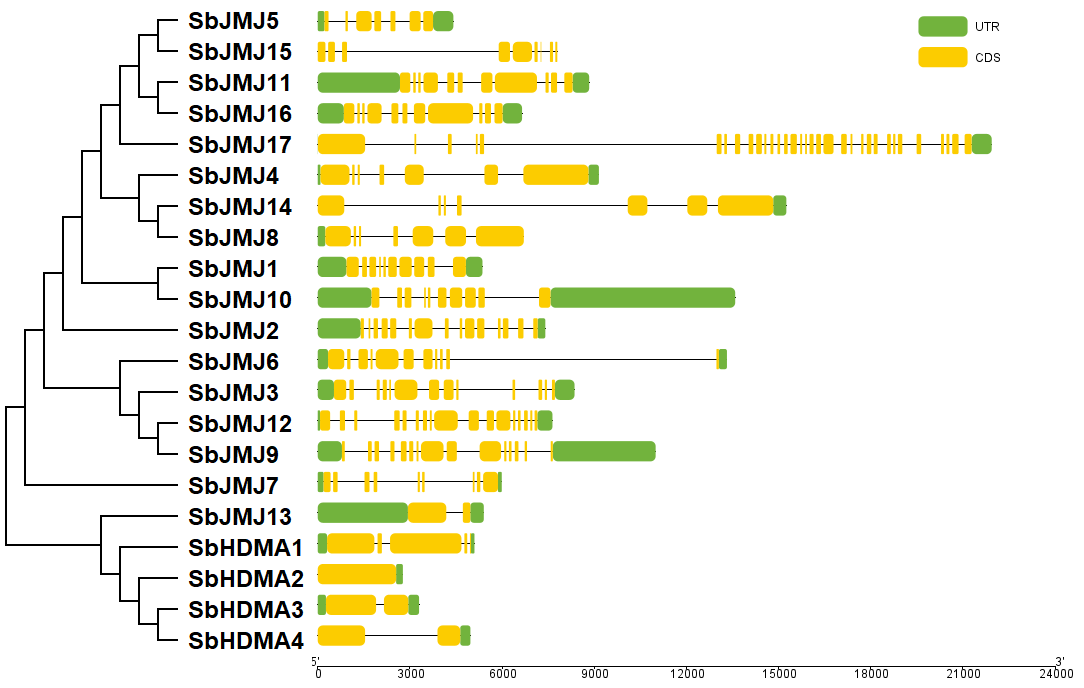


Figure S8-11 Gene structure analysis of *SbHAG,* *SbHAM*, *SbHAC*, and *SbHAF* genes.


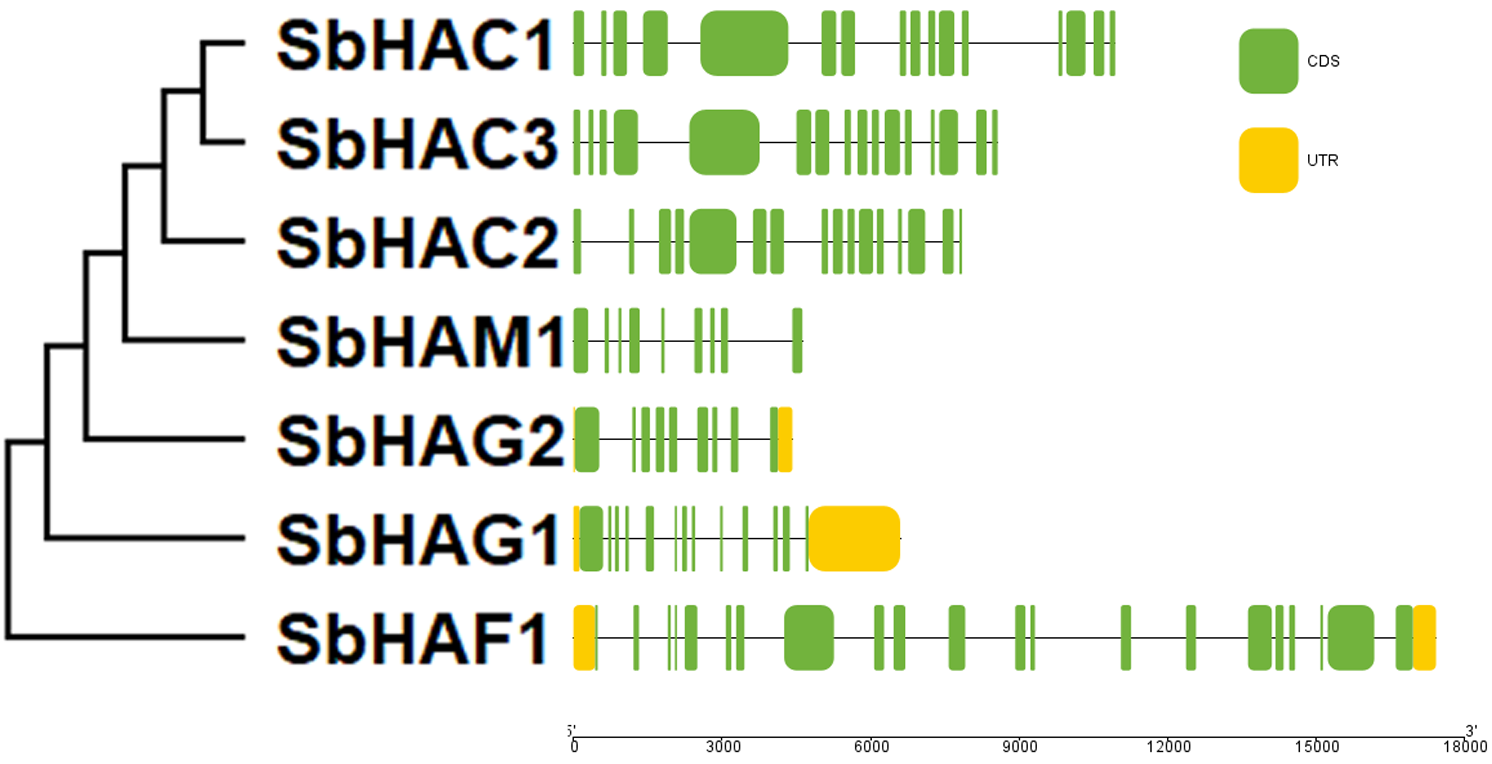


Figure S8-12 Gene structure analysis of *SbHDA,* *SbSRT*, and *SbHDT* genes.


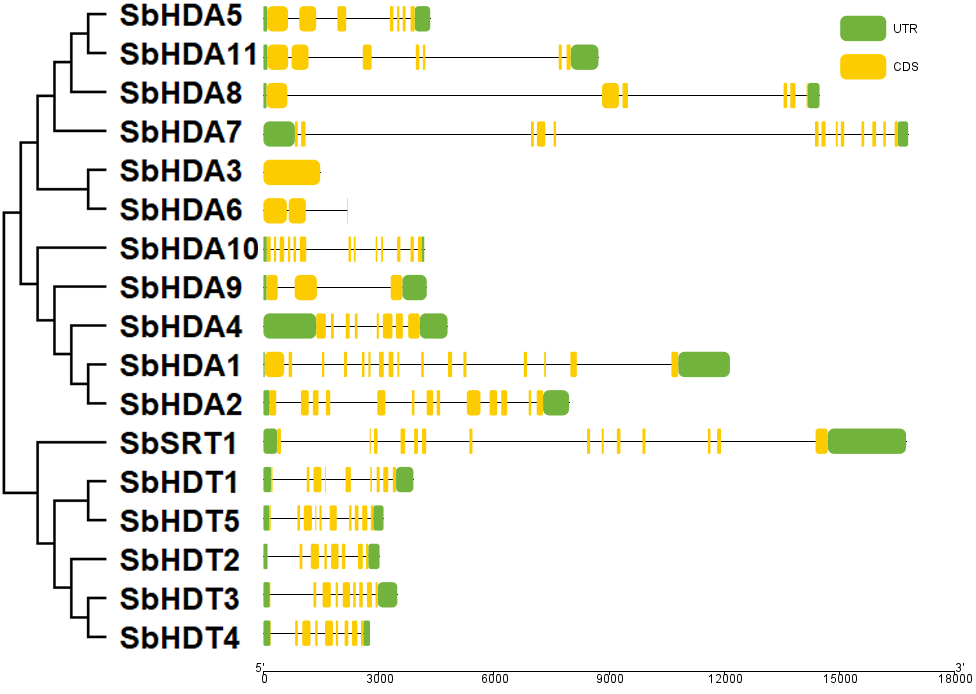


Figure S8-13 Gene structure analysis of *SvSDG* and *SvPRMT* genes.


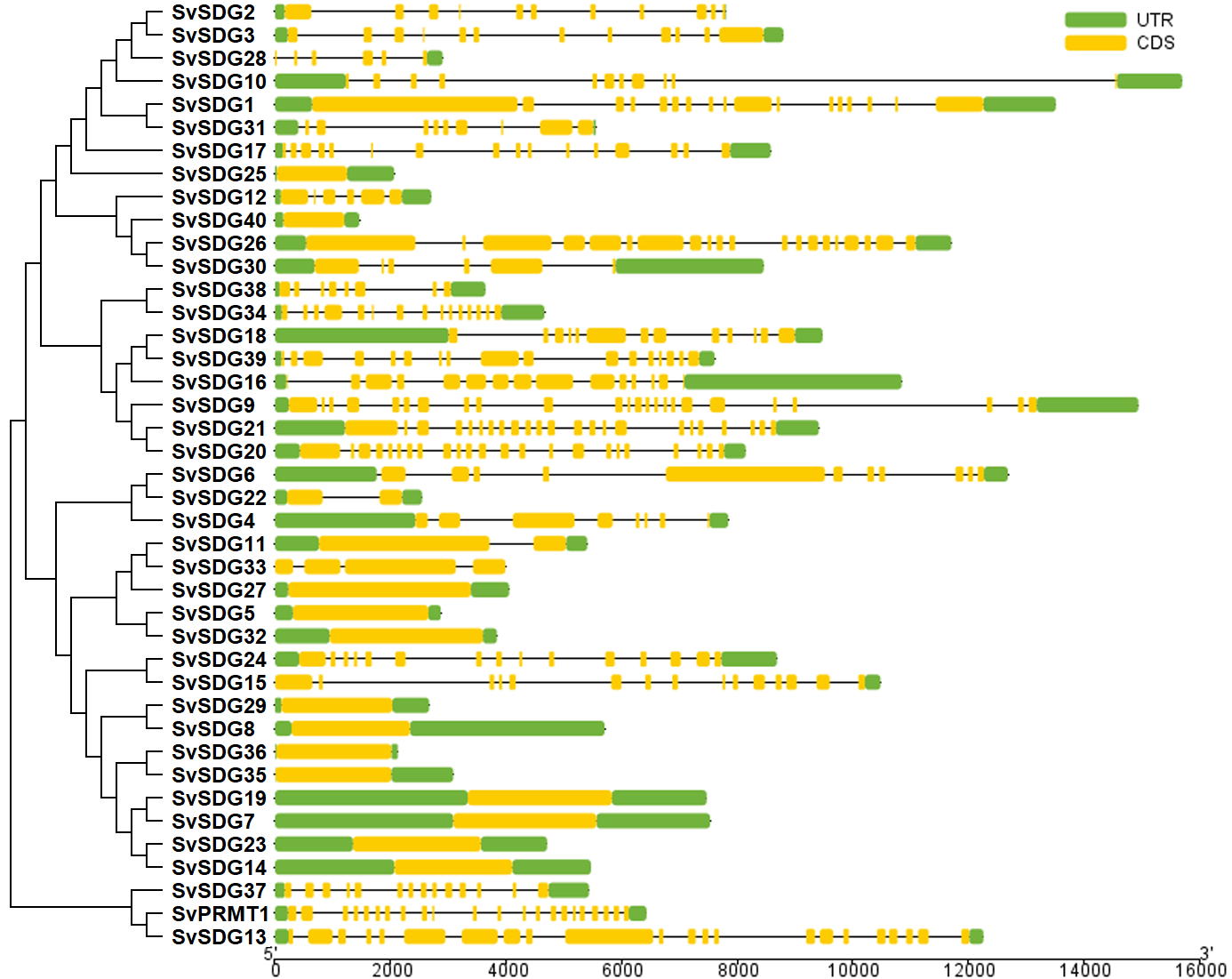


Figure S8-14 Gene structure analysis of *SvHDMA* and *SvJMJ* genes.


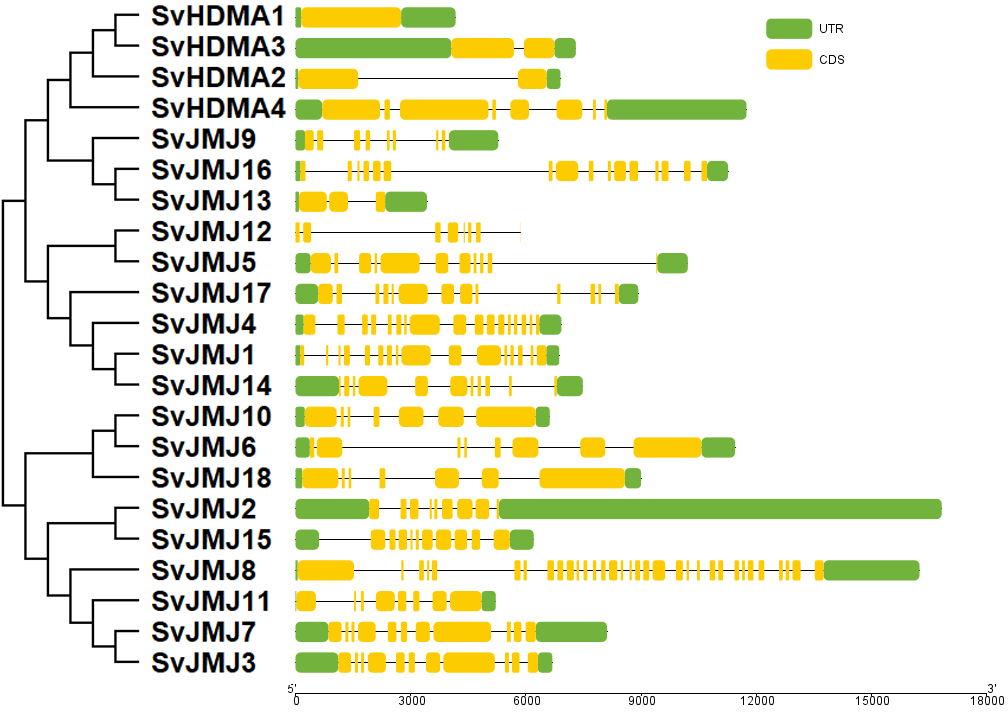


Figure S8-15 Gene structure analysis of *SvHAG,* *SvHAM*, *SvHAC*, and *SvHAF* genes.


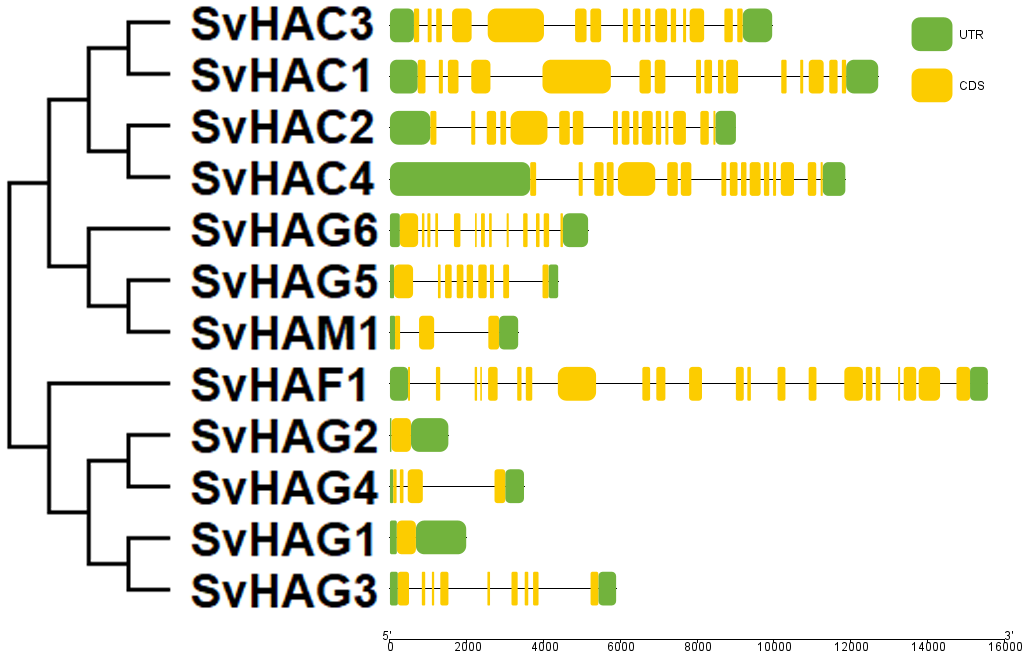


Figure S8-16 Gene structure analysis of *SvHDA,* *SvSRT*, and *SvHDT* genes.


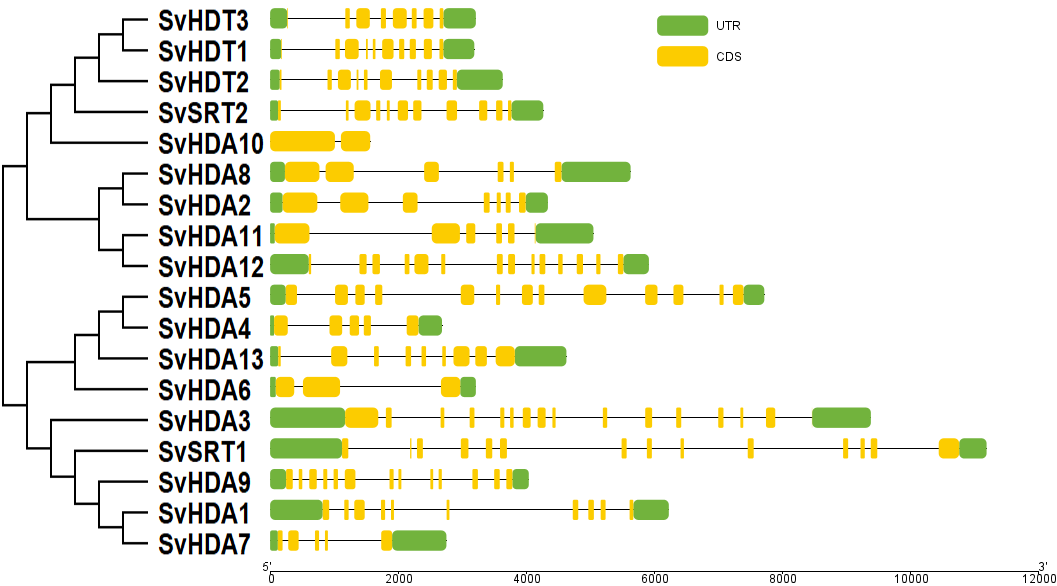


Figure S8-17 Gene structure analysis of *SiSDG* and *SiPRMT* genes.


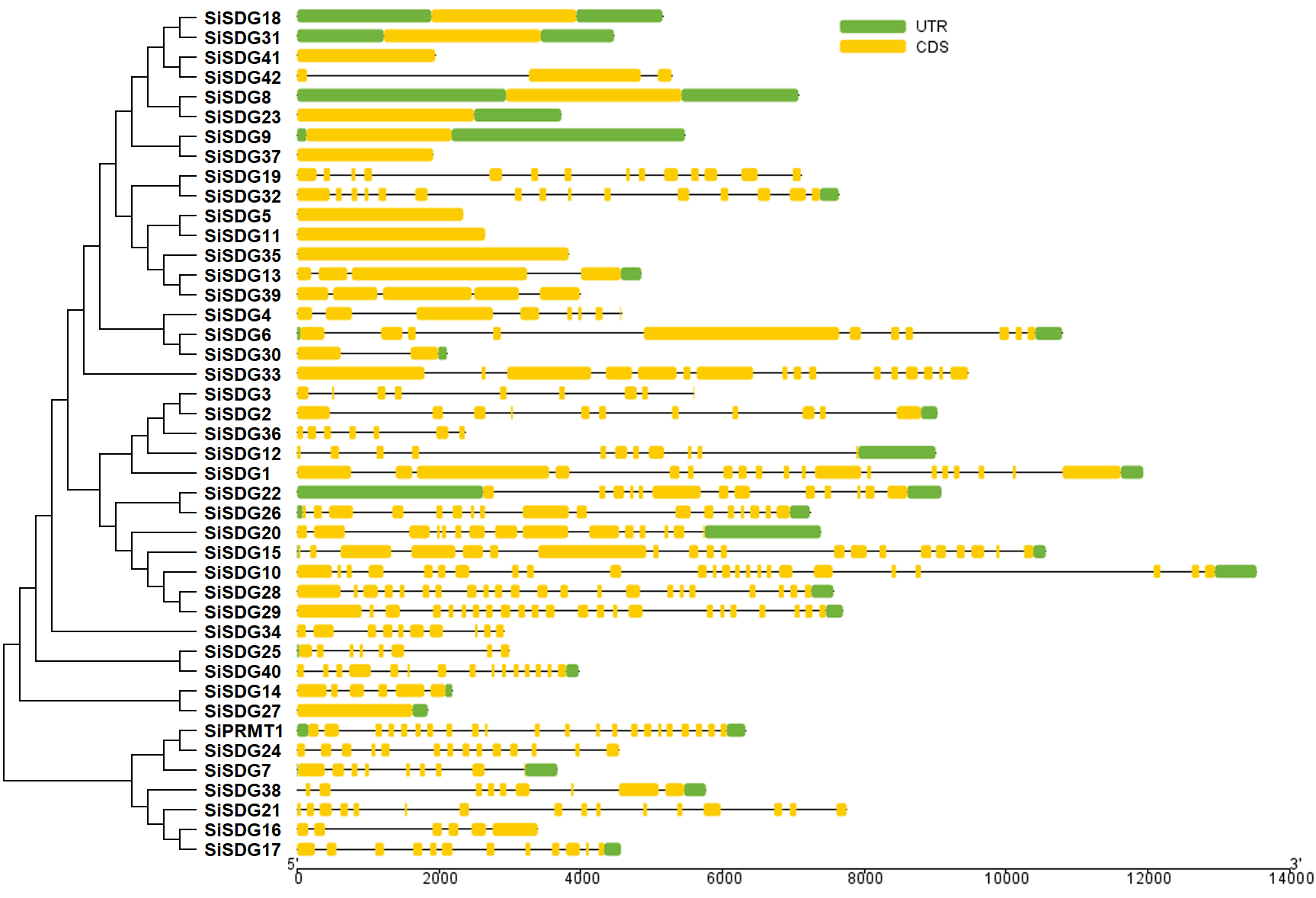


Figure S8-18 Gene structure analysis of *SiHDMA* and *SiJMJ* genes.


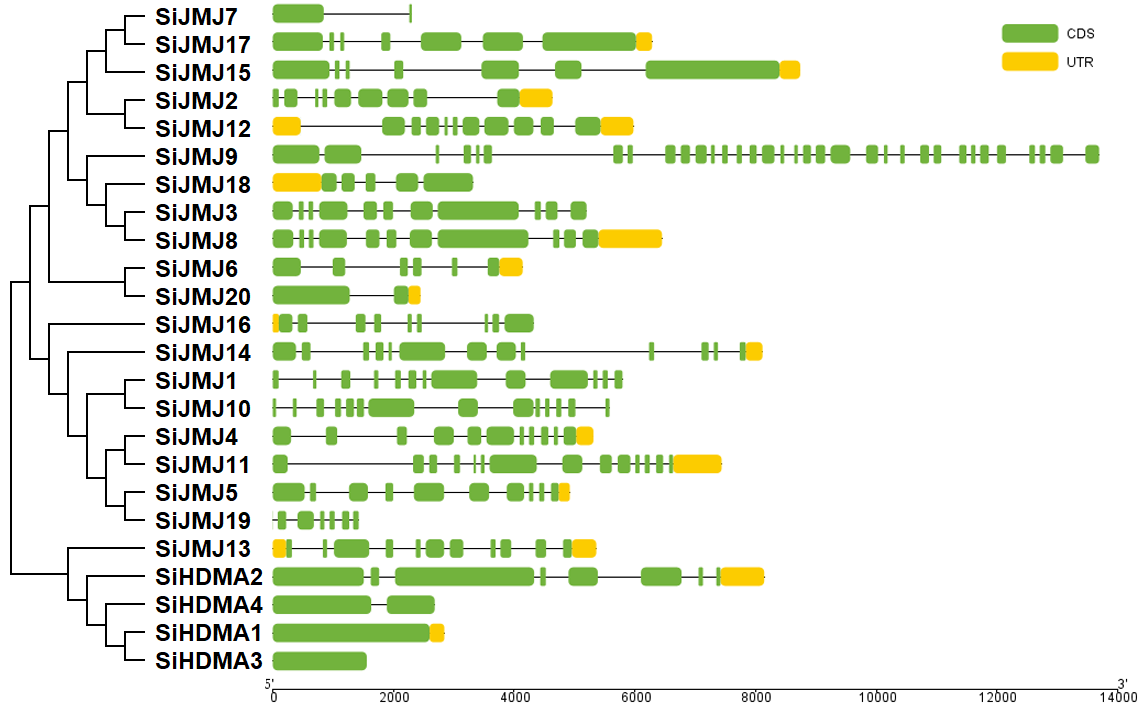


Figure S8-19 Gene structure analysis of *SiHAG,* *SiHAM*, *SiHAC*, and *SiHAF* genes.


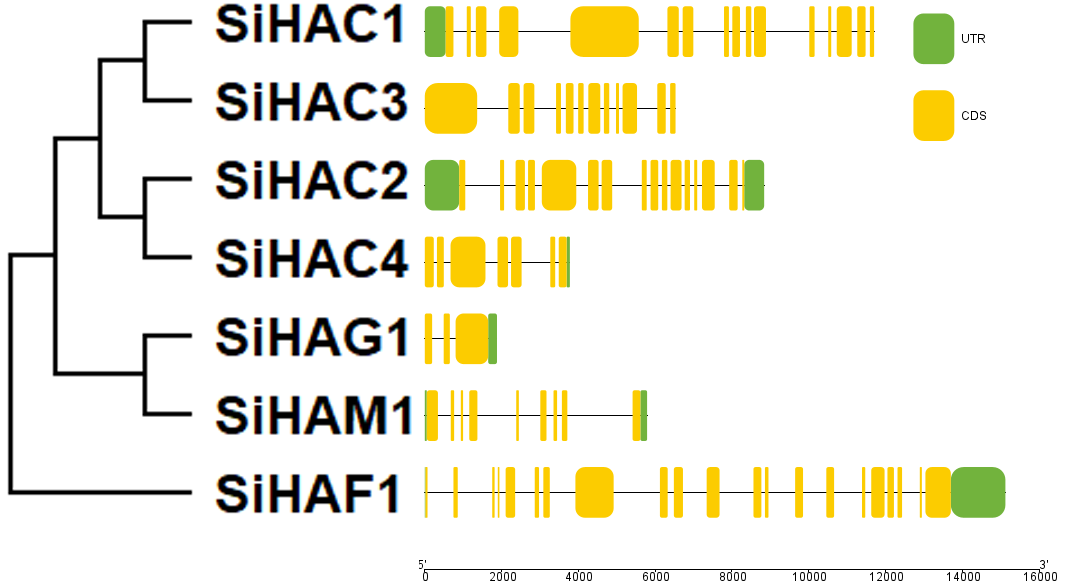


Figure S8-20 Gene structure analysis of *SiHDA,* *SiSRT*, and *SiHDT* genes.


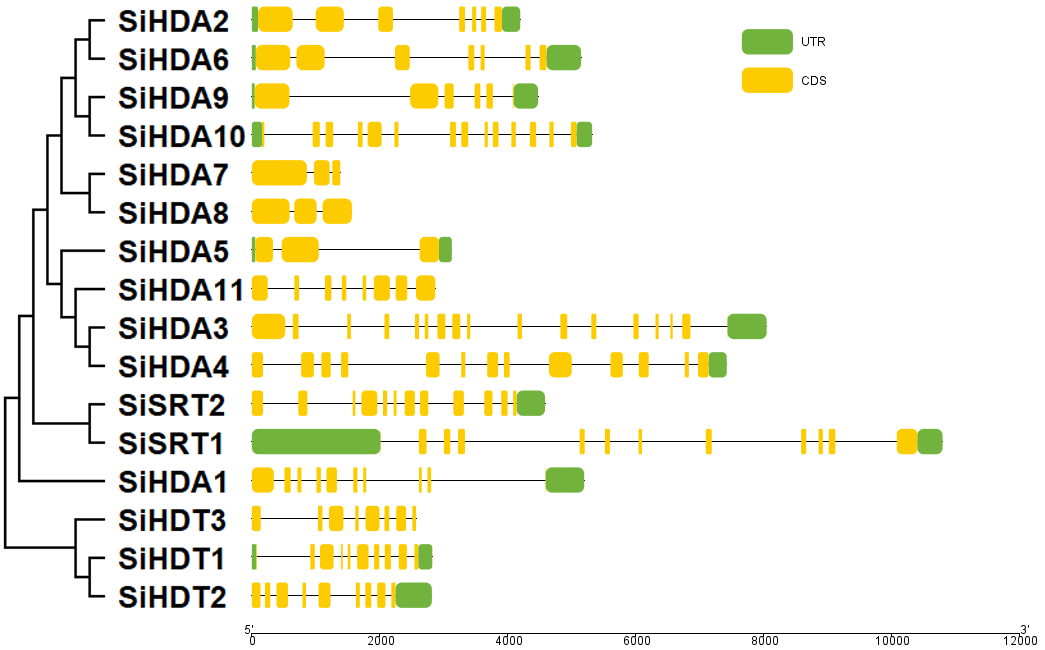


Figure S8-21 Gene structure analysis of *ZmSDG* and *ZmPRMT* genes.


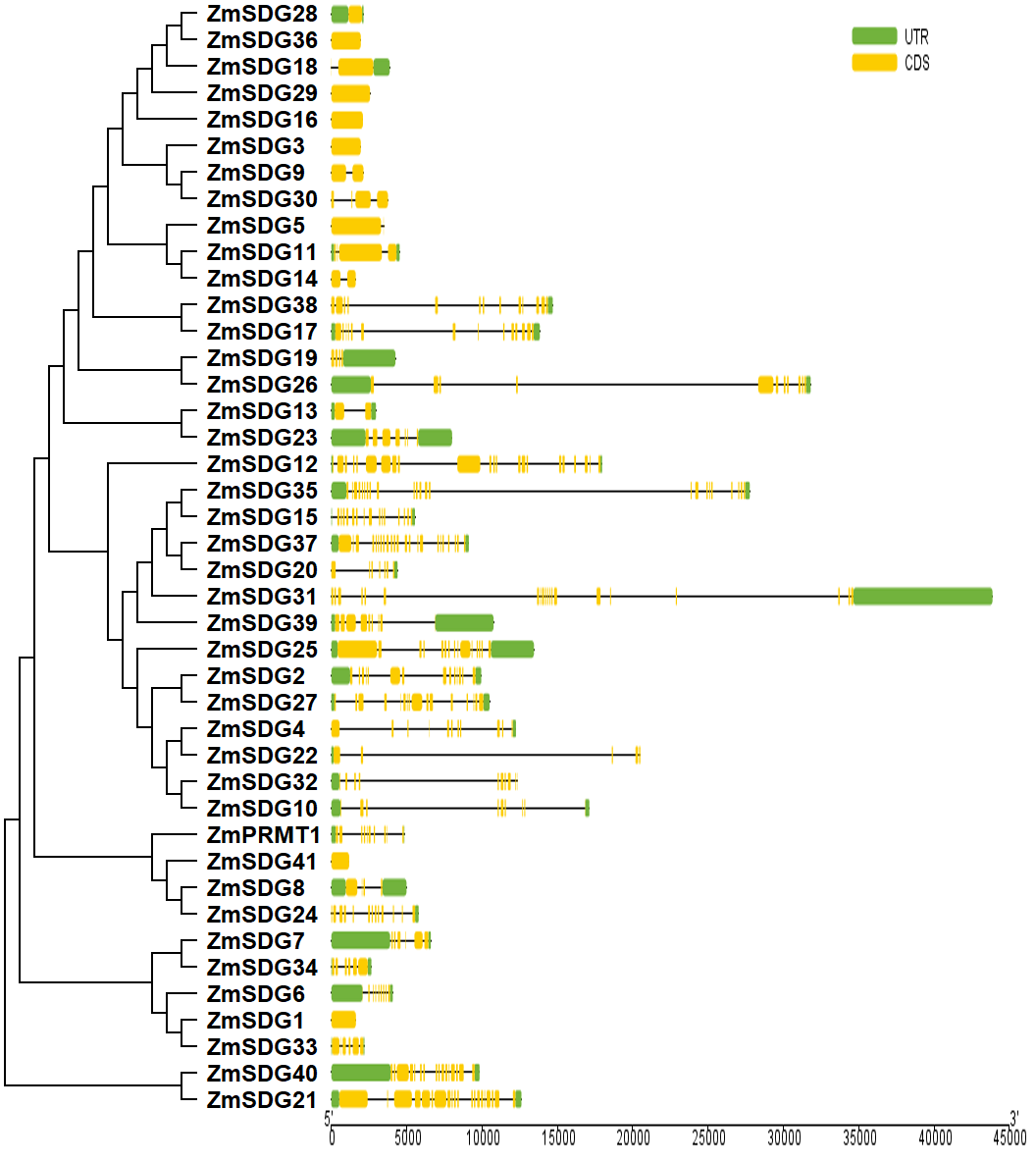


Figure S8-22 Gene structure analysis of *ZmHDMA* and *ZmJMJ* genes.


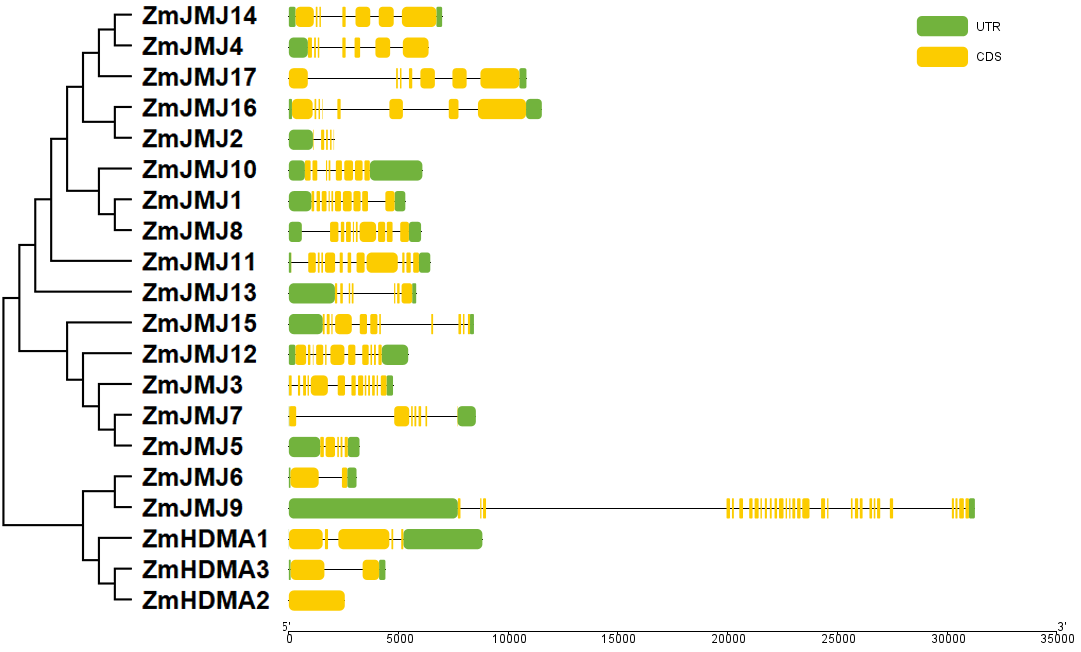


Figure S8-23 Gene structure analysis of *ZmHAG,* *ZmHAM*, *ZmHAC*, and *ZmHAF* genes.


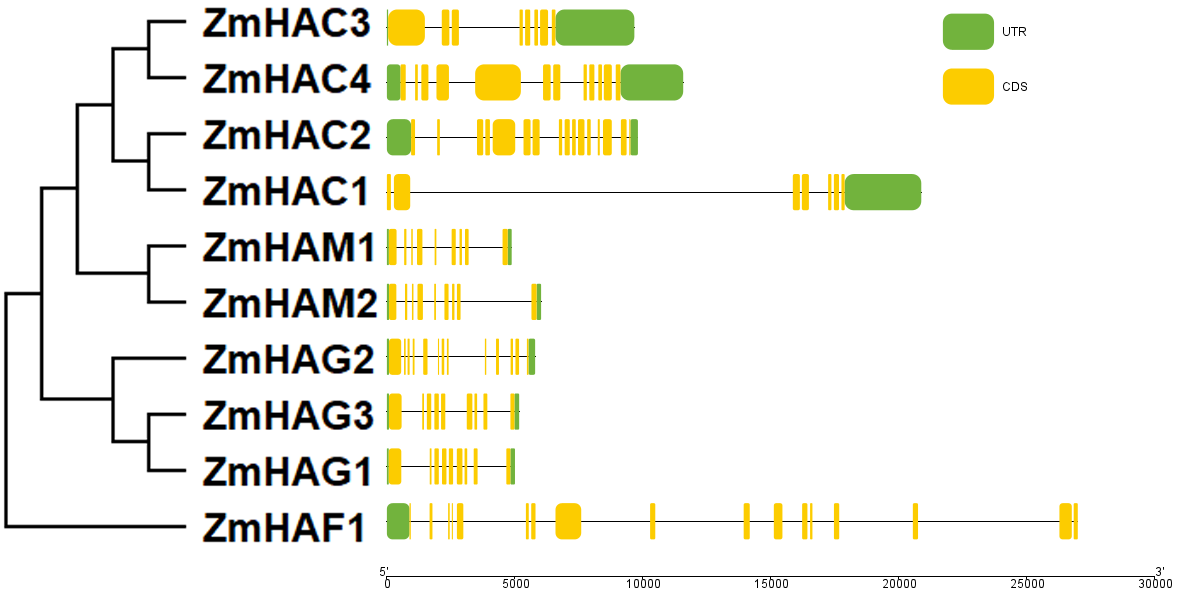


Figure S8-24 Gene structure analysis of *ZmHDA,* *ZmSRT*, and *ZmHDT* genes.


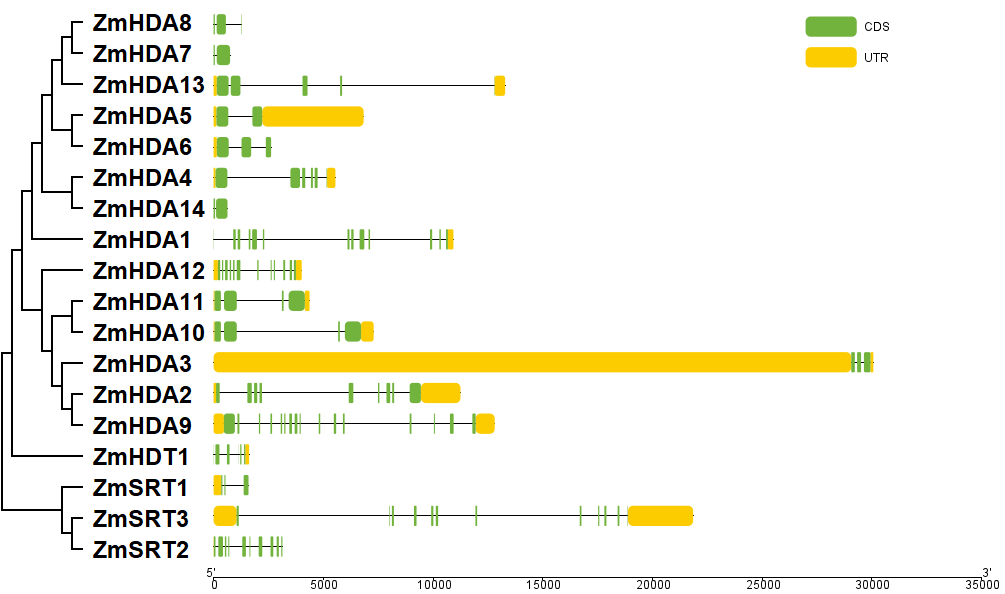

Supplement: Supplementary file 8 — Additional file 8: Figure S8. Gene structure analysis of HM genes. [file 12870_2021_3332_MOESM8_ESM.docx]
